# Delineating mouse β-cell identity during lifetime and in diabetes with a single cell atlas

**DOI:** 10.1038/s42255-023-00876-x

**Published:** 2023-09-11

**Authors:** Karin Hrovatin, Aimée Bastidas-Ponce, Mostafa Bakhti, Luke Zappia, Maren Büttner, Ciro Salinno, Michael Sterr, Anika Böttcher, Adriana Migliorini, Heiko Lickert, Fabian J. Theis

**Affiliations:** 1 Institute of Computational Biology, Helmholtz Zentrum München, Neuherberg, Germany; 2 TUM School of Life Sciences Weihenstephan, Technical University of Munich, Freising, Germany; 3 Institute of Diabetes and Regeneration Research, Helmholtz Zentrum München, Neuherberg, Germany; 4 German Center for Diabetes Research (DZD), Neuherberg, Germany; 5 Medical Faculty, Technical University of Munich, Munich, Germany; 6 Department of Mathematics, Technical University of Munich, Garching, Germany; 7 Genomics and Immunoregulation, Life & Medical Sciences (LIMES) Institute, University of Bonn, Bonn, Germany; 8 Systems Medicine, Deutsches Zentrum für Neurodegenerative Erkrankungen (DZNE), Bonn, Germany; 9 McEwen Stem Cell Institute, University Health Network (UHN), Toronto, Ontario Canada

**Keywords:** Data integration, Data mining, Mechanisms of disease, Transcriptomics, Diabetes

## Abstract

Although multiple pancreatic islet single-cell RNA-sequencing (scRNA-seq) datasets have been generated, a consensus on pancreatic cell states in development, homeostasis and diabetes as well as the value of preclinical animal models is missing. Here, we present an scRNA-seq cross-condition mouse islet atlas (MIA), a curated resource for interactive exploration and computational querying. We integrate over 300,000 cells from nine scRNA-seq datasets consisting of 56 samples, varying in age, sex and diabetes models, including an autoimmune type 1 diabetes model (NOD), a glucotoxicity/lipotoxicity type 2 diabetes model (db/db) and a chemical streptozotocin β-cell ablation model. The β-cell landscape of MIA reveals new cell states during disease progression and cross-publication differences between previously suggested marker genes. We show that β-cells in the streptozotocin model transcriptionally correlate with those in human type 2 diabetes and mouse db/db models, but are less similar to human type 1 diabetes and mouse NOD β-cells. We also report pathways that are shared between β-cells in immature, aged and diabetes models. MIA enables a comprehensive analysis of β-cell responses to different stressors, providing a roadmap for the understanding of β-cell plasticity, compensation and demise.

## Abstract (web summary)

This study reports the mouse islet atlas, a curated resource integrating scRNA-seq data of over 300,000 cells from nine datasets, covering pancreas development, homeostasis and disease states.

## Main

The major hallmark of diabetes mellitus is impaired glucose homeostasis. Blood glucose is regulated by multiple hormones secreted from pancreatic islets of Langerhans that consist of insulin-producing β-cells, which are main acters in diabetes, as well as glucagon-producing α-cells, somatostatin-producing δ-cells, pancreatic polypeptide-producing γ-cells and ghrelin-producing ε-cells^1^. Type 1 diabetes (T1D) and type 2 diabetes (T2D) arise due to the loss or progressive dysfunction of β-cells, respectively. Current anti-diabetic medications do not lead to remission, whereas more-effective treatments, such as bariatric surgery and islet transplantation, are highly invasive or can be only offered to a small number of patients^2–4^. The central role of β-cells in diabetes development urges the establishment of new therapies that focus on restoring β-cell mass and function^4,5^. Achieving such strategies requires a deeper understanding of β-cell heterogeneity, maturation, function and failure^6–8^.

Shortly after birth, β-cells are immature, defined by poor glucose-stimulated insulin secretion (GSIS)^9^. Immature β-cells gain functional maturation, as defined by the expression of several protein markers, including Urocortin-3, Flattop, transcription factor MafA and glucose transporter encoded by *Slc2a2* (also known as *Glut2*) and accurate GSIS in the first weeks after birth and again after weaning^9–12^. Adult β-cells also differ within and across phenotypes and conditions^7,11^. For instance, insulin production and secretion of β-cells are changed due to healthy aging or stress-induced senescence^13–17^. The function also differs between sexes, with male β-cells having transcriptomic signatures more akin to T2D^18^.

Different stressors can lead to β-cell failure, which is often studied with mouse models^19,20^. T2D is marked by gluco-/lipotoxicity leading to β-cell dedifferentiation, compensatory insulin production and resulting endoplasmic reticulum (ER) stress^21,22^, all of which are also present in the hyperphagic mouse db/db model^23,24^. In contrast, T1D is caused by autoimmune attack against β-cells^25,26^ that is mirrored by the mouse non-obese diabetic (NOD) model, which was also used to show the importance of β-cell stress-induced senescence and senescence-associated secretory phenotype in T1D^20,27^. β-cell identity can also be disrupted due to chemical stress^28^ and the streptozotocin (STZ)-induced ablation of β-cells was previously used to study both T1D and T2D^29–31^. Yet, due to failed clinical translation of treatments showing promise in animal models, it is important to decipher to which extent models resemble human diabetes^25^.

The implication of single-cell RNA sequencing (scRNA-seq) has greatly enhanced our understanding of β-cell maturation, heterogeneity and function in health and disease^1,30,32–35^. Nevertheless, there is no consensus on which β-cell populations exist^6,8,36^ and which pathways lead to β-cell dysfunction in different conditions. For example, for T2D progression alone, previous studies used different systems and individually identified various molecular changes, associated with energy metabolism, compensatory insulin secretion, apoptosis, inflammation, dedifferentiation and disrupted islet communication^32,37,38^. This ambiguity can be attributed to heterogeneous cellular states, joint action of multiple molecular mechanisms, different stressors and confounding of unknown environmental factors^26,32,35,38,39^. Such complexity cannot be fully captured in datasets of individual studies. Hence, a combined analysis of multiple datasets is needed to comprehensively describe β-cell heterogeneity in health and disease and to disentangle molecular pathways contributing to the deterioration of glucose homeostasis in various dysfunction conditions.

**Fig. 1:**
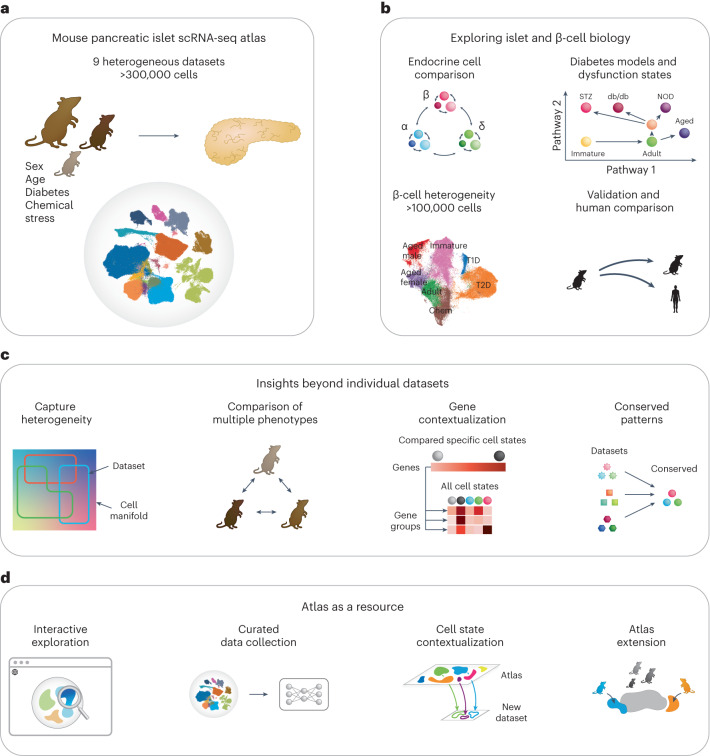
The MIA of scRNA-seq datasets across conditions offers new insights into islet and β-cell biology. **a**, MIA content, including different conditions: sex, age, diabetes models (STZ, db/db and NOD) and anti-diabetic treatments and chemical stress (application of different chemicals such as FoxO inhibitor). **b**, Putative new biological insights. **c**, Analyses enabled by MIA that would not have been possible on individual datasets. **d**, Potential use cases of MIA as a resource for future studies.

Direct comparison of multiple scRNA-seq datasets generated by different scientific groups is often not possible due to batch effects. To circumvent this, multiple scRNA-seq data analysis and integration^40–43^ approaches have been proposed. This also enabled the creation of so-called ‘integrated atlases’ that provide an expertly curated resource with a high-quality embedding optimized to retain biological variation, while removing batch effects. Atlases have become an invaluable tool as they provide new insights beyond individual datasets, such as the description of the cellular landscape in health and disease, and comparison across animal or in vitro models and corresponding human datasets^44–46^. While previous efforts have been made to compare the results of multiple islet scRNA-seq studies^18,35,47^, a comprehensive integrated atlas of mouse pancreatic islet cells across biological conditions and datasets with sufficient power to identify cell states is still missing. Therefore, we present the integrated MIA of scRNA-seq datasets across conditions (Fig. 1a). The analysis of MIA provided insights that could not be obtained from individual datasets (Fig. 1c), including a holistic description of the β-cell landscape across datasets and conditions, identification of similarities and differences between diabetes models and disentanglement of molecular pathways involved in different types of β-cell dysfunction (Fig. 1b). To empower future studies we also made MIA available for both interactive and computational analyses (Fig. 1d; https://github.com/theislab/mouse_cross-condition_pancreatic_islet_atlas).

## Results

### An integrated atlas of mouse pancreatic islet cells across conditions

**Fig. 2:**
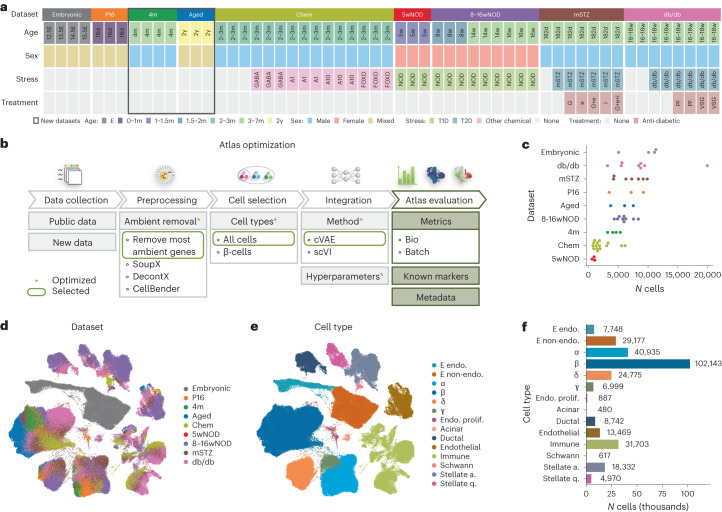
The integrated MIA captures cell types and states across lifetime, sexes and multiple stressed or diabetic conditions from different scRNA-seq datasets. **a**, Metadata of datasets and samples used in MIA. **b**, Overview of atlas integration evaluation. We tested multiple integration approaches and used the circled ones for the final atlas. **c**, Number of cells per sample (dots) within each dataset. **d**, Dataset distribution within the integrated atlas (excluding low-quality cells) shown on a UMAP. Datasets are described in Table 1. **e**, Atlas-level cell-type re-annotation (excluding low-quality cells) shown on a UMAP. **f**, Number of cells per cell type from atlas-level re-annotation, excluding low-quality cells. E, embryonic; P, postnatal; d, days; w, weeks; m, months; y, years; A1/A10, artemether (1 or 10 μM); FOXO, FoxO inhibitor; G, GLP-1; e, estrogen; i, insulin; PF, pair-fed; VSG, vertical sleeve gastrectomy; endo., endocrine; prolif., proliferative; stellate a., stellate-activated; stellate q., stellate-quiescent.

To better understand what the transcriptome of individual healthy pancreatic islet cells looks like and how it changes across a lifetime and upon various forms of diabetogenic stress, we integrated nine mouse datasets. We comprehensively collected seven previously published datasets (Methods describe data inclusion criteria) and generated two new datasets (Table 1). MIA contains 301,796 pancreatic islet cells from 56 samples (Fig. 2a,c, Table 1 and Supplementary Table 1). We use the term dataset for the collection of samples that were generated for the same purpose (for example, published together) and the term sample for jointly processed cells with shared biology, which may originate from a single animal, sequenced individually or demultiplexed, or are pooled across multiple animals sequenced on the same lane without demultiplexing. The samples within MIA vary in sex, age (ranging from embryonic to postnatal, to adult, to aged), application of chemical stressors implicated in the loss of cellular identity (FoxO inhibitor and artemether) and disease status (diabetes models, NOD, db/db and multiple low-dose STZ (mSTZ) together with different anti-diabetic treatments (vertical sleeve gastrectomy (VSG), insulin, glucagon-like peptide 1 (GLP-1) and estrogen) (Fig. 2a). To cover a wide range of developmental stages we extended the available scRNA-seq data (embryo to adult) with a newly generated scRNA-seq of aged mice (>2 years) across sexes (17,361 cells). To identify characteristics of mature cells conserved across datasets we sampled islet cells from adult (4-month-old) male mice (17,353 cells), thus complementing two other publicly available datasets.

**Table 1:** Summary of datasets used for the atlas and their availability. For detailed sample information, including sex, please refer to Supplementary Table 1

Name	Description	*N* samples	GEO accession	Reference	Source	Ensembl release
Embryonic	Embryo progression from E12.5 to E15.5	4	GSE132188	^60^	In-house	100
P16	Healthy young (P16) islets sorted according to the Fltp lineage-tracing model	3	GSE161966	^79^	In-house	94
4m	Healthy adult (4-month-old) islets from pancreas head and tail sorted according to the Fltp Venus reporter to isolate FVR^+^ and FVR^−^ cells	4	GSE211796	Previously unpublished	In-house	94
Aged	Healthy aged (2-year-old) islets sorted according to the Fltp lineage-tracing model	3	GSE211795	Previously unpublished	In-house	94
mSTZ	Healthy adult control, mSTZ-induced T2D model and mSTZ model with different anti-T2D treatments	7	GSE128565	^30^	In-house	100
db/db	Healthy adult control, db/db-induced T2D model and db/db model with different anti-T2D treatments	8	GSE174194	^23^	In-house	94
5wNOD	NOD model of T1D before T1D onset (5 weeks)	3	GSE144471	^176^	External	100
8–16wNOD	NOD model of T1D during T1D development (8–16 weeks)	9	GSE117770	^27^	External	100
Chem	Healthy young adult control or with applied chemical stress; sequencing with spike-in cells	15	GSE142465 (GSM4228185 to GSM4228199)	^28^	External	100

GEO, Gene Expression Omnibus.

**Extended Data Fig. 1:**
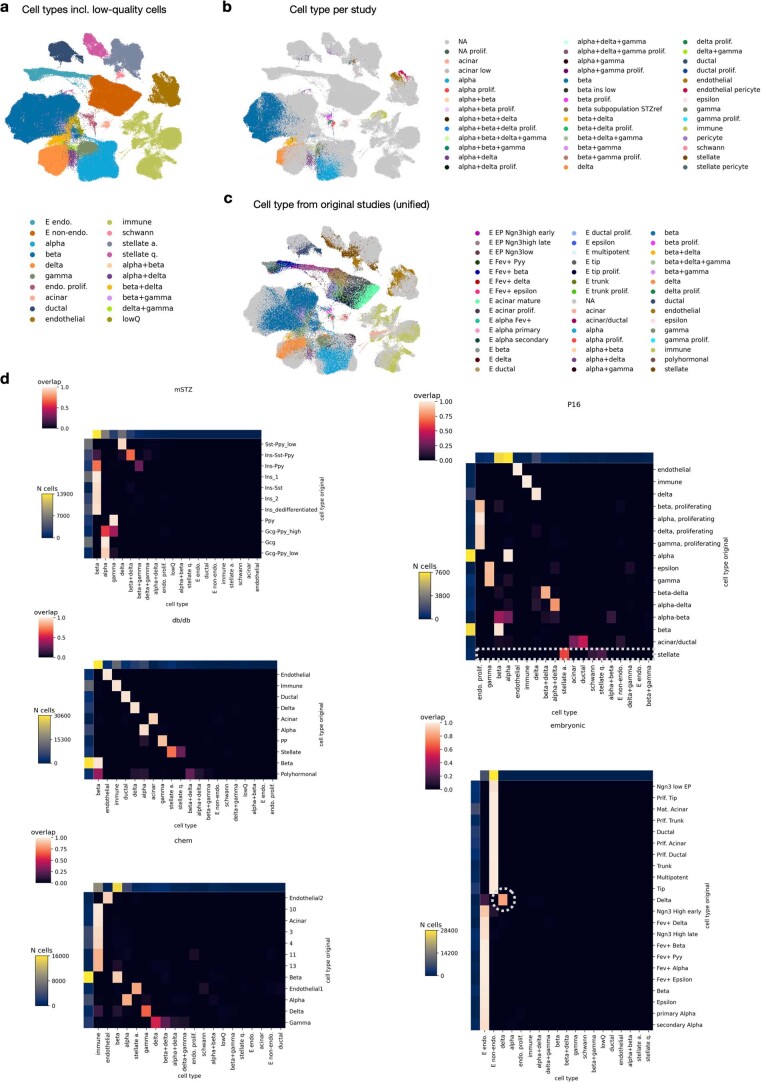
Comparison of cell types assigned in original studies and in integrated atlas re-annotation. (**a**) Atlas-level cell type re-annotation within the atlas shown on UMAP, including low-quality and potential doublet cells. (**b**) Cell types used for integration evaluation. Annotation was performed for selected samples (colored in cells) per study; unannotated cells are marked with NA. Some cell types were later renamed for the final atlas annotation (for example, the annotations in panel **b** contain the name pericyte which was later in panel **a** corrected to stellate activated). (**c**) Cell types as reported in the original publications. Cell type names were unified across studies and cells with missing annotation are marked with NA. (**d**) Comparison of integration-based re-annotated and previously reported cell type labels. Datasets that did not have previously reported annotation are not shown. Overlaps were normalized per previously reported cell type. In the P16 dataset, the dotted rectangle indicates rare Schwann cells that were merged with a larger population of stellate cells in the original annotation. In the embryonic dataset, the dotted circle indicates the mapping of embryonic δ-cells to the postnatal δ-cells cluster. Abbreviations: ‘+’ - potential doublet, lowQ - low-quality, EP - endocrine progenitor/precursor, prlf. - proliferative, mat. - mature.

To enable joint analysis of all datasets we performed data integration, creating a joint embedding space. We ensured optimal trade-off between batch correction and biological preservation on the level of cell types and cell states by evaluating different integration approaches, including preprocessing and data selection, integration tools and hyperparameter selection (Fig. 2b), as discussed in Supplementary Note 1. The integrated atlas shows clear separation into clusters that correspond to distinct cell types (Fig. 2e and Extended Data Fig. 1a–c) that colocalize across datasets (Fig. 2d).

**Extended Data Fig. 2:**
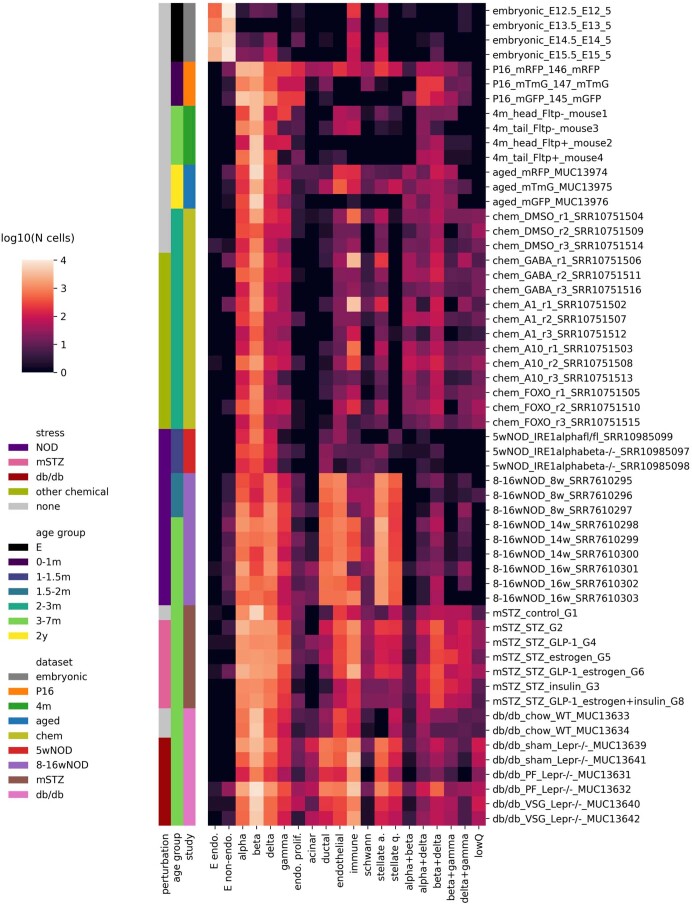
Number of cells per cell type in each sample. Sample names are given as study_sampleDescription_sampleIdentifier. Some of the datasets contained samples enriched for endocrine cells (Supplementary Table 1), which prevents direct cell type proportion comparison between samples with different cell sorting.

As the available cell type annotation was incomplete and inconsistent across datasets (Extended Data Fig. 1c,d) we manually re-annotated the integrated embedding (Fig. 2e,f and Extended Data Fig. 1a). This enabled us to resolve cell populations that were not annotated in some of the original studies, potentially because low cell numbers hamper annotation^48^. For example, we found that Schwann cells (617 out of 301,796 atlas cells) were present across the studies (Extended Data Fig. 2), although they were not annotated in any individual dataset (Extended Data Fig. 1d). Similarly, none of the original annotations distinguished between activated and quiescent stellate cells and some of the studies did not annotate stellate cells at all (Extended Data Fig. 1d and Extended Data Fig. 2).

Additionally, we also observed populations influenced by technical artifacts that colocalized across datasets, namely a low-quality cluster (lowQ, 853 cells, as well as low-quality cells identified based on a more detailed analysis of individual cell type clusters, 2,782 cells within β-cell cluster and 377 cells within α-cell cluster) and mixed (doublet) clusters (altogether 9,966 cells) (Extended Data Fig. 1a and Supplementary Table 2). They may be useful in the future in automatic annotation transfer to identify residual low-quality populations in new datasets, such as doublets that are often hard to identify.

### Embryonic and postnatal endocrine cell type markers partially overlap

Pancreatic islet profiling and stem cell differentiation highly depend on reliable endocrine cell type markers^49^; however, markers of individual cell types may differ across developmental stages. For example, in embryonic and postnatal stages different cell types are present, meaning that different markers will be specific for an individual cell type against all other present cell types. Furthermore, our integrated embedding revealed molecularly distinct cell states within cell types across development (Fig. 2d and Extended Data Fig. 1). Thus, we provide cell-type-specific markers separately for embryonic and postnatal mice (Supplementary Table 3). We did not compute postnatal ε-cell and embryonic γ-cell markers due to the lack of these cell types at the respective stages.

**Extended Data Fig. 3:**
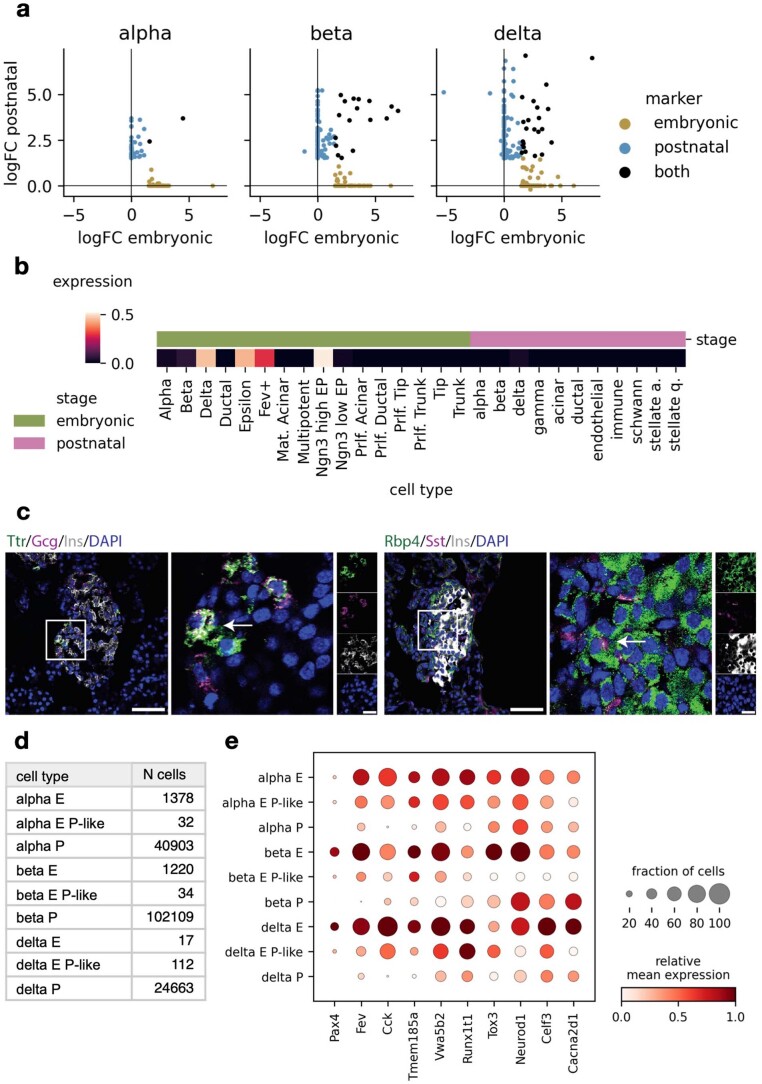
Endocrine markers differ in embryonic and postnatal datasets. (**a**) Comparison of endocrine markers in embryonic and postnatal data, showing whether genes were selected as potential markers in each stage (color). Genes missing from a stage-specific DGE analysis were assigned a logFC of 0. (**b**) Expression of *Cer1* across embryonic cell types (original study annotation) and postnatal cell types (atlas-level re-annotation). (**c**) Validation of selected endocrine markers with immunohistochemistry. Arrows indicate Ttr and Gcg double-positive α-cells (left) and Rbp4 and Sst double-positive δ-cells (right). The first subplot in the row for either Ttr or Rbp4 experiment shows a zoomed-out section, with the square denoting the zoomed-in region that is shown on the rest of the subplots, with an overlay of channels in the middle and individual channels on the right. Scale bars represent 50 μM for zoomed-out images and 20 μM for zoomed-in images. The images are representative examples from the analysis of three independent animals. (**d**) Number of cells in each endocrine cell group. Cell groups are as in Fig. 3. (**e**) Expression of embryonic Fev+ EP markers from Bastidas-Ponce et al. (2019) across endocrine cell groups.

The identified embryonic and postnatal markers only partially overlapped (Extended Data Fig. 3a), confirming that distinct marker sets are needed at different developmental stages. For example, while the expression of *Cer1* is higher in embryonic compared to postnatal δ-cells, it is a potential δ-cell marker only in postnatal and not in embryonic samples. This is due to the high expression of *Cer1* also in ε-cells and high-level *Ngn3*-expressing endocrine precursor cells that are present only in the embryo (Extended Data Fig. 3b).

**Fig. 3:**
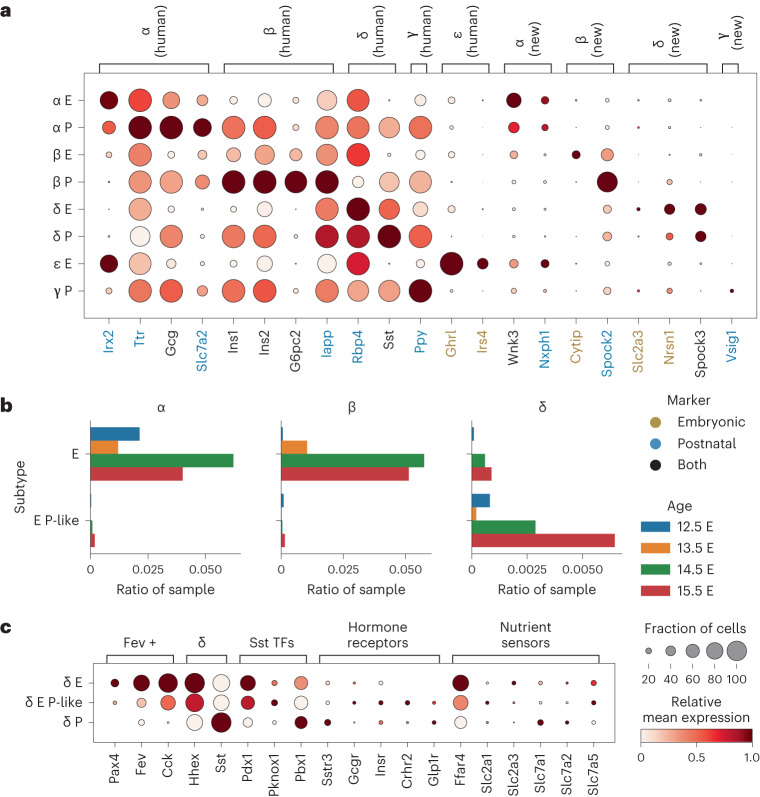
The integrated atlas embedding shows differences between embryonic and postnatal endocrine cells. **a**, Expression of endocrine markers shown across postnatal (P) and embryonic (E) endocrine cell types, including known markers shared with human (labeled human) and newly identified markers (labeled new). **b**, Number of cells in each embryonic endocrine cell group within individual embryonic samples, expressed as a fraction of cells within a sample. Cell groups are E, embryonic cells mapping to the embryonic cluster; and E P-like, embryonic cells mapping to the postnatal cluster. **c**, Expression of known maturity and δ-cell function markers across embryonic δ-cells groups. Groups are as in **b**: P, postnatal cells mapping to the postnatal cluster. In **a** and **c**, relative expression is computed as the average of cell groups normalized to [0,1] for each gene feature.

Some of the markers were shared with human endocrine markers reported in a recent scRNA-seq meta-analysis^49^ (mouse homologs *Ttr*, *Gcg*, *Irx2* and *Slc7a2* for α-cells; *Ins1*, *Ins2*, *G6pc2* and *Iapp* for β-cells; *Sst* and *Rbp4* for δ-cell; *Ppy* for γ-cells; Fig. 3a) and in other publications (*Ghrl* and *Irs4* for ε-cells)^50,51^. Furthermore, we detected several new cell-type-specific genes at different developmental stages (for example, *Wnk3* and *Nxph1* for α-cells; *Cytip* and *Spock2* for β-cells; *Slc2a3*, *Nrsn1* and *Spock3* for δ-cells; *Vsig1* for γ-cells; Fig. 3a). Among these, *Spock3* has been reported multiple times as a human α-cell, rather than δ-cell marker^49,52,53^; however, in mice, we observed consistent upregulation in δ-cells across datasets, which is further supported by a previous study reporting this gene as a δ-cell marker in zebrafish^54^.

We analyzed the protein expression of two transcriptome-based markers (*Ttr* in α-cells and *Rbp4* in δ-cells) with immunohistochemistry in mouse islets (Extended Data Fig. 3c). As anticipated, the expression of Ttr protein, which is involved in the regulation of Gcg expression and glucose homeostasis^55^, was specific to α-cells. In contrast, Rbp4 protein, which was previously reported to be a marker of δ-cells^49,56^, is expressed across the whole islet and could thus not be used to reliably distinguish δ-cells in immunohistochemistry (Fig. 3a and Extended Data Fig. 3c). Its relatively high protein levels in β-cells may be further explained by the young developmental stage (P9) of the used islets and hence β-cell immaturity, which is known to be associated with high Rbp4 expression^57,58^.

### Embryonic δ-cells cluster with postnatal δ-cells

One of the key questions in islet biology is when and how endocrine cells become functionally mature, which is of relevance for developing functional cell types from pluripotent stem cells^1^. As MIA provides a shared embedding of different biological conditions from multiple datasets that would otherwise not have been comparable due to confounding batch effects, we leveraged it to analyze cell populations during endocrine maturation. As expected, most embryonic cells (termed E group) generally did not overlap with postnatal cells (termed P group), but notably we observed that a large proportion of embryonic δ-cells mapped to the postnatal δ-cell cluster (termed E P-like group; Fig. 3b and Extended Data Figs. 1d and 3d).

To understand this overlap, we evaluated the expression of endocrine development and δ-cell function-related genes. The E P-like δ-cells had, in comparison to the E group, lower expression of δ-cell lineage determinant *Hhex*^59^ and lower expression of gene markers enriched in the Fev-positive population^60^, from which δ-cells arise^60–63^ (Fig. 3c and Extended Data Fig. 3e). Among known δ-cell functional genes, somatostatin was highly expressed already in the E group, likely because *Sst* has been used for δ-cell annotation, therefore not capturing earlier δ-cell developmental stages^50^. Other functional genes encode transcription factors involved in *Sst* gene expression^64^ and genes encoding sensors required for appropriate paracrine regulation, namely neurotransmitters, hormone receptors, including the somatostatin receptor (*Sstr3* gene) (autocrine feedback) and genes encoding nutrient sensors, including sensors for milk-based high-fat weaning diet (fatty acids, *Ffar4* gene; amino acids, SLC7 family)^56,65–69^ (Fig. 3c). They were relatively highly expressed in all cell groups. This indicates that δ-cells already possess the machinery for regulating somatostatin expression at the embryonic stage and that they quickly downregulate the expression of developmental genes, explaining the mapping of embryonic δ-cells to the postnatal cluster. However, we must note that genes potentially involved in somatostatin regulation could also be related to other cellular functions at this developmental stage. Thus, further validation of δ-cell physiology during development would be required.

### β-cells show heterogeneity across and within conditions

Extensive research has shown that β-cells are heterogeneous^7,9,11^; however, there is a lack of knowledge on how these states relate^6,8^. Hence, we aimed to use MIA to comprehensively describe β-cell states alongside their molecular characteristics in different sexes, ages and stress conditions (Table 1).

**Fig. 4:**
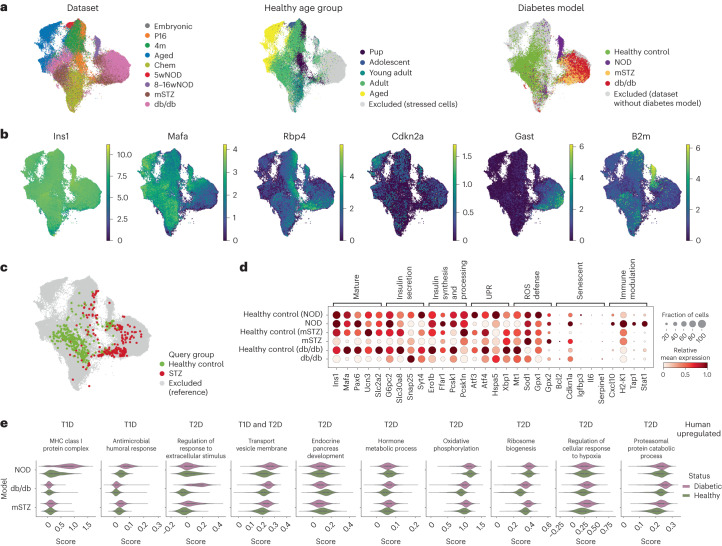
The integrated atlas embedding reveals similarities between mSTZ and db/db diabetes models. **a**, Distribution of technical (dataset) and biological (age, disease status) covariates on a UMAP of the β-cell MIA subset. The age subplot shows only cells from healthy, non-stressed samples. The disease subplot shows only cells from samples belonging to datasets that contain both healthy and diabetes model data. **b**, Expression of selected β-cell heterogeneity markers on a UMAP of the β-cell MIA subset. **c**, Joint UMAP embedding of the reference atlas (background) and the external (Feng) mouse dataset (query, foreground) indicating positioning of healthy control and STZ-treated query cells. **d**, Expression of known β-cell function genes across different diabetes models and corresponding healthy controls from individual datasets (the NOD model is from the 8–16wNOD dataset, other model names correspond to the dataset names). Relative expression is computed as the average of cell groups normalized to [0,1] for each gene feature. **e**, Activity of gene sets upregulated in T1D or T2D human samples shown for mouse diabetes models and corresponding healthy controls from individual datasets (as in **d**). On the overlay boxplots the white dot represents the median, the box the quartiles and the whiskers the minimum and maximum (no cells qualified as outliers). The data sizes are (reported as ‘*N* samples (*N* cells)’), NOD_elimination diabetic 6 (3,191) and healthy 3 (548); STZ diabetic 1 (1,496) and healthy 1 (5,795); VSG diabetic 2 (5,264) and healthy 2 (7,706). Each sample contains islets from multiple mice. MHC, major histocompatibility complex.

**Extended Data Fig. 4:**
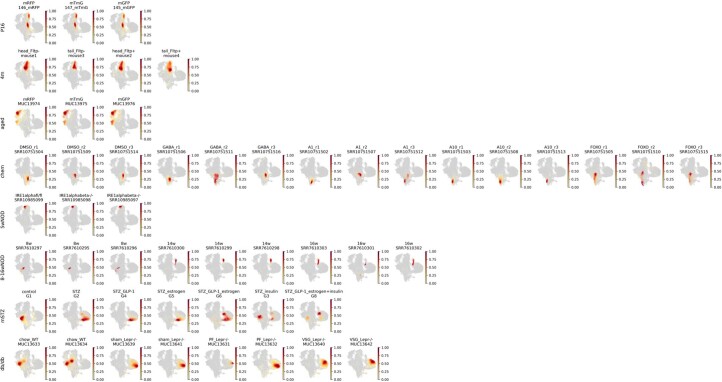
Integrated embedding of β-cells from individual samples corresponds to biological conditions. The distribution density of cells from each sample on a UMAP of the β-cell atlas subset. Sample names are reported with the sample description and identifier. The embryo dataset is not shown due to a small number of cells within the β-cell cluster.

To test whether the integration is adequate for downstream analyses of β-cell states we assessed a MIA subset consisting of 102,143 β-cells. Cells separated on the embedding based on biological covariates, such as age and disease status and overlapped between samples with similar biological covariates from different datasets (Fig. 4a and Extended Data Fig. 4). For example, healthy control β-cells mapped together regardless of their dataset of origin (mSTZ, db/db and 8–16wNOD), whereas the cells from diabetic samples from these datasets mapped away from the healthy clusters. This is in accordance with previously reported β-cell changes in aging and diabetic dysfunction^6,70,71^. Furthermore, we assessed the expression patterns of known immaturity (*Rbp4*), maturity (*Mafa*), stress (*Gast*), aging/senescence (*Cdkn2a*) and inflammatory (*B2m*) β-cell transcriptomic markers (Fig. 4b), showing complementary patterns when considering opposite activity of β-cell functional maturation (*Mafa*) and dedifferentiation (*Gast*) markers. Altogether, this indicates successful integration of the datasets both on the cell-type and cell-state level.

### Transcriptomic similarity of db/db and STZ diabetes model β-cells

The usage of the appropriate mouse model is of utmost importance to studying β-cell function both in healthy and disease conditions^19^. Different models with unique phenotypes and disease mechanisms have been developed^20^, each of them with advantages and limitations to be considered^19^. To better understand the transcriptomic differences among the diabetes mouse models, we compared the commonly used genetic models of T1D (NOD, for which we used samples from early disease stages^20,27^) and T2D (db/db^24^) together with the β-cell ablation model (STZ) that was previously used to study both T1D and T2D^29,30^. The NOD model is characterized by autoimmune and cytokine-mediated destruction of β-cells as well as ER stress^72,73^. The leptin-receptor-deficient db/db mice are obese, hyperglycemic and dyslipidemic^74,75^, leading to β-cell failure and compensation, which are associated with metabolic stress, including ER stress^23,24^. The STZ treatment is used for specific destruction of β-cells due to its affinity for the Slc2a2 (ref. ^76^) protein expressed in β-cells. The stressor is applied either in a single high dose to resemble T1D or in multiple low doses to elicit partial β-cell loss reminiscent of T2D, but in the absence of insulin resistance^19^, with both strategies analyzed below.

Based on MIA embedding, we found that β-cells from mSTZ-induced (multiple low doses) and db/db models mapped together, separately from NOD diabetic β-cells (Fig. 4a). To further validate the similarity between the mSTZ and db/db models, we mapped onto MIA another mouse dataset (referred to as the Feng dataset^31^, not part of MIA), containing samples treated with STZ (single high dose). Again, the healthy control cells from the Feng study mapped onto the healthy β-cell region of MIA and STZ-treated cells mapped onto the region with mSTZ and db/db model samples (Fig. 4c). Similarly, in the future mapping onto MIA may reveal relationships between other dysfunctional conditions.

To better understand molecular mechanisms underlying β-cell dysfunction within each of the models, we analyzed the expression of known β-cell function and stress genes (Fig. 4d). In the mSTZ and db/db models multiple maturity and insulin-related genes were downregulated, while in the NOD model immune modulation genes were upregulated. In all three models we observed expression changes in several unfolded protein response, reactive oxygen species defense and senescence-related genes. This indicates the involvement of metabolic stress in db/db and mSTZ models and immune stress in the NOD model, in accordance with current views on T1D and T2D pathomechanisms^77^.

To elucidate which mouse models capture transcriptional signatures of human T1D or T2D, we assessed whether changes observed in human diabetes are also present in mice. We performed differential gene expression (DGE) analysis on β-cells from multiple human T1D and T2D datasets (Table 2), selected genes upregulated across multiple datasets per diabetes type (T1D 32 genes, T2D 59 genes) and identified enriched gene sets (Supplementary Table 4). We further complemented our gene set list with known human diabetes-associated gene sets from the literature. Human T1D is marked by the upregulation of immune gene sets^21^, which were much more strongly upregulated in NOD than db/db and mSTZ models (Fig. 4e; details of gene set activity analysis across mouse models are provided in Supplementary Note 2). Conversely, human T2D is associated with changes in hormone metabolism and stress related to metabolic compensation^21,22,78^, which were upregulated in db/db and mSTZ but not in the NOD model. Thus, the mSTZ model reflects key molecular changes of human T2D, but not T1D. The presence of metabolic stress in the mSTZ model β-cells after clearance of the chemical stressor can be explained by the surviving population of β-cells being too small to prevent hyperglycemia and hence leading to compensatory insulin-production behavior and subsequent stress.

**Table 2:** Datasets used for validation, not part of the atlas. For detailed sample information, including sex, please refer to Supplementary Table 12

Species	Description	*N* samples	Technology	*N* cells (*N* β-cells)	GEO accession	Reference
Mouse	Healthy adult and aged islets	2	SMARTer	207 (207)	GSE83146	^177^
Mouse	Endocrine cells from healthy young and adult mice and adult mice treated with STZ or STZ and insulin, with samples collected at different times after STZ treatment	17	STRT-seq	2,999 (1,005)	GSE137909	^31^
Human	Islets from non-diabetic, T1D and non-diabetic islet autoantibody positive donors, including child donors	24	Chromium v2/v3	66,052 (11,298)	GSE148073	^26^
Human	Islets from non-diabetic and T2D adult donors	18	SMARTer Ultra Low RNA	1,600 (503)	GSE81608	^52^
Human	Islets from non-diabetic adult and aged donors	5	Chromium v2	26,474 (11,923)	GSE198623	^81^
Human	Islets from non-diabetic child and adult donors	8	Smart-seq2	2,282 (348)	GSE81547	^15^
Human	Islets from adult non-diabetic and T2D donors	8	SMARTer	617 (264)	GSE86469	^178^
Human	FACS-sorted islet cells from adult and aged donors with or without T2D	14	Smart-seq2	2,245 (674)	GSE124742 (FACS)	^21^
Human	Patch-seq of islet cells from adult and aged donors without diabetes, with T1D (adult only) or with T2D	53	Smart-seq2	2,319 (496)	GSE124742, GSE164875 (patch-seq)	^21,97^
Human	Islets from non-diabetic and T2D adult donors	9	Drop-seq	27,996 (9,958)	GSE101207	^78^
Human	Islets from non-diabetic child and non-diabetic and T2D adult donors	22	Smart-seq	619 (182)	GSE154126	^179^
Human	Islets from non-diabetic child and non-diabetic, T1D and T2D adult donors	9	Smart-seq	457 (111)	GSE83139	^22^

### Markers of β-cell states conserved across datasets

**Fig. 5:**
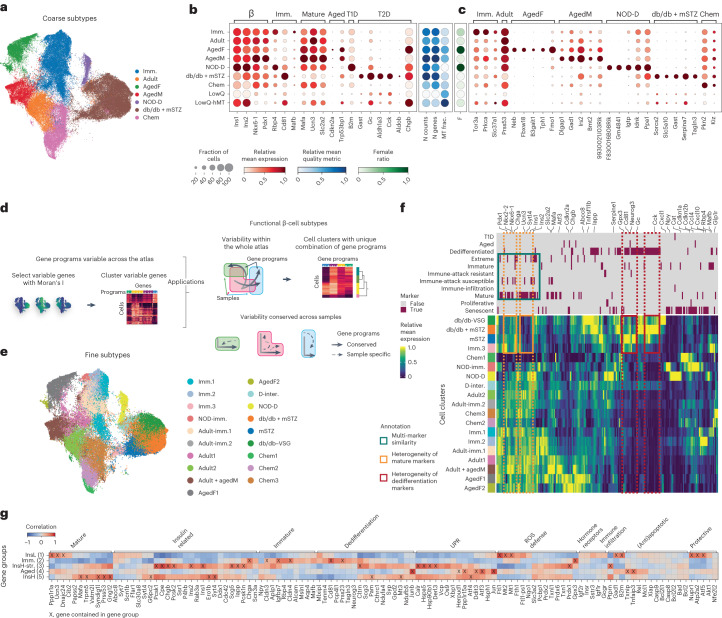
MIA encompasses β-cells heterogeneity across and within biological conditions. **a**, Coarse β-cell states labeled based on sample metadata (excluding low-quality clusters) shown as a UMAP. **b**, Expression of known markers (marker groups are specified on the top of the plot), quality control metrics and sex ratios across coarse β-cell states displayed in separate dot-plot panels. In the marker expression panel, the dot size indicates the fraction of cells expressing a gene, whereas in other panels it is set to a fixed size. **c**, Expression of MIA-based markers of coarse β-cell states. **d**, Overview of the method used for extraction of GPs and subsequent cell clustering resolution selection or definition of consistently variable GPs across samples. **e**, Fine β-cell states defined based on the presence of a unique combination of GPs (excluding low-quality clusters) shown as a UMAP. **f**, Expression of known β-cell heterogeneity markers across fine β-cell states. Phenotypes associated with individual genes (top). The dotted boxes represent two distinct sets of maturity (orange) and dedifferentiation or diabetes markers (red); the solid cyan box shows overlap and expression similarity between maturity, immune-attack susceptibility and extreme insulin producer markers. **g**, Correlation between gene groups variable in all healthy samples and known β-cell heterogeneity markers on the healthy β-cell subset. Markers present within a specific gene group are annotated with an X. imm., immature; M, male, F, female; NOD-D, NOD diabetic; D.-inter, diabetic intermediate; insL/H, insulin low/high; str., stressed. In **b**, **c** and **f**, relative expression is computed as the average of cell groups normalized to [0,1] for each gene feature.

**Extended Data Fig. 5:**
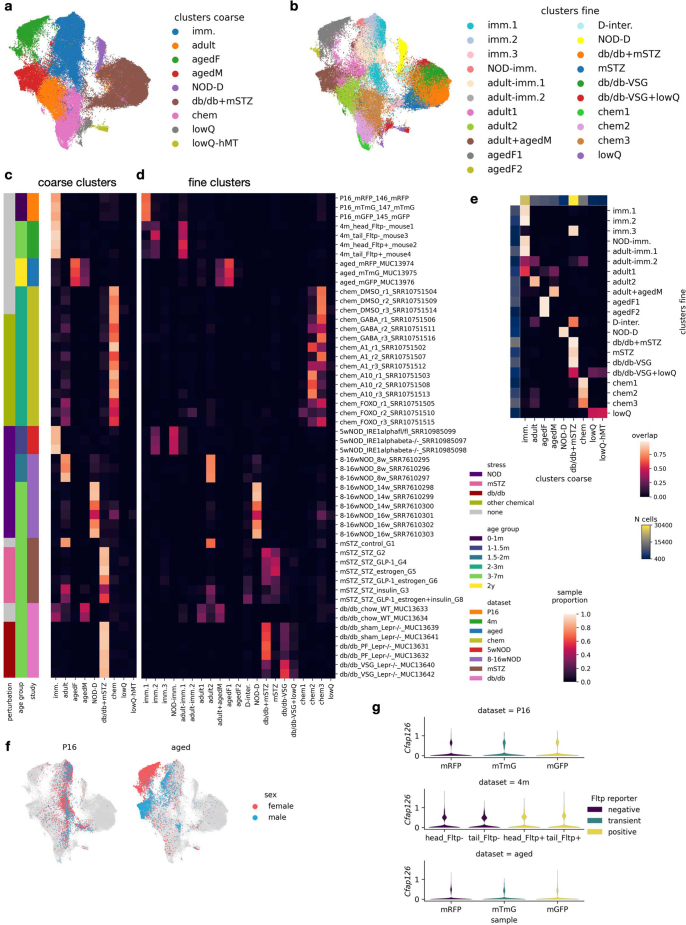
Different resolution β-cell states correspond to biological conditions. (**a**) Coarse β-cell states, including low-quality clusters. (**b**) Fine β-cell states, including low-quality clusters. (**c**) and (**d**) Coarse and fine, respectively, β-cell state proportions in each sample, also displaying corresponding sample metadata. Sample names are given as study_sampleDescription_sampleIdentifier. (**e**) Comparison of coarse and fine β-cell state annotation given as a normalized distribution of each fine state across coarse states. (**f**) A UMAP embedding of male and female β-cells across ages. Colored in are cells from datasets that have mixed sexes within samples, other β-cells are displayed as a background. (**g**) Expression of *Cfap126* (Flattop gene) across cell populations that were sorted based on the Flattop reporter system. Abbreviations: hMT - high mitochondrial transcript read fraction.

As it is unclear how newly reported β-cell states correspond across publications^6,7^, we next aimed to utilize the cross-dataset integrated conditions within MIA to describe β-cell heterogeneity in health and disease in a unified manner. We annotated states on postnatal non-proliferative β-cells (‘β’ cluster in Fig. 2e) and labeled them on the basis of the metadata (altogether referred to as ‘coarse states’; Fig. 5a and Extended Data Fig. 5a). We resolved populations of healthy adult, immature, aged (separated by sex), NOD diabetes model, mixed db/db and mSTZ diabetes models and cells from the dataset with chemical perturbations in cultured islets (referred to as chem) that likely separate due to strong differences in sample handling. For a detailed description of states see Supplementary Note 4.

We support the annotation of coarse states with known β-cell state markers depicted in Fig. 5b. Some known markers were not state-specific, such as certain immature marker genes that were also highly expressed in the db/db + mSTZ state (for example, *Cd81*; Fig. 5b), in accordance with β-cell dedifferentiation in mouse diabetes models^23,30,79^. Thus, the identification of new state-specific markers could improve the monitoring of β-cells in specific states to study their function. We identified markers specific for an individual β-cell state and conserved across all datasets mapping to that state, with top markers highlighted in Fig. 5c (Supplementary Table 5; a more detailed description is in Supplementary Note 4). For example, we identified a new marker of healthy adult state *Prss53*, associated with mitochondrial function^80^.

**Extended Data Fig. 6:**
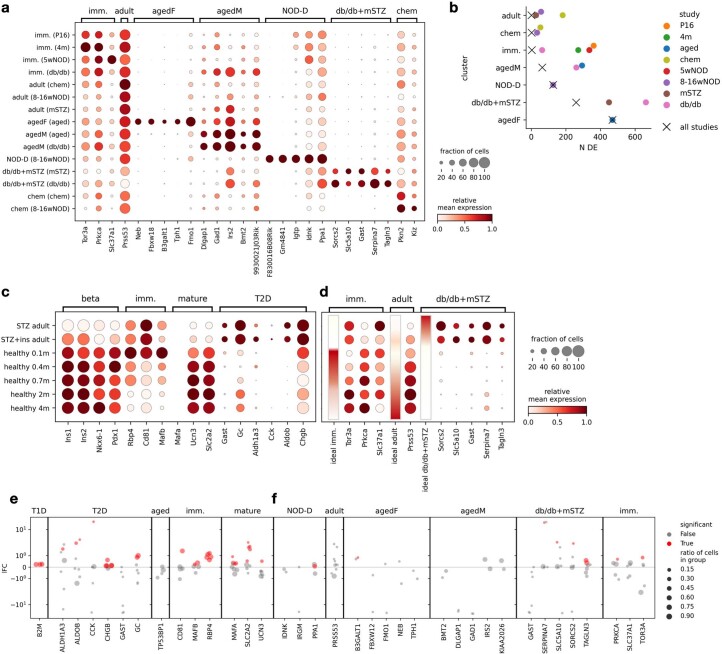
MIA-based β-cell markers are robust across mouse datasets, but do not directly translate to humans. (**a**) Expression of proposed β-cell state markers across coarse β-cell states per dataset (in brackets). (**b**) Number of cluster-specific markers extracted per dataset and state or as the intersection of all datasets within a state. (**c**) and (**d**) Expression of known and MIA-based, respectively, β-cell state markers on the external Feng mouse dataset. The ideal marker bar represents how we would expect markers of specific clusters to be expressed across the cell groups. (**e**) and (**f**) Translation of known and MIA-based, respectively, β-cell state markers to human datasets. In each dataset (dot) we compared marker expression within the relevant sample group to all other samples, showing a comparison lFC and statistical significance as well as the ratio of cells expressing the gene in the target group. Abbreviations: ins - insulin.

**Extended Data Fig. 7:**
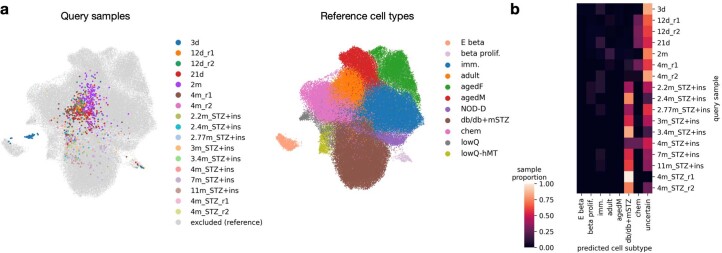
Reference mapping and cell state transfer for external mouse dataset β-cells reveals expected reference and query sample relationships. (**a**) Joint embedding of the atlas (reference) and the external mouse dataset (query). Left: All query samples, named as age, treatment and replicate when multiple samples with the same age and treatment were present. Right: Reference cell groups showing coarse β-cell states, as shown in Fig. 5a, and proliferative (part of the ‘endocrine proliferative’ atlas cluster) and embryonic β-cells (as annotated in the original study of the embryonic dataset). (**b**) Label transfer from the atlas to query, using cell groups as described in **a**. Cells with low label-transfer probability were assigned to the uncertain group. Shown are the ratios of each query sample predicted as a certain cell group.

To test the robustness of our markers we analyzed their expression on the Feng mouse dataset that is not part of the atlas^31^. This dataset consists of healthy young and adult mice, with multiple samples spanning the ages of 0.1–4 months, as well as STZ-treated diabetic samples (Extended Data Fig. 6c,d). The proposed T2D model state (db/db + mSTZ) and adult state markers were expressed as expected in the Feng dataset; however, we did not observe specific expression of immature markers in the young samples. We next evaluated whether this difference arises due to a different immature cell state present in the Feng dataset or due to technical issues in marker identification. Thus, we mapped Feng dataset cells to MIA. Indeed, we observed differences in the two immature cell states, as young samples from the Feng dataset did not map to MIA immature state (Extended Data Fig. 7a,b). The Feng postnatal day 3 (P3) β-cells mapped between embryonic and postnatal β-cells of MIA and the young postnatal cells (postnatal days 12 (P12) and 21 (P21)) mapped between the immature, adult and chem MIA states.

Additionally, we assessed whether previously known and MIA-based markers could be directly translated to ten human datasets with differences in donor metadata (Extended Data Fig. 6e,f). Only *B2m* (T1D marker)^27^ and *Rbp4* (immature marker)^79^ were significantly upregulated in all human samples associated with those phenotypes. This is in accordance with previous reports^81^ showing that not all mouse markers directly translate to human data.

### β-cell heterogeneity within biological conditions

β-cells are known to be heterogeneous within individuals^11,12,82^; however, our metadata-driven coarse states mainly did not reveal multiple populations per sample (Extended Data Fig. 5c). Some marker genes were heterogeneously expressed within coarse states, such as *Rbp4* in young and db/db + mSTZ states and *Mafa* and *Gast* in the db/db + mSTZ state (Fig. 4b), indicating that we could identify higher resolution states in MIA.

**Extended Data Fig. 8:**
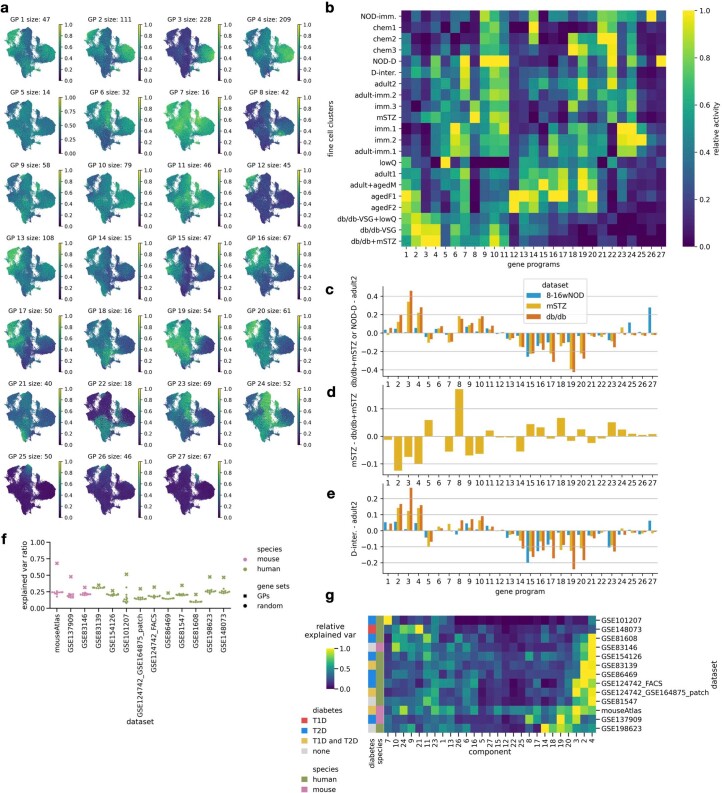
Activity of GPs across β-cells helps in β-cells state interpretation. (**a**) Activity of GPs on UMAP of the β-cell atlas subset. (**b**) Activity of GPs across fine β-cell states normalized per GP across states. (**c**), (**d**), (**e**) Differences in GP activity between pairs of fine β-cell states (specified on y axis) for individual datasets. (**f**) Ratio of variance explained by GPs across datasets compared to random groups of genes. (**g**) Relative variance explained by each GP, scaled as a ratio of maximal absolute value per dataset.

Annotation of cell states is challenging due to uncertainty about the number of distinct states^83^. To ensure that states can always be biologically interpreted, we based clustering on interpretable features (termed gene programs (GPs); Fig. 5d and Methods). GPs are data-driven groups of genes coexpressed across β-cells (27 GPs, 14–228 genes; Extended Data Fig. 8a and Supplementary Table 6). Most of the GPs were enriched for distinct molecular functions (Supplementary Table 6) and we show that they generalize to other datasets by explaining variance in two external mouse and ten human datasets (Extended Data Fig. 8f).

We defined 19 fine β-cell states (Fig. 5e), which mainly corresponded to subclusters of the coarse states (Extended Data Fig. 5e) and described more subpopulations within samples, while still containing cells from multiple samples and datasets (Extended Data Fig. 5d and Supplementary Table 2). Additionally, two clusters were characterized by low-quality control metrics and were thus not regarded as true cell states (Fig. 5e and Extended Data Fig. 5b). We further discuss β-cell heterogeneity captured within MIA in relation to previous literature in Supplementary Note 5.

We observed two populations of β-cells in the mSTZ model (states mSTZ and db/db + mSTZ; Fig. 5e and Extended Data Fig. 5d). We used biologically interpretable GP differences to ease the comparison of these two states (Extended Data Fig. 8b,d; for validation of this approach see Supplementary Note 6). The db/db + mSTZ state had higher activity of multiple GPs that contained known diabetes markers or were associated with ER stress (GP2, GP3 and GP4) and cell state mSTZ had higher activity of GPs associated with immaturity (GP8 and GP23). Both increased ER stress and immaturity were reported in the paper publishing the mSTZ dataset^30^; however, they did not describe dysfunctional populations differing in the two processes. While the more immature state (mSTZ state) was specific to the mSTZ model, the more stressed state (db/db + mSTZ state) also contained db/db model cells. This may be explained by either mSTZ diabetes model having a milder hyperglycemia than the db/db model^23,30^, leading to a lower β-cell compensatory response and thus reduced stress, or by a different mechanism of β-cell damage due to the use of STZ. As these two populations clearly differ in their metabolism, they may be of relevance for studying diabetes with the mSTZ model.

Publications based on individual datasets often do not agree on β-cell heterogeneity markers^35^. Thus, we used the wide range of β-cell phenotypes across datasets within MIA, encompassed by the fine β-cell states, to assess population markers manually extracted from the literature (Fig. 5f and Supplementary Table 7). Some markers previously reported as marking the same β-cell population, such as markers of maturity or dedifferentiation (often related to T2D models), separated into multiple groups with distinct expression patterns across fine states (Fig. 5f). This shows how MIA could be used to find specific and sensitive markers. Furthermore, we observed that different groups of markers reported across studies with different biological focuses share similar expression profiles, such as mature^10,23,84,85^, extreme insulin-producing^23,85^ and immune-attack-susceptible markers^86^. The immune-attack-susceptible markers were extracted by Rui et al.^86^ who reported NOD subpopulations differing in immune-attack susceptibility. They reported that the immune-attack-susceptible population expressed β-cell maturity genes and indeed we observed that the population markers reported by Rui et al. colocalized with known maturity genes in MIA (Fig. 5f). This demonstrates how the heterogeneous cell states within MIA can be used for gene contextualization by providing information on which β-cell states express a gene of interest and which known markers have similar expression patterns.

### β-cell dysfunction patterns within healthy samples

In our GP analysis we observed that GPs that changed between healthy and T2D model cells (GPs 3, 4, 19 and 20; Extended Data Fig. 8a,b) were also among GPs explaining the largest proportion of cell-to-cell variability within healthy datasets and samples in both mouse and human (Extended Data Fig. 8g and Supplementary Table 6). This motivated us to describe heterogeneity conserved across healthy adult samples.

**Extended Data Fig. 9:**
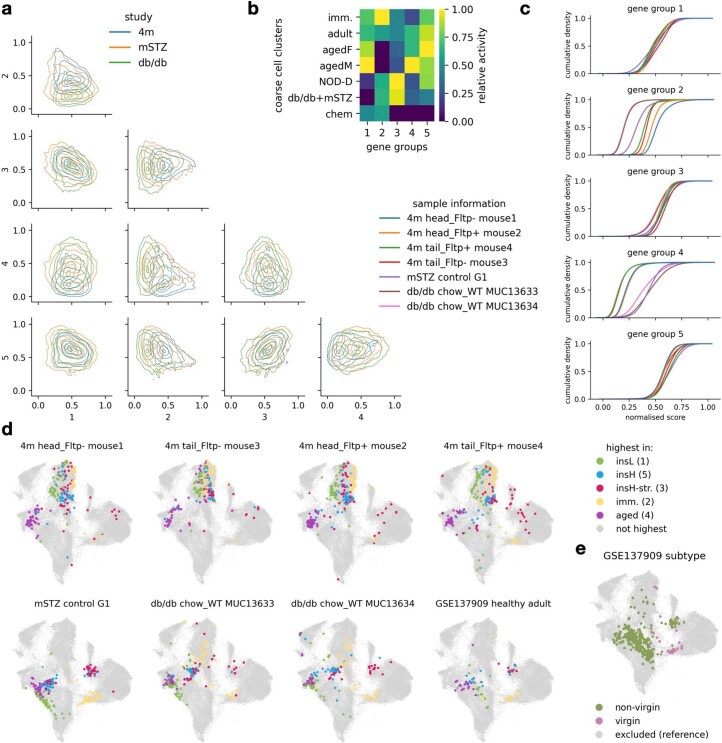
Healthy β-cells contain five distinct variable gene groups. (**a**) Pairwise comparison of the normalized activity of gene groups on β-cells from healthy samples shown as kernel-estimated density plots colored by study. Lines represent regions containing 5, 34, 67, 95 and 99% of cells. Axes represent the activity of the compared gene groups. (**b**) Mean gene group activity within coarse β-cell clusters, normalized across clusters. (**c**) Per-sample distribution of normalized gene group activities in β-cells shown as cumulative density functions. (**d**) Localization of cells with the highest activity of the five gene groups on the atlas β-cell UMAP for individual health adult atlas samples (named as: dataset sample_metadata sample_name) and the healthy adult samples from the external dataset (Feng, GSE137909) mapped on top of the atlas. (**e**) Localization of virgin and non-virgin β-cells from Feng (GSE137909) dataset on the integrated atlas UMAP as annotated in the original publication.

We collected genes that are consistently variable within individual healthy samples and grouped them based on coexpression patterns conserved across samples, resulting in five gene groups (a detailed description of groups is in Supplementary Note 7 and Supplementary Table 8). Groups 3 and 5 were associated with β-cell maturity and insulin production, with group 3 having a stronger insulin-production-related stress signature (Fig. 5g and Supplementary Table 8). Group 1 contained genes implicated in β-cell metabolic stress recovery, such as ATP production-related genes^82^ (Fig. 5g and Supplementary Table 8). The negative correlation between the expression of group 1 and groups 3 and 5 (Extended Data Fig. 9) is in accordance with previously reported cycling of β-cells between insulin production and recovery in mice and humans^82,87,88^. As group 1 genes, including multiple mitochondria-associated genes, β-cell maturation and function genes (*Ucn3*, *Ftl1*, *Cd63* and Scg2)^47,89^ and protective genes (*Nupr1*, *Atp2a2* and *Atf5*)^90–92^, are involved in healthy metabolic stress recovery they may be of interest for T2D therapy. Indeed, group 1 showed the lowest activity in the diabetes model β-cells (Extended Data Fig. 9b), indicating impaired stress recovery.

We also observed two gene groups indicating that cells within healthy adults differ in the degree of maturity and senescence. Group 4 contained senescence genes and healthy adult cells most highly expressing these genes colocalized with aged cells. Notably, while group 2 contained immaturity genes, the healthy adult cells with high expression of this group partially colocalized with the immature subset of mSTZ model cells (fine β-cell states imm.3 and mSTZ) (Supplementary Note 7, Fig. 5g, Extended Data Fig. 9 and Supplementary Table 8).

Comparison to a meta-analysis of human healthy heterogeneity markers^35^, revealed shared genes *Tm4sf4* and *Clu* from group 3 (insulin production and metabolic stress) and genes *Fos*, *Herpud1* and *Rgs4* from group 4 (aging). While these orthologs likely share function across species, Mawla and Huising^35^ did not specifically state which β-cell states they are associated with.

### Diabetes response of β-cells is highly complex

**Fig. 6:**
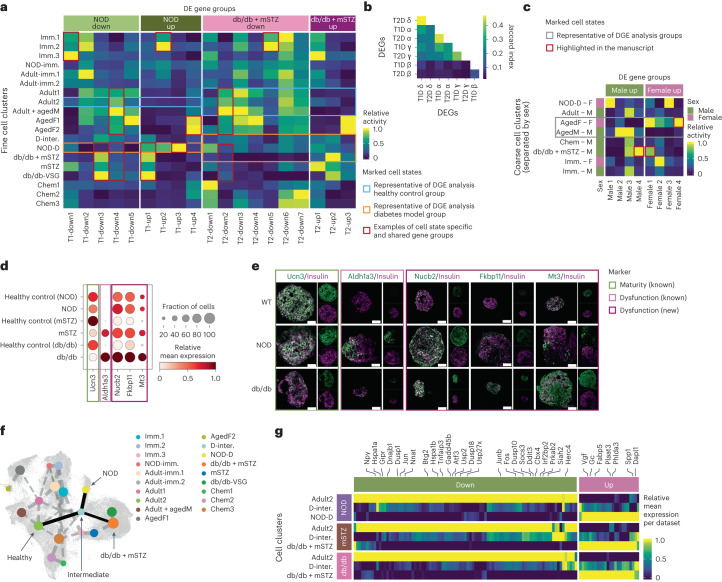
β-cell diabetes dysfunction involves different molecular patterns that are unique or shared with other conditions, including different diabetes models and aging. **a**, The activity of β-cell diabetes-trajectories (NOD and db/db + mSTZ) DEG groups across fine β-cell states (red rectangles mark examples highlighted in text). Cell groups representative of healthy and diabetic states in DGE analysis are marked with blue and orange rectangles, respectively. **b**, Overlap of DEGs across diabetes models (T1D NOD, T2D db/db + mSTZ) and endocrine cell types. **c**, Expression of DEG groups between aged males and females across coarse β-cell states, split by sex. Marked are cell groups highlighted in the text and groups representative of healthy and diabetes model cells from DGE analysis. **d**, Gene expression of diabetes markers that were validated on protein level; shown for diabetes models and associated controls. **e**, Validation of selected diabetes model β-cell DEGs on protein level with immunohistochemistry. The images are representative examples of three independent animals. Scale bars, 50 μM. For every antibody pair, the left plot shows an overlay of channels and the right shows individual channels. **f**, PAGA graph showing connectivity (lines) between fine β-cell states (dots) imposed on β-cell UMAP. The connections between healthy, intermediate and diabetes model states are marked in solid lines. **g**, Expression of DEGs with the same direction in NOD and db/db + mSTZ trajectories in healthy, intermediate and diseased states per dataset (dataset 8–16wNOD is abbreviated as NOD). Expression is normalized per gene and dataset. imm., immature; M, male; F, female; NOD-D, NOD diabetic; D.-inter, diabetic intermediate. In **a**, **c**, **d** and **g** relative expression is computed as the average of cell groups normalized to [0,1] for each gene feature.

While β-cells are the primary cell type affected in diabetes, the disease also has broader effects on the whole islet^93,94^. To investigate these effects, we performed DGE analysis between healthy and T1D model or T2D model samples in α-, β−, γ- and δ-cells. All cell types had a large number of differentially expressed genes (DEGs) in both diabetes types (Supplementary Fig. 2 and Supplementary Table 9). DEGs in the β-cell T1D model and T2D model had a relatively low overlap and were also distinct from DEGs in other cell types (Fig. 6b). This is in accordance with different mechanisms that lead to the loss or dysfunction of β-cells in T1D and T2D^77^. In contrast, DEGs overlapped more strongly between T1D model and T2D model within α-, γ- and δ-cells and also showed a relatively high overlap across these cell types. This is likely due to β-cells being the primary cell type affected in diabetes, further leading to islet disruption and causing residual stress in other endocrine cells^95,96^.

To characterize the residual stress within endocrine cell types other than β-cells we examined shared DEGs in both diabetes types. Upregulated genes were enriched for ER stress, whereas downregulated genes were enriched for gene sets related to membrane depolarization and ion transport (Supplementary Table 9) and contained hormone genes (*Gcg* in α-cells, *Ppy* in γ-cells and *Sst* in δ-cells) (Supplementary Table 9). This indicates that diabetes also affects endocrine hormone production and secretion in endocrine cell types beyond β-cells. In support of this, a recent human α-cell patch-seq study reported a loss of electrophysiological identity in T2D^97^ and electrophysiology of δ-cells was likewise reported to be disrupted in prediabetic mice^98^. However, in further analyses we decided to focus on β-cells due to their importance in diabetes development^94^.

### Diabetes-unique and cross-condition dysfunction in β-cells

**Extended Data Fig. 10:**
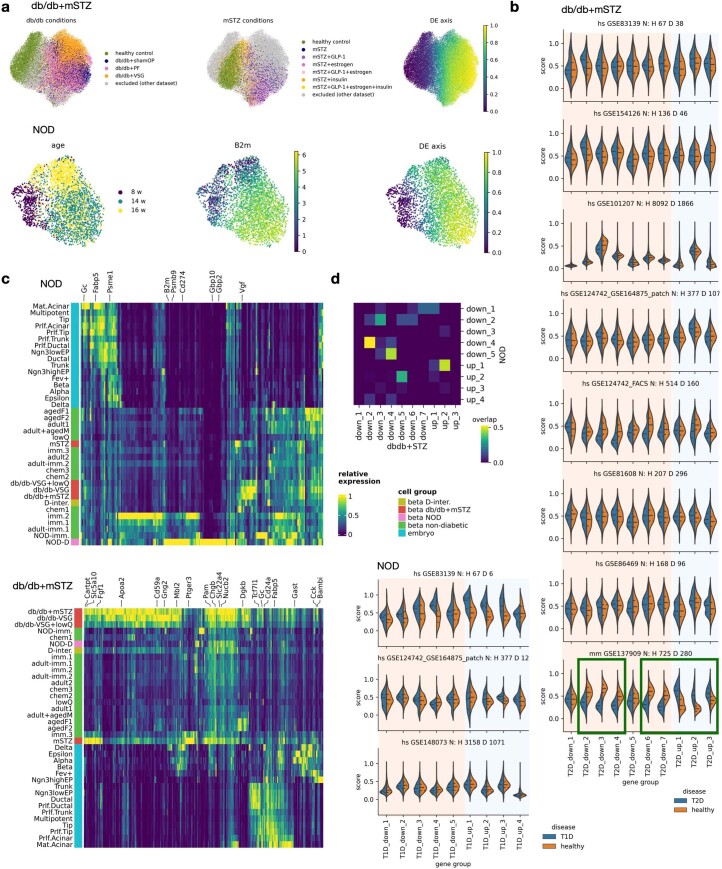
Diabetes-related molecular changes of β-cells show similarities and differences across dysfunctional states and translate to an external mouse dataset. (**a**) Design of DGE analysis showing original conditions in each used dataset (dataset 8-16wNOD for NOD DGE, datasets db/db and mSTZ for db/db+mSTZ DGE) and axis used for fitting the DGE model. For NOD we also show expression of a known T1D marker *B2m*. (**b**) Translation of diabetes model DEG groups (T1D NOD, T2D db/db+mSTZ) to external human and mouse datasets, indicated as normalized activity of gene groups in T1D or T2D (in mice STZ-treated) and healthy samples. Plot titles contain information on species (hs - human, mm - mouse), dataset and number of cells in healthy (H) and diabetic (D) groups. Encircled are gene groups that translate to the external mouse dataset. (**c**) Expression of genes upregulated in diabetic NOD or db/db+mSTZ cells shown across fine β-cell states and embryonic cell types as annotated in the original study. Cell color annotations are based on healthy and developmental conditions. (**d**) Overlap between NOD and db/db+mSTZ DEG groups as a ratio of the smaller group.

To find genes dysregulated in the T1D NOD model and T2D db/db and mSTZ model β-cells, a DGE analysis was performed for each model group. As cells within individual subjects can be heterogeneously dysfunctional, leading to reduced power in DGE analysis^78^, we leveraged MIA embedding to assign cells from healthy controls and disease models along a healthy–dysfunctional trajectory (Extended Data Fig. 10a and Supplementary Note 8). This is of special importance for NOD mice, as in the original study the authors observed incomplete penetrance^27^ dysfunctional β-cell phenotype^27^.

As the DGE analysis resulted in hundreds of DEGs that are expected to be heterogeneous in terms of their molecular function, we clustered them using their expression across all β-cells within MIA (sizes 12–349 genes; Fig. 6a and Supplementary Table 10). The groups are described in more detail in Supplementary Table 10 in terms of gene set enrichment, gene membership and cell states with high expression. In the text they are referred to as T1 groups for NOD and T2 groups for db/db + mSTZ.

First, we used the DEG groups to disentangle dysfunction patterns of interest from confounding effects. In the original NOD dataset paper by Thompson et al.^27^ the authors observed confounding of dysfunction progression and age differences between samples containing healthy (8 weeks) and dysfunctional cells (14 and 16 weeks), impairing the interpretation of diabetes-associated changes. Indeed, we also observed, among NOD downregulated genes, one group (T1-down1), which was highly expressed across multiple immature states (Fig. 6a) and contained genes associated with immaturity (*Pyy* and *Npy*)^99,100^ thus likely representing a confounding effect of age. Other gene groups did not seem to be associated with known batch effects.

With our DEG clustering approach, we disentangled two NOD-upregulated immune processes (groups T1-up2 and T1-up3) that showed differences in expression across β-cell states. Group T1-up3 was NOD diabetic cells (state 14–16wNOD) specific and more strongly enriched for antigen-processing genes (containing genes *B2m*, *Tap2* and major histocompatibility complex (MHC) II group members), whereas T1-up2 was, in addition to NOD diabetic cells, also highly expressed in immature cells (Fig. 6a) and more strongly enriched for innate immune response genes (containing genes *Stat1*, *Stat2*, *Gbp7* and immunoproteasome group members), potentially representing the regulation of β-cells by the immune system that is not restricted to diabetes^101^. Upregulation of both T1-up3 and T1-up2 in NOD is in accordance with the active involvement of β-cells in T1D-related immune response by means of antigen presentation and immune infiltration in the islets^27,102^, respectively. Furthermore, in the NOD diabetes model, we also observed upregulation of senescence-related genes (group T1-up4) that were shared with aged females (Fig. 6a). Indeed, senescence genes have been previously reported in association with NOD model dysfunction and aging individually^27,103^ and we here show their relationship.

As expected, in db/db + mSTZ cellular metabolism that is necessary for normal β-cell function^77^ was disrupted. A group of genes (T2-down3) was downregulated across all T2D model cell states and was higher across healthy cell states (Fig. 6a), with enrichment for insulin secretion and steroid metabolism. Additionally, we observed DEG groups supporting mSTZ subpopulations associated with immaturity or metabolic stress, which we observed above based on GP differences (Supplementary Note 9).

Multiple parallels can be drawn between NOD and db/db + mSTZ dysregulation. For example, NOD group T1-up1 also showed high expression in cell states from db/db and mSTZ datasets (Fig. 6a) and partially overlapped with db/db + mSTZ upregulated genes (Extended Data Fig. 10d), with the overlap containing multiple genes previously associated with diabetes (*Gc*, *Fabp5*, *Spp1* and *Vgf*)^104–107^. NOD and db/db + mSTZ also shared similarities in downregulated genes (T1-down4 and T2-down2; Extended Data Fig. 10d) that were, in turn, highly expressed in healthy mature cells (Fig. 6b). These groups contained multiple cross-species conserved β-cell genes (*Atf3*, *Btg2*, *Ddit3*, *Egr4*, *Fosb* and *Jun*)^108^, targets of β-cell expression program regulator CREB (*Per1*, *C2cd4b*, *Nr4a2*, *Fos* and *Dusp1*)^108,109^ and genes involved in management of metabolic stress involved in insulin production and secretion in non-diabetic β-cells (*Egr1*, *Hspa1b*, *Ddit3* and *Dnajb1*)^82,110^. This indicates that the β-cell phenotype is compromised across diabetes models. In contrast, some gene groups were conversely expressed in NOD and db/db + mSTZ analyses. For example, NOD group T1-down3, containing some genes involved in adaptive stress response (*Txnip* and *Herpud1*)^33,111^, was, in addition to healthy cells, also highly expressed in db/db and mSTZ model cells.

As it has been previously reported that diabetes results in the dedifferentiation of β-cells toward less-mature states in both mice and humans^22,23,30,112^ we compared the expression of upregulated genes across postnatal β-cell states and embryonic cell types, including endocrine cells and their progenitors. Among both the NOD and db/db + mSTZ upregulated genes we found genes that were strongly expressed in embryonic data or were specific to diabetes model cells (Extended Data Fig. 10c). This shows that changes in diabetes models involve both dedifferentiation as well as diabetes-model-specific responses.

To validate our findings, we further examined whether DEGs are translatable to other datasets. In the Feng dataset, which is not part of the atlas and contains STZ-treated samples^31^, most T2-groups had the expected expression direction in the STZ model cells (Extended Data Fig. 10b). However, two gene groups (T2-down1 and T2-down5) did not show different expression activity between diabetic-model and healthy Feng cells. For group T2-down5 the discrepancy could be explained by the gene group being most highly expressed in immature healthy cell states from MIA (Fig. 6a), which, as discussed above, are absent in the Feng dataset (Extended Data Fig. 7). In contrast, group T2-down1 had a relatively low expression difference between diabetic and healthy MIA cell states (Fig. 6a). For both gene groups, the observed expression patterns in MIA already indicate that they may not generalize to other datasets that have a somewhat different healthy and diseased cell state composition. The dissection of DEGs based on MIA β-cell states enabled us to explain why a subset of DEGs may not be translatable to other datasets, which is a common, usually unexplained, problem in scRNA-seq studies.

To support RNA-level DGE results (Supplementary Table 10) at the protein level, we selected relatively highly expressed DEGs and stained them with specific antibodies in islets from healthy and diabetes model (NOD and db/db) mice (Fig. 6d,e). First, we validated that islets contain expected healthy and dysfunctional β-cell states by profiling the protein expression of insulin, an established maturation marker Ucn3 (ref. ^9^) and a dedifferentiation marker Aldh1a3 (refs. ^113,114^) (Fig. 6d,e and Supplementary Note 10). We next profiled three new markers of the T2D model: Nucb2, which is involved in insulin secretion^115,116^ and whose mutations were reported to be associated with diabetes risk^117^, Fkbp11, an ER-located chaperone previously reported to be upregulated in certain mouse T2D models^118,119^ and Mt3, which was reported to be associated with β-cell death^120^. Protein and RNA levels of Nucb2 were upregulated in both NOD and db/db islets and Fkbp11 and Mt3 in the db/db islets. This validation supports the observations from our DGE analysis and proposes new dysfunction markers on both the RNA and protein level.

When comparing NOD and db/db + mSTZ genes to multiple human datasets we did not observe the expected DEG group activity differences between healthy and diabetic samples in a consistent manner (Extended Data Fig. 10b); however, certain diabetes hallmark genes translate across the species. For example, the *Dgkb* gene, whose ortholog is associated with human T2D^121^, was upregulated in our db/db + mSTZ analysis. Thus, future studies could use our diabetes DGE results to query for molecular changes shared with humans and thus assess whether pathways of interest could be further profiled with NOD, db/db or mSTZ models.

### A shared progression state in type 1 and 2 diabetes model β-cells

One of the key goals of diabetes research is to understand the transition from pre-diabetes to diabetes and back upon treatment to identify disease states where remission is still possible. To decipher the relationships between healthy and diseased states we calculated a partition-based graph abstraction (PAGA) on the fine β-cell states (Fig. 6f). The connection from the main healthy state (adult2, containing healthy adult cells across datasets) to the T1D model state (14–16wNOD) or the T2D model state (db/db + mSTZ) led in both cases via an intermediate state (D-inter.). Indeed, it has been suggested previously that both T1D and T2D may share some molecular stress patterns in β-cells, but diverge in the final outcome due to a persistent immune or metabolic challenge, respectively^27,122–124^; however, we did not find a report of a shared intermediate state in T1D and T2D models.

The intermediate state contained both stressed healthy and diabetic cells (Extended Data Fig. 5d and Supplementary Note 7), including cells from the Feng dataset mapped onto MIA (Fig. 4c); however, the sample with the largest cell proportion localizing in this state was the mSTZ diabetes model sample with regenerative anti-diabetic treatment^30^ (GLP-1 + estrogen + insulin; Extended Data Fig. 5d). This indicates that the intermediate state may be related to either treatment effects or diabetes progression and β-cell stress.

Molecular differences between the healthy and the intermediate state resembled those observed in the diabetic states (14–16wNOD, db/db + mSTZ; Extended Data Fig. 8c,e), as described in Supplementary Note 11. As the intermediate state may be related to both T1D and T2D models we profiled the expression of diabetes DEGs shared between T1D model and T2D model DGE analyses (described above). Most of these genes already exhibited expression differences between the healthy and the intermediate state and further changed from the intermediate to the diabetes model states (Fig. 6g and Supplementary Note 11). Notably, shared downregulated genes (89 genes) were strongly enriched for response to extracellular stimuli and transcription factor regulation of gene expression due to genes of activator protein-1 (AP-1) complex, which are involved in cell survival and death^125^. This indicates that regulatory mechanisms are disrupted between the healthy and intermediate states.

Our analysis suggests that the intermediate state presents a snapshot of the transition between healthy and dysfunctional cells in different diabetes models; however, it is unclear whether this is part of disease progression or a result of treatment and further investigations are required to clarify this state.

### Sex differences in β-cells involve diabetes-associated genes

Sex differences affect normal β-cell function and subsequent development of diabetes^126–129^. Therefore, we assessed sex differences across ages and their relationships to diabetes models. Two datasets from early postnatal (P16) and aged (2 years) mice with a mixture of male and female cells were used. In P16 mice we did not observe any DEGs, except for sex-linked Y-chromosome genes (*Ddx3y*, *Eif2s3y* and *Uty*), which were also used during data preprocessing for sex-annotation of cells. More DEGs were observed in aged mice (26 male and 116 female upregulated genes; Supplementary Table 11), which is also reflected in the clear separation of these cells into two distinct states (Fig. 5a). To further dissect the aged DEGs we clustered them based on expression across all β-cells of MIA, resulting in four female and four male groups (female1–4 and male1–4; Fig. 6c, Supplementary Fig. 3 and Supplementary Table 11).

Females are known to have higher insulin production and are less prone to develop T2D^18,130^. Indeed, we observed some DEG groups explaining these phenotypes. Group male4, which was highly expressed in T2D model state (Fig. 6c), contained multiple genes related to dedifferentiation, immaturity and other endocrine cell types^49,113,131–133^ (Supplementary Table 11). In contrast, the female1 group, which was likewise expressed in T2D model state (Fig. 6c), contained multiple genes previously reported to be upregulated in pregnancy^23,134^ (Supplementary Table 11) as well as genes related to insulin secretion (*Chgb*)^135^ and stress response (*Mapk4* and *Gpx3*)^136,137^. Furthermore, a group expressed specifically in aged female cells (female4, 78 genes; Fig. 6c), contained some genes involved in insulin regulation^138–140^ and glucose metabolism^141,142^ (Supplementary Table 11). Altogether, this indicates that female β-cells are more inclined to diabetes-associated compensation and male β-cells to loss of identity.

## Discussion

Here we present the MIA, a high-quality integrated atlas, that compiles multiple developmental stages and disease conditions from 56 samples with transcriptomics readouts of over 300,000 cells. The exploration of MIA provides new insights into islet biology and diabetes research that could not have been obtained from individual datasets. Our key discoveries are the description of the β-cell landscape from diverse datasets, the proposition that mSTZ diabetes model molecularly resembles T2D rather than T1D and the identification of molecular pathways involved in different types of β-cell dysfunction. While this paper is focused on β-cells, we also showcased that MIA can be used for studying other cell types, presenting an opportunity for future studies.

We used MIA to comprehensively describe the β-cell landscape across datasets and conditions. We identified molecular variation conserved across healthy adult β-cells. This included pathways of immaturity and aging as well as pathways potentially involved in cycling between insulin production and metabolic stress, followed by regeneration. We further proposed the use of GPs to identify and characterize molecularly distinct cell states in the β-cell landscape. This led to the identification of an intermediate β-cell state between healthy controls and different diabetes models that may be involved in diabetes progression or treatment-induced remission. We also observed two distinct populations within the mSTZ model differing in immaturity and compensatory phenotype, which may be of relevance when using the STZ model in future diabetes studies. Notably, when comparing different diabetes models, we observed that β-cells in the STZ model exhibited a gene expression profile akin to the db/db model and not the NOD model. This was again reflected in comparison to human data, where mSTZ β-cells showed upregulation of T2D-related metabolic stress pathways while lacking upregulation of T1D-related immune pathways.

For future studies, MIA enables automatic cell type and state transfer as well as cross-study and cross-condition comparison by embedding cells into a shared reference space. We have demonstrated this with the Feng dataset, which is not part of MIA, resulting in the expected mapping of healthy control and STZ diabetes model β-cells to the corresponding MIA regions. This also showed that the immature populations present in MIA and the Feng dataset differ, indicating that the reason for them not sharing markers is likely of biological nature, attributed to different cell states. Our vision is that future studies can similarly map their datasets on top of MIA and publicly provide the generated embeddings to further extend the conditions compiled in MIA. As an example, we showed this for a young (P3) sample from the Feng dataset, for which we do not have a matched developmental stage in MIA, with its embedding filling the gap between our embryonic and older postnatal samples.

The heterogeneity compiled within MIA also enables contextualization at the gene level. For example, known β-cell maturity and dysfunction markers are more heterogeneous than expected, showing distinct expression subgroups across β-cells states of MIA. Similarly, researchers could use the interactive cellxgene^143^ instance of MIA to analyze the expression of their genes of interest across cell types and diverse biological conditions within MIA.

Our next aim was to describe which pathways are involved in different β-cell dysfunction phenotypes. Therefore, we used MIA to group DEGs and contextualize them based on expression across other conditions. For diabetes-model DEGs this approach revealed phenotype specific as well as shared molecular changes across diabetes models, aging and immaturity. Grouping of DEGs also identified distinct dysfunction-associated changes across sexes, explaining lower susceptibility of females for diabetes due to upregulation of compensatory rather than loss of identity pathways that were observed in males. In the future, the dissection of dysfunction patterns based on multiple phenotypes may provide valuable insights for personalized medicine, which is based on knowledge about different disease-associated molecular patterns. It may also be useful for drug repurposing, which relies on pathways shared across diseases^144–146^. For example, it was previously shown that removing senescent cell populations in NOD mice and models of aging improves the overall regulation of glucose levels^27,103^. Indeed, in our analysis, we observed upregulation of senescence-associated genes in both aged and T1D model cells.

We show that our results are reproducible in independent mouse transcriptomic data and in immunohistochemistry, proposing new markers of T2D model-associated dysfunction (Nucb2, Fkbp11 and Mt3). Comparison to human datasets revealed some similarities to mice; however, new methods will be required to improve cross-species comparison and translation.

In conclusion, MIA provides a useful tool for islet biology and diabetes research. It is available as a curated resource in formats that enable interactive exploration via cellxgene and computational analyses (https://github.com/theislab/mouse_cross-condition_pancreatic_islet_atlas), including access to the cellxgene curated dataset via Sfaira^147^. Our discoveries in β-cell biology showcase how MIA can be used both as a reference of cell states as well as for further querying of gene expression across conditions.

## Methods

Animal studies were conducted with adherence to relevant ethical guidelines for the use of animals in research in agreement with German animal welfare legislation with the approved guidelines of the Society of Laboratory Animals and the Federation of Laboratory Animal Science Associations. The study was approved by the Helmholtz Munich Animal Welfare Body and by the Government of Upper Bavaria.

### Generation of new mouse samples included in the atlas

Mice were housed in groups of two to four animals and maintained at 23 ± 1 °C and 45–65% humidity on a 12-h dark–light cycle with ad libitum access to diet (irradiated standard diet for rodents, Altromin 1314, Altromin Spezialfutter) and water.

Islets of Langerhans have been isolated using a standard protocol^148,149^. The aged dataset was generated from islets of Langerhans isolated from the Fltp lineage-tracing mouse model (Fltp iCre mTmG)^150^ in mice older than 2 years. Two male and two female mice were pooled together after islet isolation and before FACS. The sorting was used to separate cells into Fltp-negative (tomato-positive), Fltp-lineage-positive (GFP positive) and Fltp-transient (double-positive) populations (Supplementary Fig. 4), using FACSDiva (v.6.1.3) and FlowJo (v.10.8.1) software. Separate libraries were generated for each sorted population after pooling across sexes. For the 4m dataset, we used the Fltp reporter mouse line Fltp^ZV^ (ref. ^151^). The pancreas head and tail were anatomically separated before islet isolation. Islets from six Fltp^ZV/+^ male mice were pooled. Subsequently, Fltp Venus reporter-positive and negative cells were sorted (Supplementary Fig. 4), thus generating four libraries. The metadata of all samples are shown in Supplementary Table 1.

Libraries of single cells were produced using the Chromium Single-Cell 3′ library and 10x Genomics gel bead kit v.3.1 (PN 1000121) in the aged dataset and with v.2 (PN 120237) in the 4m dataset. Briefly, 10,000 cells were loaded per channel of a 10x chip to produce gel bead-in-emulsions (GEMs). Then the samples underwent reverse transcription to barcoded RNA, followed by cleanup, complementary DNA amplification, enzymatic fragmentation, 5′ adaptor and sample index attachment. The samples of the aged dataset were sequenced using a NovaSeq6000 (Illumina) with 100-bp paired-end sequencing and the samples of 4m dataset were sequenced using a HiSeq4000 (Illumina) with 150-bp paired-end sequencing of read 2.

### Datasets included in the atlas

We used nine mouse pancreatic islet scRNA-seq datasets previously generated with 10x Genomics Chromium technology. Data availability is described in Table 1. Public data were obtained from the GEO in July 2020 by comprehensively searching for mouse pancreatic islet scRNA-seq datasets. From the collected datasets we excluded datasets that would not be applicable for analysis of β-cell heterogeneity, such as cancer and reprogramming datasets as well as datasets with low endocrine cell counts, including embryonic datasets, with the exception of an in-house embryonic dataset. We also excluded datasets that were not generated with Chromium (namely Smart-seq2) as most of them had low cell counts and could lead to strong cross-technology batch effects due to differences in sensitivity and bias in the type of captured genes^152^. Furthermore, some of the integration methods are not designed for full-length reads, such as Smart-seq2 (ref. ^41^). Altogether, using additional sequencing technologies would make the integration more challenging.

All computational analyses of scRNA-seq data were performed with Scanpy (v.1.6–1.8.1)^153^, except where noted elsewhere.

### Datasets for atlas validation

For validation we collected public mouse and human scRNA-seq datasets (Table 2 and Supplementary Table 12) and downloaded their expression count matrices and metadata from GEO and paper supplements. If raw counts were available, re-normalization was performed with the Scanpy normalize_total function, otherwise, the available pre-normalized data were used. For downstream analyses, log(expr + 1)-transformed normalized expression was used. We manually unified cell type annotation from original studies to a shared set of cell-type names by renaming existing labels. No further preprocessing was performed on these datasets. These datasets were not included in the atlas and were always analyzed individually. In the text, we refer to the GSE137909 dataset as the Feng dataset. Where necessary, we mapped genes across species based on ortholog information from BioMart^154^ (Ensembl Genes v.103).

### Preprocessing of datasets for atlas building

Gene expression counts were calculated based on genome versions described in Table 1 with 10x Genomics CellRanger (v.2.2.1–v.3.1.0)^155^. Each dataset was separately preprocessed with the below-described steps, except when we note that a processing step was performed per sample, and filtering thresholds were determined on a per-dataset level.

### Ambient gene identification

To reduce the effect of ambient expression on embedding calculation we removed the most prominent ambient genes, which were identified as described here. We selected likely empty droplets that contained only ambient RNA based on having fewer than 100 counts. Gene proportions within empty droplets were computed on raw counts per sample, representing gene proportions within the ambient RNA. Genes with the highest ambient proportion were selected with a dataset-specific ambient proportion threshold, selecting genes as the union across samples, generating a set of approximately 20 genes per dataset. Owing to the proportional nature of expression measurements a relatively high ambient proportion of some genes leads to lower proportions in other ambient genes. Thus, we reduced the ambient threshold when some genes had a relatively high ambient proportion to also capture fewer ambient genes that are nevertheless known to strongly affect ambient profiles, such as endocrine hormone genes. Additionally, a larger set of approximately 100 genes was generated with a more permissive threshold that aimed to include top ambient genes so that selecting more genes would no longer evidently increase the captured cumulative ambient proportion given by the sum of the per-gene ambient proportions.

### Dataset quality control

Empty droplet score was computed per sample with DropletUtils (v.1.10.3)^156^ emptyDrops function using LogProb output for downstream visual quality control assessment purposes. Cell-containing droplets as determined by the CellRanger pipeline were used in downstream analyses. Cell filtering was performed based on guidelines published previously^157^, excluding cells with a low number of expressed genes, low total counts or high mitochondrial proportion and outliers with a very high number of total counts or expressed genes. Genes expressed in a very small number of cells and top ambient genes were excluded for the purpose of annotation and integration. Doublets were filtered out with Scrublet (v.0.2.1)^158^ scores computed per sample using a manually set threshold to separate the scores into cross-cell type doublet and potential non-doublet populations as proposed in the tutorial^158^, while ensuring that selected doublet cells mainly mapped into discrete cluster locations on the Uniform Manifold Approximation and Projection (UMAP) embedding. The choice of the threshold was set permissively, as indicated by the presence of some residual doublet populations in the final atlas version.

### Dataset-wise cell annotation

To perform cell annotation within individual datasets normalization was performed per dataset with scran (v.1.16.0–1.18.7) pooled size factors^159,160^, data were log(expr + 1)-transformed and 2,000 highly variable genes (HVGs) were selected with Scanpy using the cell_ranger selection flavor and samples as batches. The cell cycle stage of each cell was annotated using the Cyclone method^161^ as implemented in scran. For datasets without per-cell sex information, the sex was annotated based on Y-chromosome located HVGs with high expression. We assigned cells into insulin, glucagon, somatostatin and pancreatic polypeptide high or low groups per-sample based on scores from the Scanpy score_genes function. Cell types were annotated in the following datasets: P16, 4m, aged, mSTZ (healthy sample), db/db (healthy samples), based on known pancreatic cell type markers followed by recursive subclustering until homogenous clusters were reached. Rare cell types that did not form a separate cluster were annotated based on per-cell marker scores (for example, ε-cells in the P16 dataset). Here and in the below re-annotation of the integrated data we relied on the following cell type markers across multiple datasets, although on the per-dataset level, we also used other markers, expressed in cell subpopulations present in only some of the datasets. The marker list is acinar: *Cpa1*, *Prss2*; α: *Gcg*; β: *Ins1*, *Ins2*; δ: *Sst*; ductal: *Krt19*, *Muc1*, *Sox9*; endothelial: *Pecam1*, *Plvap*; ε: *Ghrl*; γ: *Ppy*; immune: *Cd52*, *Lyz2*, *Ptprc*; stellate-activated: *Col1a2*, *Bicc1*, *Pdgfra*; stellate-quiescent: *Ndufa4l2*, *Acta2*, *Cspg4*, *Rgs5*; and Schwann: *Cryab*, *Plp1*, *Sox10*. Expected multiplet rates were computed and together with Scrublet scores used to determine which annotated multiplet cell types present true cells or residual multiplets. We annotated β-cell states based on the expression of known β-cell heterogeneity markers.

### Batch-wise preprocessing for integration

We tested different methods for ambient expression correction: CellBender (v.0.2.0)^162^, SoupX (v.1.5.0)^163^ and DecontX (from celda v.1.5)^164^. We did not use CellBender preprocessed data further as we observed non-homogeneous correction within clusters, namely some genes known to be cell type-specific, such as β-cell-specific *Ins1* and *Ins2*, were removed partially and at different levels across cells within other cell types. For other methods, different ambient correction strengths were used and one or more were selected for integration per method. Non-ambient-corrected data were also used. Top ambient genes were excluded, also in ambient corrected datasets (using the smaller ambient gene set). The ambient correction method selected for final integration is described in the ‘Integration selection’ section. Genes previously marked as too lowly expressed on a per-dataset level were also removed. To enable integration with samples as batches and future mapping of new samples onto the reference the data was per-sample scran normalized and transformed with log(expr + 1). The batch-wise re-normalization was performed as scran size factors may not be comparable across multiple runs due to size factors being relative within a dataset^160^. These additional batch differences can thus be learned to be corrected by the integration model. By performing batch-wise normalization (here, batch is a sample) we ensure that the integration model can account for this effect when removing batch effects. For scVI integration non-normalized data were used. Expression matrices of all samples were merged, retaining the intersection of genes. The 2,000 HVGs obtained with the scIB (developmental version, last updated on 17 January 2022)^41^ hvg_batch function was used.

### Integration selection

For integration we used scVI v.0.7.0a5 (ref. ^40^) with hyperopt hyperparameter optimization and scArches v.0.1.5 (ref. ^42^) with manual parameter optimization. First, we performed integration on the annotated data only to select scVI parameters with hyperopt (number of network layers and their size, number of latent dimensions, reconstruction loss, dropout rate, learning rate, gene dispersion and number of epochs) and scArches parameters based on visual evaluation (different HVG selection, integration strength regulated by the weight between reconstruction and Kullback–Leibler divergence loss, number of network layers and reconstruction metrics), to ensure that selected parameters lead to a reasonable integration. Afterward, integration was performed on all data. Different integration methods and preprocessing combinations were evaluated with scIB metrics. We added a new biological conservation metric named Moran’s I conservation, which does not require cell-type annotation. For biological conservation evaluation we excluded unannotated and multiplet cells, except for Moran’s I, which could be run on all cells. As annotation was available only for a subset of cells the batch correction metrics were run both on all data, using clusters instead of cell-type labels and on the annotated data subset. We also performed evaluation on β-cells only, using β-cell states as cell labels, with different integration strengths. Top selected integrations were run multiple times to better distinguish between random initialization and true performance variation. The best method (removed top ambient genes and scArches-cVAE) was selected based on summarized biological conservation and batch correction scores, as described in scIB, with a special focus on β-cell state conservation.

We also tested β-cell-specific integration, using β-cells defined based on an integrated annotation (see below) with the same integration settings as for the whole atlas, but with multiple different integration strengths in scArches-cVAE. Batch correction evaluation was run on all cells, using clusters instead of cell type labels and biological preservation evaluation on cells that had state annotation. The results were compared to metrics computed on the same set of cells from the whole atlas integration.

For comparison, we also show unintegrated embedding, which was computed using the same set of genes as the final atlas integration. We normalized expression using the Scanpy normalize_total function as scran normalization performed on individual samples, as used for integration, leads to lower comparability of normalization factors across samples. Data were log(expr + 1)-transformed and scaled, followed by principal-component analysis (PCA)-embedding computation that was used as the basis for UMAP.

### Integration evaluation with Moran’s I conservation

We proposed a new biological conservation metric for comparison across integration runs without the need for cell type annotation that determines how strongly genes are variable across the integrated embedding. Namely, if embedding captures biological variation at a finer scale, for example, within cell types, then the expression variation of genes that are potential determinants of cell state differences (for example, HVGs) should be non-random across the embedding. The method first computes HVGs ($g$, 1,000 genes) on the expression data with Scanpy highly_variable_genes function using cell_ranger flavor and batch_key parameters. Moran’s I for these HVGs is then computed on the integrated embedding ${(i)}$ with Scanpy morans_i function. This function uses information about each cell’s *k*-nearest neighbors graph computed with Scanpy neighbors function on the integrated embedding with Euclidean distance metric. The final score is computed as the mean of per-gene scores. This score is rescaled to fall within range [0,1], matching other scIB scores. This can be formulated as:

$${{\mathrm{score}}}=\frac{\frac{1}{g}\mathop{\sum }\limits_{1}^{g}({i}_{g})+1}{2}$$

### The final annotation of the integrated atlas

We defined cell types on the integrated atlas by consecutive Leiden^165^ subclustering with Scanpy, namely by manually selecting clusters to be subclustered as needed to separate cell types, relying on information about previously annotated cells, hormone expression high/low assignment and quality metrics. Namely, empty droplets were identified based on low expression and high empty droplet probability and doublet clusters based on higher doublet scores and expression of markers of multiple cell types. We compared the re-annotation to the annotation from original publications, for which we manually unified cell type labels by renaming the labels to a shared set of names.

As scran normalization performed per-sample is not comparable across samples (described above) scran size factors were recalculated on the integrated cell clusters and the atlas was jointly re-normalized. In downstream analyses, we used this normalized data, except for the methods that required raw counts.

To disentangle biologically relevant differentially active genes from genes whose expression is likely a result of ambient expression differences in the downstream analyses, we defined genes that may be predominately ambiently expressed in a given cell type. Top ambient genes likely not coming from β-cells were defined as follows. For each sample, genes with high expression in empty droplets, containing fewer than 100 counts, were selected with a single threshold across all samples and the genes were pooled across samples. These ambient genes were clustered based on expression across integrated cell clusters. Ambient gene clusters were assigned to non-β-cell originating ambient genes if they had relatively low expression across all β-cell clusters compared to cell clusters coming from other cell types. Besides making the set of likely non-β-cell ambient genes, we used during interpretation a per-gene metric that can indicate ambient gene origin, namely relative gene expression in a cell type compared to other cell types, with higher scoring genes being less likely ambient. As this metric was used for postnatal endocrine analyses the embryonic clusters were excluded as they are not expected to contribute to ambience in postnatal samples. The atlas subset was then subclustered using Leiden clustering with resolution of 2. Mean expression in cell clusters was maxabs-scaled across clusters, representing relative expression in each cluster. To determine the relative expression of a gene in a cell type we used the highest relative expression obtained across all cell clusters containing predominantly that cell type.

In all further analyses where we needed to reduce the number of cells due to computational constraints we prepared pseudobulk data (here, termed ‘fine pseudobulk’) by Leiden clustering with high resolution (such as resolution of 20) to create tens or hundreds of clusters (depending on data size) that should capture the majority of heterogeneity within the data. This is akin to recently proposed methods that aim at creating so-called ‘metacells’ that group together cells without biological differences^166,167^. Pseudobulk expression was computed as the mean of log(expr + 1)-transformed normalized expression within each cluster. For DGE analysis on pseudobulk (here termed ‘metadata-based pseudobulk’) we grouped cells based on their metadata, such as sample and cell type, as before suggested for single cell DGE analysis^168^. Here, normalized counts were summed across cells and log(expr + 1)-transformation was not applied.

### Identification of endocrine cell type markers

For the identification of endocrine cell type markers one-versus-one DGE analyses were performed with edgeR (v.3.32.1)^169^. For the postnatal markers metadata-based pseudobulks of postnatal datasets per cell type, sample and sex were created. We excluded embryonic, doublet and endocrine proliferative cell types. The former cell type was excluded as a minute number of postnatal cells mapped to the embryonic clusters (Extended Data Fig. 1). The latter two cell type groups were excluded as they share gene expression with matched non-doublet and non-proliferative cell types, which would prevent the identification of these genes as DGE markers. Lowly expressed genes were removed with edgeR and a single DGE test was fitted, using edgeR general linear model (GLM) with robust dispersion, with sample and sex as covariates and two-sided likelihood-ratio significance testing. To obtain one-versus-rest upregulated genes for each endocrine cell type the factors across cell types were compared. Marker genes were selected based on a false discovery rate (FDR) <0.05 and log fold change (FC) >1.5 against all other cell types. In the supplementary tables we reported the maximal adjusted *P* values across compared cell types and for logFC we reported 0 if logFC across comparisons had both negative and positive values and otherwise signed minimal logFC based on absolute value sorting. For embryonic markers, the embryonic dataset with cell type annotation from the original study^60^ was used. The Fev^+^ cluster was excluded as it contained precursors of individual endocrine cell types with similar expressions as in the descendant cell types, which would prevent the identification of markers. Metadata-based pseudobulks were created per cell type and sample, whereas sex was not used as a covariate, as at this age strong sex differences were not expected. Endocrine cell-type markers were identified as for the postnatal datasets. In the postnatal dataset, we used 52 samples and in the embryonic dataset we used 4 samples, with some cell types being represented in fewer samples and some samples containing data pooled across multiple animals.

### Comparison of embryonic and postnatal endocrine cells

We grouped α-, β- and δ-cells into three groups per cell type: embryo (cells that were annotated as a certain endocrine cell type in the original embryo study and mapped into the embryo endocrine atlas cluster); embryo postnatal-like (cells from the embryo dataset that mapped into one of the postnatal endocrine atlas clusters); and postnatal (cells from postnatal datasets that mapped into one of the postnatal endocrine atlas clusters). For embryo and embryo postnatal-like cell types, we computed what proportion of embryonic cells per sample-specific age group they represent.

### Reference mapping of the external mouse dataset

The Feng dataset (query) was re-normalized per-sample with scran and log(expr + 1)-transformation to match atlas (reference) datasets preprocessing. The reference scArches model was used to compute the query embedding, using samples as batches. For query β-cell mapping analysis the cell type annotations from the original study^31^ were used. A joint UMAP embedding of query and reference β-cells was computed, as well as a UMAP with added reference embryonic β-cells, using β-cells from the original study annotation^60^ that mapped into the atlas embryo endocrine cluster, and reference proliferative β-cells, defined as endocrine proliferative cells that were previously annotated as highly expressing insulin, but not other hormones. Query β-cell states were predicted based on atlas coarse β-cell states with the addition of embryonic and proliferative β-cell groups. For cell type transfer a weighted *k*-NN classifier adapted from scArches manuscript^42^ was used with an uncertainty threshold of 0.75.

### Comparison of diabetes models to human T1D and T2D

To obtain T1D and T2D gene sets conserved across human datasets the T1D or T2D cells were compared against cells from non-diabetic samples in each human dataset (the number of samples in each group varied across datasets; Supplementary Table 12 shows sample group sizes). Only genes expressed in at least 10% of diabetic or healthy cells per dataset were used. Genes with an FDR <0.25 and logFC >0.5 in at least half of the datasets based on the Scanpy rank_genes_groups *t*-test function (two-sided Welch’s test on cell level) were selected.

Gene set enrichment was computed with hypeR (v.1.6.0)^170^ at the FDR threshold of 0.25 using Gene Ontology (GO), KEGG and Reactome gene sets from MSigDB (v.7.4.1). Before enrichment, each gene set was subsetted to genes present in the background that consisted of all genes used for the analysis (here, genes tested for DGE) and gene sets containing less than five or more than 500 genes were removed. From enriched gene sets with shared genes, we manually selected representative gene sets to be highlighted in the text.

Mouse diabetes model β-cells were scored for both the newly defined and literature-based gene sets with Scanpy score_genes function on each dataset. Comparisons were performed between the following groups: in the 8–16wNOD dataset the 8-week (healthy) versus 14- and 16-week samples (diabetic); in the mSTZ dataset control (healthy) versus the mSTZ-treated sample (diabetic) and in the db/db dataset control (healthy) versus db/db sham-operated samples (diabetic). Gene set score distributions in healthy and diabetic groups within each dataset (sample numbers for healthy mSTZ = 1, db/db = 2, 8–16wNOD = 3; and diabetic mSTZ = 1, db/db = 2, 8–16wNOD = 6; some samples contained pooled animals) were compared using a two-sided Mann–Whitney *U*-test on cell level and a natural-logarithm based logFC was computed between distribution medians.

### Coarse β-cell states and their markers

Clusters were computed with the Scanpy Leiden function and were thereafter added descriptive annotation based on sample ratios across clusters, relying on sample metadata, quality scores and relationships between clusters determined with PAGA. Initial clustering was performed with a relatively high resolution so that we could later merge clusters that we could not interpret as separate based on the criteria described above while ensuring that we did not miss any unique clusters.

Cluster-specific markers conserved across datasets were computed as follows. Data were subsetted to exclude low-quality clusters and the embryo dataset as it contained too few β-cells (fewer than 20 per sample across all β-cell clusters). Cell groups used for DGE were defined as a combination of cluster and dataset, using for each cluster only datasets with a high proportion of cells in that cluster in at least one sample. For each dataset-cluster group DGE analysis was performed with the Scanpy rank_genes_groups *t*-test function against all other cell groups, except the ones from the same cluster, excluding genes that were lowly expressed in both clusters before DGE analysis. The number of samples per group varied across cell states, with the total number of considered samples before grouping being 52, with some samples containing pooled animals. As markers, we selected genes that were significantly upregulated (FDR < 0.1 and logFC > 0) in all datasets across all other cell groups and for plotting genes were prioritized based on the highest minimal logFC across all comparisons. Genes were further filtered to select likely non-ambient genes by keeping only genes with relatively high expression in β-cells (>0.7). Hemoglobin genes were also removed as they were not caught by the relative expression filter as erythrocytes are absent from data, but the transcripts are still present in the ambient RNA.

Markers of adult, immature and T2D model states were visually validated on the external mouse dataset. The healthy β-cells were grouped by age and the STZ-treated cell groups were based on the administration of insulin.

Translation of markers to the human data was tested based on all collected human datasets with per-dataset one-versus-rest one-sided *t*-tests on cell level and *P* value significance threshold of 0.05. We also report log_2_-based logFC between group means. The following cell groups were defined: T1D or T2D groups contained all cells annotated as T1D or T2D and were used to test both known T1D or T2D markers as well as our NOD or db/db + mSTZ markers, respectively and for other marker groups only healthy donor cells were used, with the adult set used to test our adult mouse cluster and contained ages of 19–64 years, mature set used to test known maturity markers and contained ages of 19 years or more, aged male or female sets contained ages of 65 years or more and immature set ages of 18 years or less. Age groups were defined based on OLS HsapDv human life cycle stages definitions^171^. The number of samples varied across groups and datasets (Supplementary Table 12 provides more details).

### Gene programs in β-cells

To define GPs we first identified genes variable across embedding and then clustered them based on coexpression (Fig. 5d), as described below. To identify variable genes low-quality coarse β-cell clusters were excluded before the analysis as they could lead to high spatial autocorrelation scores of genes associated with data quality. Lowly expressed genes and the non-β-cell ambient gene set were removed. Moran’s I was used to assess the autocorrelation of expression across the integrated embedding (all 15 dimensions). We observed a bias of genes expressed in fewer cells toward lower Moran’s I, which would lead to lowly expressed genes unjustly being less often selected as variable based on Moran’s I threshold. To account for this bias, we regressed out the effect of the number of cells expressing the gene on Moran’s I. For this regression we used genes likely not to be truly variable across the embedding, as explained below, to estimate the base-level effect of expression sparsity across cells on Moran’s I. Genes likely not to be truly variable were selected as follows: most highly expressed genes (*N* cells ≥ 40,000 from a total of 99,361 cells) were excluded as they were deviating from the trend toward higher Moran’s I values, which was likely due to their importance in β-cell function and thus higher variability across the β-cell embedding. The remaining genes were binned (*N* bins = 20) based on the number of cells in which they were expressed and the five genes with the lowest Moran’s I from each bin were selected for regression, representing the base-level (likely not biologically relevant variable) Moran’s I at certain expression strength. The regression was fitted on the selected genes and then the corrected Moran’s I score was computed as the residuals from regression for all genes for which the uncorrected Moran’s I score was initially computed. Finally, GPs were defined by selecting genes with the highest corrected Moran’s I and clustering them using fine pseudobulk cell clusters as features with hierarchical clustering and visually determined cutting threshold based on a heat map of gene expression across pseudobulks. Gene set enrichment of GPs was computed as for the human T1D and T2D conserved genes. We supplemented GP gene set enrichment interpretation with marker-based domain knowledge to support β-cell-specific functional annotation, which is not fully encompassed by the more generic gene sets available in KEGG, GO and Reactome.

The ratio of variance explained by GPs per dataset was computed based on principal component (PC) regression. For each dataset, lowly expressed genes were removed and 50 PCs were computed based on HVGs. Cells were scored for GP activities with the Scanpy score_genes function (excluding genes missing from each dataset from GPs) to analyze how well GP scores of all or individual GPs explain each PC based on regression *R*^2^ (coefficient of determination). The total variance explained was computed as a sum of *R*^2^ across PCs weighted by the ratio of variance explained by each corresponding PC. For comparison, the same procedure was used to evaluate variance explained by random gene groups of the same size as the GPs, repeating the procedure ten times to estimate the random distribution. For the analysis of explained variance in healthy mouse and human samples, only samples with at least 100 β-cells were used and the explained variance was computed as described above, repeating the calculation for random gene groups 100 times. The significance of the explained variance by GPs was computed as a one-sided empirical *P* value compared to the distribution for the matched random gene group.

### Fine β-cell states

Each cell was scored for each GP with the Scanpy score_genes function followed by averaging within the fine pseudobulk clusters to speed up further analysis. The GP scores were used as features to cluster pseudobulk clusters into β-cell state clusters using hierarchical clustering followed by visual selection of the cutting threshold based on GP activity purity within clusters and unique pattern of GPs across clusters. Each cell was assigned to the cluster of its pseudobulk group. The clusters were named based on the metadata of the samples with a large proportion of cells within the cluster. The resulting β-cell state clusters were used to obtain a pruned PAGA graph, selecting a pruning threshold that separated between high and low connectivities.

We analyzed GP-based molecular differences for individual datasets between healthy and diseased states (adult2 versus db/db + mSTZ (for datasets db/db and mSTZ) and versus NOD-D (for dataset 8–16wNOD)) and two diseased states (db/db + mSTZ and mSTZ for dataset mSTZ). All β-cells were scored for GP activity with the Scanpy score_genes function and individual scores were normalized across cells to [0,1] with winsorizing by removing the highest and lowest 20 cells for setting the scaling range. The per-dataset differences between means of the normalized scores within clusters were then used for cluster comparison.

We manually extracted known markers of β-cell heterogeneity from the literature. For plotting across fine β-cell states we excluded markers expressed in less than 1% of β-cells and plotted mean expression per cell state. A heat map was created with ComplexHeatmap (v.2.11.1)^172,173^.

### Conserved β-cell heterogeneity in healthy samples

Low-quality coarse β-cell clusters were excluded as they could lead to high spatial autocorrelation scores of genes associated with data quality. Control samples from the chem dataset were not used as they showed lower integration of β-cells, indicating potential strong batch effects, which could negatively affect the identification of variable gene groups conserved in healthy β-cells. Thus, healthy adult samples from db/db, mSTZ and 4m datasets were used. For each sample, lowly expressed genes were removed and a neighborhood graph was computed on per-sample PC embedding for Moran’s I computation, as described in the ‘Gene programs in β-cells’ section. Here, we adjusted the threshold for removing genes expressed in many cells from Moran’s I score correction regression to expression in at least 30% of cells. Genes with high Moran’s I in all samples were selected. To ensure that gene clusters are conserved across samples the genes were clustered based on the highest distance on per-sample fine pseudobulks using hierarchical clustering. The cutting threshold was visually determined based on a heat map of gene expression across per-sample pseudobulk. Gene group scores were compared to the expression of known β-cell functional and phenotypic markers extracted from the literature, with marker correlations computed on per-sample pseudobulks and summarized as a mean of per-dataset means across per-sample scores. Gene set enrichment was computed as for β-cell GPs.

To find the cells with the highest expression of each gene group we used Scanpy score_genes function on individual healthy adult samples, followed by selection of 50 cells with the highest score. As the Feng dataset had a low number of healthy adult β-cells we performed scoring on all control samples together and selected only the top 20 cells per gene group.

### Differential expression in T1D model and T2D model β-cells

We performed DGE analysis on all samples from 8–16wNOD (*n* = 9) and from db/db and mSTZ (*n* = 15, samples contained pooled animals) datasets, excluding low-quality coarse β-cell clusters. A continuous disease process (Extended Data Fig. 10a) was computed with MELD (v.1.0.0)^174^ on the integrated embedding as healthy sample densities normalized over healthy and diseased densities, using for healthy and diseased the same set of samples as in the diabetes model comparison to human diabetes-associated gene sets. In the db/db + mSTZ analysis, the final MELD healthy and diseased scores were computed as a mean over datasets-specific scores. We observe that the resulting process corresponds to the gradient from the healthiest (highest healthy sample cell density within a region) to the most diabetically stressed cells (highest diabetes model sample cell density within a region), with the process value of individual cells being determined based on cell embedding location rather than just sample membership. Genes expressed in less than 5% of healthy or diabetic sample cells were removed. To assess linear change in gene expression along the disease process we used diffxpy (v.0.7.4)^175^ two-sided Wald test that fits a negative binomial model to raw counts across cells using expression normalization size factors as exposure. Dataset information was used as a covariate in the db/db + mSTZ analysis. The DEGs were selected based on FDR < 0.05, logFC (binary logarithm of the relevant model coefficient representing linear change) >1 and relative expression in β-cells >0.2, to keep only genes that are less likely ambient, as described above. For comparison to the embryonic data the [0,1]-normalized expression of upregulated genes was plotted across fine β-cell states and embryonic clusters as annotated in the original study.

For both DGE analyses the up- and downregulated genes were separately hierarchically clustered on the whole β-cell fine pseudbulk data. Cutting thresholds were selected visually based on heat maps portraying gene expression grouped across fine pseudobulks. All β-cells were scored for DEG groups with the Scanpy score_genes function and the scores were averaged within β-cell clusters. Gene set enrichment was computed as described for human T1D and T2D genes. Gene membership across groups was compared as the relative overlap normalized by the size of the smaller group.

The DEGs in NOD and db/db + mSTZ were compared to three human datasets with T1D samples and one mouse and seven human datasets with T2D samples, respectively. We scored cells for each DEG group activity with the Scanpy score_genes function, followed by [0,1] normalization across cells and separately plotted cells from healthy and diabetic samples.

For analysis of the DGE patterns in relationship to the D-inter. cluster the genes up- or downregulated in both NOD and db/db + mSTZ were obtained. We plotted their expression per diabetes model datasets across the adult2, D-inter. and 14–16wNOD (for 8–16wNOD dataset) or db/db + mSTZ (for db/db and mSTZ datasets) clusters. We normalized gene expression across clusters in each dataset to [0,1]. We computed the gene set enrichment of the shared DEGs as for human T1D and T2D genes. The GP differences between adult2 and D-inter. clusters were computed for individual datasets (db/db, mSTZ and 8–16wNOD) as described in the section ‘Fine β-cell states’.

### Differential expression in T1D model and T2D model endocrine cells

To compare DEGs across diabetes models and endocrine cell types we fitted a joint model with edgeR. Cells from healthy adults (datasets 4m, 8–16wNOD samples aged 8 weeks, db/db control, mSTZ control; *n* = 10, some samples contained pooled animals), a T1D model (dataset NOD_progression samples aged 14 and 16 weeks; *n* = 6) and T2D models (datasets mSTZ and db/db, both without treatment; *n* = 3) were used to compute metadata-based pseudobulks per disease status group, sample, dataset, sex and endocrine cell type. Lowly expressed genes were removed with edgeR. A single expression model was fitted, using edgeR GLM with robust dispersion, with dataset and sex as covariates. A two-sided likelihood-ratio test was used to compare model factors for each T1D model or T2D model cell type to the corresponding healthy cell type to obtain the T1D model or T2D model effect per cell type. The DEGs were selected based on FDR < 0.05, absolute logFC > 1 and relative expression in individual cell types >0.1 to focus on genes that are less likely to be ambiently expressed. Overlap between DEGs was computed accounting for DGE direction between the two groups. Same direction DEGs across α-, δ- and γ-cells in both diabetes types were extracted and gene set enrichment was computed as for human T1D and T2D genes.

### Sex differences in β-cells during aging

Two datasets that contained a mixture of male and female cells were used: P16 and aged. Each dataset was analyzed separately; both datasets had three samples per group with pooled animals within samples. Cells from low-quality coarse β-cell clusters, genes expressed in less than 5% of cells and non-β-cell ambient genes were removed. DGE analysis was performed with sex and samples as covariates using diffxpy two-sided Wald test. We removed genes that could not be fitted, as indicated by extremely small standard deviations of the regression coefficient (s.d. 2.2 × 10^−162^). DEGs were selected based on FDR < 0.05 and absolute logFC > 1.

DEGs between sexes in the aged dataset were separated by DGE direction and hierarchically clustered on the whole β-cell fine pseudobulk data. Cutting thresholds were selected visually based on heat map portraying gene expression across fine pseudobulks. All β-cells were scored for DEG groups with the Scanpy score_genes function.

### Laboratory validation of diabetes markers

For diabetes markers validation we used healthy adult mice from strains C57BL/6J (three males and three females, aged 2–4 months) and B6.BKS(D)-Leprdb/J (healthy db/db control), db/db T2D model mice (three males aged 8 weeks) and NOD T1D model mice (three females aged 8 weeks). For endocrine markers validation we used postnatal healthy mice from strain C57BL/6J (two males and one female, at P9 stage). Mice were housed in groups of two to four animals and maintained at 23 ± 1 °C and 45–65% humidity on a 12-h dark–light cycle with ad libitum access to diet (irradiated standard diet for rodents, Altromin 1314, Altromin Spezialfutter) and water.

Mice pancreases were dissected and fixed (4% PFA–PBS, 24 h at 4 °C). The organs were cryoprotected in a sequential gradient of 7.5, 15 and 30% sucrose–PBS solutions (each solution 2 h at room temperature). Next, pancreases were incubated in 30% sucrose and tissue-freezing medium (Leica) (1:1, overnight at 4 °C). Afterward, they were embedded using a tissue-freezing medium. Sections of 20-μm thickness were cut from each sample mounted on a glass slide (Thermo Fisher Scientific).

Islet isolation was performed by collagenase P (Roche) digestion of the adult pancreas. We injected 3 ml collagenase P (1 mg ml^−1^) into the bile duct and the perfused pancreas was consequently dissected and placed into 3 ml collagenase P for 15 min at 37 °C. Then, 10 ml G-solution (HBSS (Lonza) + 1% BSA (Sigma)) was added to the samples followed by centrifugation at 563*g* (Eppendorf Centrifuge 5910R) at 4 °C. After another washing step with G-solution, the pellets were resuspended in 5.5 ml gradient preparation (5 ml 10% RPMI (Lonza) and 3 ml 40% Optiprep (Sigma) per sample) and placed on top of 2.5 ml of the same solution. To form a three-layer gradient, 6 ml G-solution was added on the top. Samples were then incubated for 10 min at room temperature before subjecting to centrifugation at 523*g* (settings were acceleration 3, stopping 0; Eppendorf Centrifuge 5804R). Finally, the interphase between the upper and the middle layers of the gradient was collected and filtered through a 70-μm nylon filter and washed with G-solution. Islets were handpicked under the microscope. For fixation, islets were incubated in 4% PFA–PBS for 15 min at room temperature.

For immunostaining, the cryosections were rehydrated and then permeabilized (0.2% Triton X-100-H_2_O for 30 min at room temperature). Then, the samples were blocked in a blocking solution (PBS, 0.1% Tween-20, 1% donkey serum and 5% FCS for 1 h at room temperature). Primary antibodies (Supplementary Table 13) were incubated for at least 4 h at room temperature followed by three washes with PBX. The samples were then incubated with secondary antibodies (Supplementary Table 13) during 4–5 h of incubation. For the anti-Rbp4 antibody, we performed antigen retrieval with a citric buffer (10 mM sodium citrate and 0.05% Tween-20, pH 6) in addition to the above-described protocol. Finally, the pancreatic sections were stained with 4,6-diamidino-2-phenylindole (1:500 dilution in 1× PBS for 30 min). All images were obtained on a Leica microscope of the type DMI 6000. Images were analyzed using the LAS X v.3.5.6 and/or ImageJ Fiji-Win32 software.

### Reporting summary

Further information on research design is available in the Nature Portfolio Reporting Summary linked to this article.

### Supplementary information

Supplementary Information Supplementary Figs. 1–4, Supplementary Notes 1–11 and legends for Supplementary Tables 1–13.

Reporting Summary

Supplementary Tables 1–13 Collection of Supplementary Tables 1–13, corresponding to legends in the supplementary information file.

## Data Availability

Up-to-date data resource links are available from https://github.com/theislab/mouse_cross-condition_pancreatic_islet_atlas. The two newly generated scRNA-seq datasets, the integrated atlas and the reference mapped embedding of the Feng dataset were deposited to the GEO within super-series GSE211799. The atlas is also available as a cellxgene instance (https://cellxgene.cziscience.com/collections/296237e2-393d-4e31-b590-b03f74ac5070). The scArches model for reference mapping and an example code for reference mapping used for the Feng dataset are available in https://github.com/theislab/mouse_cross-condition_pancreatic_islet_atlas/tree/main/reference_mapping. The following previously published datasets were included into the atlas: GSE132188, GSE161966, GSE128565, GSE174194, GSE144471, GSE117770, GSE142465 (GSM4228185 to GSM4228199). The following previously published datasets were used for validation: GSE83146, GSE137909, GSE148073, GSE81608, GSE198623, GSE81547, GSE86469, GSE124742 (FACS), GSE124742 (patch-seq), GSE164875 (patch-seq), GSE101207, GSE154126 and GSE83139. Gene sets were obtained from MSigDB (v.7.4.1) and ortholog information was obtained from BioMart (Ensembl Genes v.103).

## Code availability

All code is available at https://github.com/theislab/mouse_cross-condition_pancreatic_islet_atlas. This includes both reproducibility code and an example of how new datasets can be mapped onto the atlas.

## References

[1] Tritschler S, Theis FJ, Lickert H, Bottcher A (2017). Systematic single-cell analysis provides new insights into heterogeneity and plasticity of the pancreas. Mol. Metab..

[2] Bornstein SR, Ludwig B, Steenblock C (2022). Progress in islet transplantation is more important than ever. Nat. Rev. Endocrinol..

[3] Gentileschi P, Bianciardi E, Benavoli D, Campanelli M (2021). Metabolic surgery for type II diabetes: an update. Acta Diabetol..

[4] Jain C, Ansarullah, Bilekova S, Lickert H (2022). Targeting pancreatic β cells for diabetes treatment. Nat. Metab..

[5] Bakhti M, Böttcher A, Lickert H (2019). Modelling the endocrine pancreas in health and disease. Nat. Rev. Endocrinol..

[6] Miranda MA, Macias-Velasco JF, Lawson HA (2021). Pancreatic β-cell heterogeneity in health and diabetes: classes, sources, and subtypes. Am. J. Physiol. Endocrinol. Metab..

[7] Benninger RKP, Kravets V (2022). The physiological role of β-cell heterogeneity in pancreatic islet function. Nat. Rev. Endocrinol..

[8] Liu JSE, Hebrok M (2017). All mixed up: defining roles for β-cell subtypes in mature islets. Genes Dev..

[9] Blum B (2012). Functional β-cell maturation is marked by an increased glucose threshold and by expression of urocortin 3. Nat. Biotechnol..

[10] Nishimura W, Takahashi S, Yasuda K (2015). MafA is critical for maintenance of the mature β cell phenotype in mice. Diabetologia.

[11] Roscioni SS, Migliorini A, Gegg M, Lickert H (2016). Impact of islet architecture on β-cell heterogeneity, plasticity and function. Nat. Rev. Endocrinol..

[12] Bader E (2016). Identification of proliferative and mature β-cells in the islets of Langerhans. Nature.

[13] Aguayo-Mazzucato C (2020). Functional changes in β cells during ageing and senescence. Diabetologia.

[14] Avrahami D (2015). Aging-dependent demethylation of regulatory elements correlates with chromatin state and improved β cell function. Cell Metab..

[15] Enge M (2017). Single-cell analysis of human pancreas reveals transcriptional signatures of aging and somatic mutation patterns. Cell.

[16] Helman A (2016). p16(Ink4a)-induced senescence of pancreatic β cells enhances insulin secretion. Nat. Med..

[17] Shrestha S (2022). Aging compromises human islet β cell function and identity by decreasing transcription factor activity and inducing ER stress. Sci. Adv..

[18] Yong HJ, Toledo MP, Nowakowski RS, Wang YJ (2022). Sex differences in the molecular programs of pancreatic cells contribute to the differential risks of type 2 diabetes. Endocrinology.

[19] Kleinert M (2018). Animal models of obesity and diabetes mellitus. Nat. Rev. Endocrinol..

[20] Yzydorczyk, C., Mitanchez, D., Boubred, F. & Simeoni, U. in *Glucose Intake and Utilization in Pre-Diabetes and Diabetes* (eds Watson, R. R. & Dokken, B. B.) 5–20 (Academic Press, 2015).

[21] Camunas-Soler J (2020). Patch-seq links single-cell transcriptomes to human islet dysfunction in diabetes. Cell Metab..

[22] Wang YJ (2016). Single-cell transcriptomics of the human endocrine pancreas. Diabetes.

[23] Oppenländer L (2021). Vertical sleeve gastrectomy triggers fast β-cell recovery upon overt diabetes. Mol. Metab..

[24] Chan JY, Luzuriaga J, Bensellam M, Biden TJ, Laybutt DR (2013). Failure of the adaptive unfolded protein response in islets of obese mice is linked with abnormalities in β-cell gene expression and progression to diabetes. Diabetes.

[25] In’t Veld P (2014). Insulitis in human type 1 diabetes: a comparison between patients and animal models. Semin. Immunopathol..

[26] Fasolino M (2022). Single-cell multi-omics analysis of human pancreatic islets reveals novel cellular states in type 1 diabetes. Nat. Metab..

[27] Thompson PJ (2019). Targeted elimination of senescent β cells prevents type 1 diabetes. Cell Metab..

[28] Marquina-Sanchez B (2020). Single-cell RNA-seq with spike-in cells enables accurate quantification of cell-specific drug effects in pancreatic islets. Genome Biol..

[29] Furman BL (2021). Streptozotocin-induced diabetic models in mice and rats. Curr. Protoc..

[30] Sachs S (2020). Targeted pharmacological therapy restores β-cell function for diabetes remission. Nat. Metab..

[31] Feng Y (2020). Characterizing pancreatic β-cell heterogeneity in the streptozotocin model by single-cell transcriptomic analysis. Mol. Metab..

[32] Wigger L (2021). Multi-omics profiling of living human pancreatic islet donors reveals heterogeneous β cell trajectories towards type 2 diabetes. Nat. Metab..

[33] Chen C-W (2022). Adaptation to chronic ER stress enforces pancreatic β-cell plasticity. Nat. Commun..

[34] Stožer A (2022). From isles of Königsberg to islets of Langerhans: Examining the function of the endocrine pancreas through network science. Front. Endocrinol..

[35] Mawla AM, Huising MO (2019). Navigating the depths and avoiding the shallows of pancreatic islet cell transcriptomes. Diabetes.

[36] Kaestner, K. H. et al. What is a β cell? – chapter I in the Human Islet Research Network (HIRN) review series. *Mol. Metab.* **53**, 101323 (2021). 10.1016/j.molmet.2021.101323 PMC8452767 34416394

[37] Khin P-P, Lee J-H, Jun H-S (2021). A brief review of the mechanisms of β-cell dedifferentiation in type 2 diabetes. Nutrients.

[38] Halban PA (2014). β-cell failure in type 2 diabetes: postulated mechanisms and prospects for prevention and treatment. Diabetes Care.

[39] Sahin GS, Lee H, Engin F (2021). An accomplice more than a mere victim: the impact of β-cell ER stress on type 1 diabetes pathogenesis. Mol. Metab..

[40] Lopez R, Regier J, Cole MB, Jordan MI, Yosef N (2018). Deep generative modeling for single-cell transcriptomics. Nat. Methods.

[41] Luecken MD (2022). Benchmarking atlas-level data integration in single-cell genomics. Nat. Methods.

[42] Lotfollahi M (2022). Mapping single-cell data to reference atlases by transfer learning. Nat. Biotechnol..

[43] Butler A, Hoffman P, Smibert P, Papalexi E, Satija R (2018). Integrating single-cell transcriptomic data across different conditions, technologies, and species. Nat. Biotechnol..

[44] Aviv, R. et al. The human cell atlas. *eLife*10.7554/elife.27041 (2017). 10.7554/eLife.27041 PMC5762154 29206104

[45] Quake SR (2022). A decade of molecular cell atlases. Trends Genet..

[46] Li J (2022). Deep learning of cross-species single-cell landscapes identifies conserved regulatory programs underlying cell types. Nat. Genet..

[47] Chen K (2022). Single-cell RNA-seq transcriptomic landscape of human and mouse islets and pathological alterations of diabetes. iScience.

[48] Sikkema L (2023). An integrated cell atlas of the lung in health and disease. Nat. Med..

[49] van Gurp L (2022). Generation of human islet cell type-specific identity genesets. Nat. Commun..

[50] Lange M (2022). CellRank for directed single-cell fate mapping. Nat. Methods.

[51] Baron, M. et al. A single-cell transcriptomic map of the human and mouse pancreas reveals inter- and intra-cell population structure. *Cell Syst.*10.1016/j.cels.2016.08.011 (2016). 10.1016/j.cels.2016.08.011 PMC5228327 27667365

[52] Xin Y (2016). RNA sequencing of single human islet cells reveals type 2 diabetes genes. Cell Metab..

[53] Dziewulska A, Dobosz AM, Dobrzyn A (2018). High-throughput approaches onto uncover (epi)genomic architecture of type 2 diabetes. Genes.

[54] Tarifeño-Saldivia E (2017). Transcriptome analysis of pancreatic cells across distant species highlights novel important regulator genes. BMC Biol..

[55] Su Y (2012). Novel function of transthyretin in pancreatic α cells. FEBS Lett..

[56] DiGruccio MR (2016). Comprehensive α, β and δ cell transcriptomes reveal that ghrelin selectively activates δ cells and promotes somatostatin release from pancreatic islets. Mol. Metab..

[57] Artner I (2010). MafA and MafB regulate genes critical to β-cells in a unique temporal manner. Diabetes.

[58] Huang R, Bai X, Li X, Wang X, Zhao L (2021). Retinol-binding protein 4 activates STRA6, provoking pancreatic β-cell dysfunction in type 2 diabetes. Diabetes.

[59] Zhang J, McKenna LB, Bogue CW, Kaestner KH (2014). The diabetes gene Hhex maintains δ-cell differentiation and islet function. Genes Dev..

[60] Bastidas-Ponce A (2019). Comprehensive single cell mRNA profiling reveals a detailed roadmap for pancreatic endocrinogenesis. Development.

[61] Bastidas-Ponce A, Scheibner K, Lickert H, Bakhti M (2017). Cellular and molecular mechanisms coordinating pancreas development. Development.

[62] Johansson KA (2007). Temporal control of neurogenin3 activity in pancreas progenitors reveals competence windows for the generation of different endocrine cell types. Dev. Cell.

[63] Byrnes LE (2018). Lineage dynamics of murine pancreatic development at single-cell resolution. Nat. Commun..

[64] Goudet G, Delhalle S, Biemar F, Martial JA, Peers B (1999). Functional and cooperative interactions between the homeodomain PDX1, PBX, and Prep1 factors on the somatostatin promoter*. J. Biol. Chem..

[65] Gao R, Yang T, Zhang Q (2021). δ-Cells: the neighborhood watch in the islet community. Biology.

[66] Stone VM (2014). GPR120 (FFAR4) is preferentially expressed in pancreatic δ cells and regulates somatostatin secretion from murine islets of Langerhans. Diabetologia.

[67] Rorsman P, Huising MO (2018). The somatostatin-secreting pancreatic δ-cell in health and disease. Nat. Rev. Endocrinol..

[68] Strowski MZ, Parmar RM, Blake AD, Schaeffer JM (2000). Somatostatin inhibits insulin and glucagon secretion via two receptors subtypes: an in vitro study of pancreatic islets from somatostatin receptor 2 knockout mice. Endocrinology.

[69] Omar-Hmeadi M, Lund P-E, Gandasi NR, Tengholm A, Barg S (2020). Paracrine control of α-cell glucagon exocytosis is compromised in human type-2 diabetes. Nat. Commun..

[70] Nasteska D, Hodson DJ (2018). The role of β cell heterogeneity in islet function and insulin release. J. Mol. Endocrinol..

[71] Drigo, R. A. E. et al. Aging of human endocrine pancreatic cell types is heterogeneous and sex-specific. Preprint at *bioRxiv*10.1101/729541 (2019).

[72] Chen Y-G, Mathews CE, Driver JP (2018). The role of NOD Mice in type 1 diabetes research: lessons from the past and recommendations for the future. Front. Endocrinol..

[73] Meyerovich K, Ortis F, Allagnat F, Cardozo AK (2016). Endoplasmic reticulum stress and the unfolded protein response in pancreatic islet inflammation. J. Mol. Endocrinol..

[74] Coleman DL (1978). Obese and diabetes: two mutant genes causing diabetes-obesity syndromes in mice. Diabetologia.

[75] Kobayashi K (2000). The db/db mouse, a model for diabetic dyslipidemia: molecular characterization and effects of Western diet feeding. Metabolism.

[76] Lenzen S (2008). The mechanisms of alloxan- and streptozotocin-induced diabetes. Diabetologia.

[77] Eizirik DL, Pasquali L, Cnop M (2020). Pancreatic β-cells in type 1 and type 2 diabetes mellitus: different pathways to failure. Nat. Rev. Endocrinol..

[78] Fang Z (2019). Single-cell heterogeneity analysis and CRISPR screen identify key β-cell-specific disease genes. Cell Rep..

[79] Salinno C (2021). CD81 marks immature and dedifferentiated pancreatic β-cells. Mol. Metab..

[80] Mizusawa N (2022). Identification of protease serine S1 family member 53 as a mitochondrial protein in murine islet β cells. Islets.

[81] Tritschler, S. et al. A transcriptional cross species map of pancreatic islet cells. *Mol. Metab.*10.1016/j.molmet.2022.101595 (2022). 10.1016/j.molmet.2022.101595 PMC9526148 36113773

[82] Xin Y (2018). Pseudotime ordering of single human β-cells reveals states of insulin production and unfolded protein response. Diabetes.

[83] Kiselev VY, Andrews TS, Hemberg M (2019). Challenges in unsupervised clustering of single-cell RNA-seq data. Nat. Rev. Genet..

[84] Brereton, M. F., Rohm, M. & Ashcroft, F. M. β-Cell dysfunction in diabetes: a crisis of identity? *Diabetes Obes. Metab.*10.1111/dom.12732 (2016). 10.1111/dom.12732 PMC5890905 27615138

[85] Farack L (2019). Transcriptional heterogeneity of β cells in the intact pancreas. Dev. Cell.

[86] Rui J (2017). β Cells that resist immunological attack develop during progression of autoimmune diabetes in NOD mice. Cell Metab..

[87] Kang, R. B. et al. Single-nucleus RNA sequencing of human pancreatic islets identifies novel gene sets and distinguishes β-cell subpopulations with dynamic transcriptome profiles. *Genome Med.* **15**, 30 (2023). 10.1186/s13073-023-01179-2 PMC10150516 37127706

[88] Chu CMJ (2022). Dynamic Ins2 gene activity defines β-cell maturity states. Diabetes.

[89] Tonne JM (2013). Global gene expression profiling of pancreatic islets in mice during streptozotocin-induced β-cell damage and pancreatic Glp-1 gene therapy. Dis. Model. Mech..

[90] Päth G (2020). NUPR1 preserves insulin secretion of pancreatic β-cells during inflammatory stress by multiple low-dose streptozotocin and high-fat diet. Am. J. Physiol. Endocrinol. Metab..

[91] Puddu A (2013). Update on the protective molecular pathways improving pancreatic β-cell dysfunction. Mediators Inflamm..

[92] Juliana CA (2017). ATF5 regulates β-cell survival during stress. Proc. Natl Acad. Sci. USA.

[93] Atkinson MA, Campbell-Thompson M, Kusmartseva I, Kaestner KH (2020). Organisation of the human pancreas in health and in diabetes. Diabetologia.

[94] Adams MT, Blum B (2022). Determinants and dynamics of pancreatic islet architecture. Islets.

[95] Carrano AC, Mulas F, Zeng C, Sander M (2017). Interrogating islets in health and disease with single-cell technologies. Mol. Metab..

[96] Noguchi GM, Huising MO (2019). Integrating the inputs that shape pancreatic islet hormone release. Nat. Metab..

[97] Dai X-Q (2022). Heterogenous impairment of α cell function in type 2 diabetes is linked to cell maturation state. Cell Metab..

[98] Drigo RAE (2019). Structural basis for δ cell paracrine regulation in pancreatic islets. Nat. Commun..

[99] Rodnoi P (2017). Neuropeptide Y expression marks partially differentiated β cells in mice and humans. JCI Insight.

[100] Jacovetti C, Regazzi R (2022). Mechanisms underlying the expansion and functional maturation of β-cells in newborns: impact of the nutritional environment. Int. J. Mol. Sci..

[101] Dalmas E (2019). Innate immune priming of insulin secretion. Curr. Opin. Immunol..

[102] Li Y (2021). Revisiting the antigen-presenting function of β cells in T1D pathogenesis. Front. Immunol..

[103] Aguayo-Mazzucato C (2019). Acceleration of β cell aging determines diabetes and senolysis improves disease outcomes. Cell Metab..

[104] Kokkinopoulou I, Diakoumi A, Moutsatsou P (2021). Glucocorticoid receptor signaling in diabetes. Int. J. Mol. Sci..

[105] Stephens SB (2012). A VGF-derived peptide attenuates development of type 2 diabetes via enhancement of islet β-cell survival and function. Cell Metab..

[106] Gurgul-Convey E (2020). Sphingolipids in type 1 diabetes: focus on β-cells. Cells.

[107] Furuhashi M (2020). Independent and distinct associations of FABP4 and FABP5 with metabolic parameters in type 2 diabetes mellitus. Front. Endocrinol..

[108] Martens GA (2011). Clusters of conserved β cell marker genes for assessment of β cell phenotype. PLoS ONE.

[109] Van de Velde, S. et al. CREB promotes β cell gene expression by targeting its coactivators to tissue-specific enhancers. *Mol. Cell. Biol.* **39**, e00200-19 (2019). 10.1128/MCB.00200-19 PMC6692124 31182641

[110] Leu S-Y (2020). Loss of EGR-1 uncouples compensatory responses of pancreatic β cells. Theranostics.

[111] Hong K, Xu G, Grayson TB, Shalev A (2016). Cytokines regulate β-cell thioredoxin-interacting protein (TXNIP) via distinct mechanisms and pathways. J. Biol. Chem..

[112] Puri S, Folias AE, Hebrok M (2015). Plasticity and dedifferentiation within the pancreas: development, homeostasis, and disease. Cell Stem Cell.

[113] Kim-Muller JY (2016). Aldehyde dehydrogenase 1a3 defines a subset of failing pancreatic β cells in diabetic mice. Nat. Commun..

[114] Cinti F (2016). Evidence of β-cell dedifferentiation in human type 2 diabetes. J. Clin. Endocrinol. Metab..

[115] Yang Y (2019). Islet β-cell-produced NUCB2/nesfatin-1 maintains insulin secretion and glycemia along with suppressing UCP-2 in β-cells. J. Physiol. Sci..

[116] Maejima Y (2017). Nesfatin-1 inhibits voltage gated K+ channels in pancreatic beta cells. Peptides.

[117] Li X-S, Yan C-Y, Fan Y-J, Yang J-L, Zhao S-X (2020). NUCB2 polymorphisms are associated with an increased risk for type 2 diabetes in the Chinese population. Ann. Transl. Med.

[118] Lu H, Yang Y, Allister EM, Wijesekara N, Wheeler MB (2008). The identification of potential factors associated with the development of type 2 diabetes: a quantitative proteomics approach. Mol. Cell. Proteom..

[119] Hartley T (2010). Endoplasmic reticulum stress response in an INS-1 pancreatic β-cell line with inducible expression of a folding-deficient proinsulin. BMC Cell Biol..

[120] Byun H-R, Choi JA, Koh J-Y (2014). The role of metallothionein-3 in streptozotocin-induced β-islet cell death and diabetes in mice. Metallomics.

[121] Viñuela A (2020). Genetic variant effects on gene expression in human pancreatic islets and their implications for T2D. Nat. Commun..

[122] Shrestha N, De Franco E, Arvan P, Cnop M (2021). Pathological β-cell endoplasmic reticulum stress in type 2 diabetes: current evidence. Front. Endocrinol..

[123] Wang, S., Flibotte, S., Camunas-Soler, J., MacDonald, P. E. & Johnson, J. D. A new hypothesis for type 1 diabetes risk: the at-risk allele at rs3842753 associates with increased β-cell INS messenger RNA in a meta-analysis of single-cell RNA-sequencing data. *Can. J. Diabetes*10.1016/j.jcjd.2021.03.007 (2021). 10.1016/j.jcjd.2021.03.007 34052132

[124] Cefalu WT (2022). Heterogeneity of diabetes: β-cells, phenotypes, and precision medicine: proceedings of an international symposium of the Canadian Institutes of Health Research’s Institute of Nutrition, Metabolism and Diabetes and the US National Institutes of Health’s National Institute of Diabetes and Digestive and Kidney Diseases. Diabetes Care.

[125] Gurzov EN, Ortis F, Bakiri L, Wagner EF, Eizirik DL (2008). JunB inhibits ER stress and apoptosis in pancreatic β cells. PLoS ONE.

[126] Gannon M, Kulkarni RN, Tse HM, Mauvais-Jarvis F (2018). Sex differences underlying pancreatic islet biology and its dysfunction. Mol. Metab..

[127] Brownrigg, G. P. et al. Sex differences in islet stress responses support female β cell resilience. *Mol. Metab.* **9**, 101678 (2023). 10.1016/j.molmet.2023.101678 PMC9971554 36690328

[128] Liu G (2021). Single-cell RNA sequencing reveals sexually dimorphic transcriptome and type 2 diabetes genes in mouse islet β cells. Genom. Proteom. Bioinform..

[129] Makino S (1980). Breeding of a non-obese, diabetic strain of mice. Jikken Dobutsu.

[130] Tramunt B (2020). Sex differences in metabolic regulation and diabetes susceptibility. Diabetologia.

[131] Viloria K (2020). Vitamin-d-binding protein contributes to the maintenance of α cell function and glucagon secretion. Cell Rep..

[132] Cabrera O (2008). Glutamate is a positive autocrine signal for glucagon release. Cell Metab..

[133] Szabat M (2011). Kinetics and genomic profiling of adult human and mouse β-cell maturation. Islets.

[134] Layden BT (2010). Regulation of pancreatic islet gene expression in mouse islets by pregnancy. J. Endocrinol..

[135] Bearrows SC (2019). Chromogranin B regulates early-stage insulin granule trafficking from the Golgi in pancreatic islet β-cells. J. Cell Sci..

[136] Sidarala V, Kowluru A (2017). The regulatory roles of mitogen-activated protein kinase (MAPK) pathways in health and diabetes: lessons learned from the pancreatic β-cell. Recent Pat. Endocr. Metab. Immune Drug Discov..

[137] Chang C, Worley BL, Phaëton R, Hempel N (2020). Extracellular glutathione peroxidase GPx3 and its role in cancer. Cancers.

[138] Lebrun P (2010). The suppressor of cytokine signalling 2 (SOCS2) is a key repressor of insulin secretion. Diabetologia.

[139] Zhang Y (2017). Glucose potentiates β‐cell function by inducing Tphl expression in rat islets. FASEB J..

[140] Bertolino P (2008). Activin B receptor ALK7 is a negative regulator of pancreatic β-cell function. Proc. Natl Acad. Sci..

[141] Berger C, Zdzieblo D (2020). Glucose transporters in pancreatic islets. Pflug. Arch..

[142] Bunik VI, Degtyarev D (2008). Structure-function relationships in the 2-oxo acid dehydrogenase family: substrate-specific signatures and functional predictions for the 2-oxoglutarate dehydrogenase-like proteins. Proteins.

[143] Megill, C. et al. cellxgene: a performant, scalable exploration platform for high dimensional sparse matrices. Preprint at *bioRxiv*10.1101/2021.04.05.438318 (2021).

[144] Tiriveedhi V (2018). Impact of precision medicine on drug repositioning and pricing: a too small to thrive crisis. J. Pers. Med..

[145] Linsley PS, Greenbaum CJ, Nepom GT (2021). Uncovering pathways to personalized therapies in type 1 diabetes. Diabetes.

[146] Unnikrishnan R, Radha V, Mohan V (2021). Challenges Involved in incorporating personalised treatment plan as routine care of patients with diabetes. Pharmgenom. Pers. Med..

[147] Fischer, D. S. et al. Sfaira accelerates data and model reuse in single cell genomics. *Genome Biol.*10.1186/s13059-021-02452-6 (2021). 10.1186/s13059-021-02452-6 PMC8386039 34433466

[148] Li D-S, Yuan Y-H, Tu H-J, Liang Q-L, Dai L-J (2009). A protocol for islet isolation from mouse pancreas. Nat. Protoc..

[149] Corbin KL (2021). A practical guide to rodent islet isolation and assessment revisited. Biol. Proced. Online.

[150] Lange A (2012). Fltp(T2AiCre): a new knock-in mouse line for conditional gene targeting in distinct mono- and multiciliated tissues. Differentiation.

[151] Gegg, M. et al. Flattop regulates basal body docking and positioning in mono- and multiciliated cells. *eLife*10.7554/elife.03842 (2014). 10.7554/eLife.03842 PMC4221739 25296022

[152] Wang X, He Y, Zhang Q, Ren X, Zhang Z (2021). Direct comparative analyses of 10x genomics chromium and Smart-seq2. Genom. Proteom. Bioinform..

[153] Wolf FA, Angerer P, Theis FJ (2018). SCANPY: large-scale single-cell gene expression data analysis. Genome Biol..

[154] Kinsella RJ (2011). Ensembl BioMarts: a hub for data retrieval across taxonomic space. Database.

[155] Zheng GXY (2017). Massively parallel digital transcriptional profiling of single cells. Nat. Commun..

[156] Lun ATL (2019). EmptyDrops: distinguishing cells from empty droplets in droplet-based single-cell RNA sequencing data. Genome Biol..

[157] Luecken MD, Theis FJ (2019). Current best practices in single-cell RNA-seq analysis: a tutorial. Mol. Syst. Biol..

[158] Wolock SL, Lopez R, Klein AM (2019). Scrublet: computational identification of cell doublets in single-cell transcriptomic data. Cell Syst..

[159] Lun, A. T. L., McCarthy, D. J. & Marioni, J. C. A step-by-step workflow for low-level analysis of single-cell RNA-seq data with Bioconductor. *F1000Research*10.12688/f1000research.9501.2 (2016). 10.12688/f1000research.9501.1 PMC5112579 27909575

[160] Lun ATL, Bach K, Marioni JC (2016). Pooling across cells to normalize single-cell RNA sequencing data with many zero counts. Genome Biol..

[161] Scialdone A (2015). Computational assignment of cell-cycle stage from single-cell transcriptome data. Methods.

[162] Fleming, S. J. et al. Unsupervised removal of systematic background noise from droplet-based single-cell experiments using CellBender. *Nat. Methods*10.1038/s41592-023-01943-7 (2023). 10.1038/s41592-023-01943-7 37550580

[163] Young MD, Behjati S (2020). SoupX removes ambient RNA contamination from droplet-based single-cell RNA sequencing data. Gigascience.

[164] Yang S (2020). Decontamination of ambient RNA in single-cell RNA-seq with DecontX. Genome Biol..

[165] Traag VA, Waltman L, van Eck NJ (2019). From Louvain to Leiden: guaranteeing well-connected communities. Sci. Rep..

[166] Baran Y (2019). MetaCell: analysis of single-cell RNA-seq data using K-nn graph partitions. Genome Biol..

[167] Persad, S. et al. SSEACells infers transcriptional and epigenomic cellular states from single-cell genomics data. *Nat. Biotechnol.*10.1038/s41587-023-01716-9 (2023). 10.1038/s41587-023-01716-9 PMC10713451 36973557

[168] Squair JW (2021). Confronting false discoveries in single-cell differential expression. Nat. Commun..

[169] Robinson MD, McCarthy DJ, Smyth GK (2010). edgeR: a Bioconductor package for differential expression analysis of digital gene expression data. Bioinformatics.

[170] Federico A, Monti S (2020). hypeR: an R package for geneset enrichment workflows. Bioinformatics.

[171] Jupp S, Burdett T, Leroy C, Parkinson HE (2015). A new ontology lookup service at EMBL-EBI. SWAT4LS.

[172] Gu Z, Eils R, Schlesner M (2016). Complex heatmaps reveal patterns and correlations in multidimensional genomic data. Bioinformatics.

[173] Gu, Z. Complex heatmap visualization. *iMeta*10.1002/imt2.43 (2022). 10.1002/imt2.43 PMC10989952 38868715

[174] Burkhardt DB (2021). Quantifying the effect of experimental perturbations at single-cell resolution. Nat. Biotechnol..

[175] Fischer, D. diffxpy. https://diffxpy.readthedocs.io/en/latest/index.html (2020).

[176] Lee H (2020). β Cell dedifferentiation induced by IRE1α deletion prevents type 1 diabetes. Cell Metab..

[177] Xin Y (2016). Single-cell RNAseq reveals that pancreatic β-cells from very old male mice have a young gene signature. Endocrinology.

[178] Lawlor N (2017). Single-cell transcriptomes identify human islet cell signatures and reveal cell-type-specific expression changes in type 2 diabetes. Genome Res..

[179] Avrahami D (2020). Single-cell transcriptomics of human islet ontogeny defines the molecular basis of β-cell dedifferentiation in T2D. Mol. Metab..

