# Supplementary material for: Delineating mouse β-cell identity during lifetime and in diabetes with a single cell atlas
Source: Nat Metab. 2023 Sep 11;5(9):1615–37. doi: 10.1038/s42255-023-00876-x (PMC10513934; doi:10.1038/s42255-023-00876-x)
Supplement: Supplementary file 1 — Supplementary Figs. 1–4, Supplementary Notes 1–11 and legends for Supplementary Tables 1–13. [file 42255_2023_876_MOESM1_ESM.pdf]

nature metabolism

Article

https://doi.org/10.1038/s42255-023-00876-x

Delineating mouse β-cell identity during lifetime and in diabetes with a single cell atlas

In the format provided by the authors and unedited

Supplementary materials

Supplementary notes

Supplementary Note 1: Integration optimization

On the non-integrated embedding we observed strong separation between endocrine cell types across datasets (**Supplementary Figure 1a**). For example, γ-cells were located in two distinct regions across datasets. They did not even co-localize among in-house datasets; i.e., P16 and 4m datasets located separately from aged, mSTZ, and db/db datasets. As these differences could not be fully explained by biological differences after visual analysis of available metadata, we assumed that they are also driven by technical factors, which would bias our interpretations if we used the embedding without batch effect removal. Thus, we decided to perform integration, as recommended when analyzing multiple datasets at once[1–3].

For the integration of the atlas, we tested cVAE model implemented in the scArches package[4] and scVI method[5] from scvi-tools, as it was recently reported that neural network based integration methods are most suitable for large and complex dataset collections, such as ours[1]. We observed stronger batch correction by scVI and comparable biological conservation on the level of cell types between cVAE and scVI integrations (**Supplementary Figure 1c**). As we were particularly interested in β-cell analysis we also evaluated integration on the level of β-cell states defined with known markers (**Supplementary Figure 1c**). Since scVI-based integrations had much lower biological variation preservation within β-cells we decided to use cVAE to enable exploration of biological heterogeneity within β-cells. We observed strong sample-specific ambient RNA contamination (**Supplementary Figure 1b**), possibly due to different cell type proportions across samples, introducing additional batch effects. Thus, we aimed to improve integration performance by preprocessing with various ambient-contamination removal methods. Interestingly, we found that preprocessing with ambient removal tools (DecontX[6], SoupX[7]) did not improve integration beyond the exclusion of genes with the strongest ambient expression contribution. Thus, we decided to use ambient gene exclusion alone for the final integration (**Supplementary Figure 1c**).

Furthermore, we also attempted to specifically improve β-cell integration by performing integration of β-cells only. We assumed that by integrating only β-cells we could remove more of the ambient effects. That is, the HVG selection and thus the integration would no longer be affected by the variability of markers from other cell types. However, in the β-cell specific integration we were unable to achieve matched biological preservation at the same batch correction level as in the whole-islet integration (**Supplementary Figure 1d**). Thus, we did not use it further. Better integration of all cell types at once could be explained by different factors, such as a more diverse set of genes used for integration that improves biological variation preservation or by learning batch effects across cell types, thus being better able to separate technical effects from biological variation that may be more cell type specific. For example, in β-cells alone, some biological states are sample-specific, resulting in direct confounding of batch and biological variation, while when using multiple cell types, the batch effects can be more easily disentangled from biological variability at the cell type level.

Supplementary Note 2: Expression of human diabetes-associated gene sets across mouse diabetes models

To compare mouse diabetes models with human diabetes we plotted for mouse data the expression of gene sets associated with human diabetes, including here-identified (see methods) and previously reported gene sets (**Figure 4e**, significance is reported in **Supplementary Table 4**). Gene set "transport vesicle membrane" was enriched in both our human T1D and T2D upregulated genes and was accordingly upregulated in all three mouse models. Genes upregulated in our human T1D DGE analysis were enriched for immune gene sets, as reported previously[8], including "MHC class I protein complex" and "antimicrobial humoral response". Both of these gene sets had a larger fold change between healthy and diabetic β-cells in the NOD than in the mSTZ or db/db. Among the gene sets enriched in the human T2D upregulated genes were gene sets associated with regulation of response to extracellular stimuli and with hormone metabolic processes, both of which were upregulated in the db/db and mSTZ models, but not in the NOD model. We further supplement our analysis with gene sets previously reported to be upregulated in diabetic β-cells[8–10], including gene sets indicative of β-cell compensation in T2D[8,10]. These gene sets (oxidative phosphorylation, ribosome biogenesis, regulation of cellular response to hypoxia, and proteasomal protein catabolic process) were upregulated in db/db and mSTZ, but not NOD diabetes model β-cells. In **Supplementary Note 3** we discuss the limitation of our gene set comparison when the gene set contains too diverse genes that are associated with opposing molecular processes.

Supplementary Note 3: Limitation of cell group comparison with gene set scores

To compare molecular characteristics of diabetes in human and mouse models we collected gene sets upregulated in human diabetes followed by comparison expression of these gene sets in mouse healthy controls and diabetic samples. While this method produced expected results in mouse diabetes models for most gene sets (explained in **Supplementary Note 2**) we observed some unexpected discrepancies between the two species and also current literature. The gene set "endocrine pancreas development" was enriched among human T2D-upregulated genes (**Supplementary Table 4**), which raises the expectation that its mouse orthologues would also have a higher overall expression in the mouse T2D models compared to healthy controls (**Figure 4e**). However, the gene set was downregulated in the db/db and mSTZ models (T2D) and not significantly dysregulated in the NOD model (T1D). This is not in agreement with current literature as T2D related dedifferentiation was before observed in both mouse and human[9,11,12]. The unexpected direction of the gene set change in the db/db and mSTZ models may be explained by the gene set "endocrine pancreas development" containing both maturation (e.g., *Pax6*, *Pdx1*[12]) and immaturity (e.g., *Neurod1*, *Neurog3*[13]) marker genes, which likely affects the score in the mouse cells. This shows a limitation of our gene set based analysis when the selected gene set contains too diverse genes.

Supplementary Note 4: Description of coarse β-cell states and their markers

We resolved seven coarse states based on metadata information as well as two clusters that are likely low-quality populations, characterized by poor quality metric scores (**Extended Data Figure 5a**), which we regarded as technical artifacts and hence did not use in the downstream β-cell coarse state marker identification. Most of the cell states contained cells from multiple datasets (**Extended Data Figure 5c, Supplementary Table 2**). Due to the high transcriptomic similarity between mSTZ and db/db diabetic β-cells, as described in the section "Transcriptomic similarity of db/db and STZ diabetes model β-cells", they were annotated as a single T2D model state. The immature state contained cells ranging from P16 to adult, but was characterized by high expression of known immaturity marker genes, such as *Rbp4* and *Mafb*[14] (**Figure 5b**). Two healthy datasets of different ages contained mixed sex samples ("P16" - postnatal day 16, "aged" - 2 years old). Separation of cells by sex was observed only in the aged dataset (**Extended Data Figure 5f**), as expected since the P16 dataset was generated from sexually immature cells before puberty. Furthermore, we observed that β-cells from the chem dataset mapped separately from other

datasets (**Figure 4a**, **Figure 5a**, **Extended Data Figure 5c**), likely due to *in vitro* islet cultivation or ambient effect of the spike-in RNA used in the protocol[15].

We used MIA to identify markers that are both specific for an individual β-cell state and conserved across all datasets mapping to that state by applying one-vs-rest DGE between cell states and retaining the intersection of markers across datasets (**Figure 5c**, **Extended Data Figure 6a**, **Supplementary Table 5**), with each state containing one to four datasets (**Extended Data Figure 6b**). We observed a low number of cell state markers for some states, such as adult and immature states (**Extended Data Figure 6b**). This may be due to the stringent requirement of markers being consistent in four datasets mapping into these states. However, this ensures high robustness. We also observed on a per-dataset level that certain cell states, including the adult state, had fewer markers, indicating that these states are less molecularly unique (**Extended Data Figure 6b**). Reliable detection of these states would likely benefit from a combination of positive and negative markers. However, we found a large number of db/db+mSTZ state markers that were conserved across both datasets mapping into this state (**Extended Data Figure 6b**), indicating the strength of molecular changes involved in the T2D model dysfunction.

Supplementary Note 5: Comparison of β-cell heterogeneity within MIA with previous literature

As β-cell heterogeneity has been extensively described before[16], we decided to compare our results to key publications in the field. Gradual maturation of β-cells after birth was previously characterized based on the upregulation of *Ucn3* and downregulation of *Mafb* by Zeng et al. (2017) and based on the downregulation of *Cd81* by Salinno (2021). Accordingly, we observed higher expression of *Mafb* and *Cd81* and lower expression of *Ucn3* in our immature state (imm.) from younger mice compared to the healthy adult state (adult, **Figure 5b**). Furthermore, our GP23, which contained multiple immaturity marker genes (*Rbp4*, *Mafb*), was gradually downregulated from immature to adult states (imm.1 and imm.2, adult-imm.1, and adul1 and adul2, **Extended Data Figure 8b**). However, multiple genes that were reported to be downregulated during maturation by Zeng et al. (2017) (*Fos*, *Fosb*, *Egr1*, *Junb*, *Jun*, *Txnip*) were present in our aging-associated healthy gene group 4 (**Supplementary Table 8**), showing some discrepancy between exact maturation-associated genes. It was also reported that proliferative β-cells decline after birth[17] and indeed, we observed the highest proportion of proliferative endocrine cells in our youngest postnatal dataset (P16, **Extended Data Figure 2**). Moreover, Aguayo-Mazzucato et al. (2017) reported an increase of senescence marker *Cdkn2a* during aging, as was also observed in our aged states (agedF and agedM, **Figure 5b**). Maturity and aging heterogeneity were previously also observed within individual samples[17,18]. This corresponds to our finding that all healthy adult samples contain subpopulations of cells with high expression of immaturity or aging-related genes (healthy variable gene groups 2 and 4, **Supplementary Note 7**). However, our integrated embedding did not show separation between cell populations sorted for maturity marker Flattop (**Extended Data Figure 4**, datasets: P16, 4m, aged), which was discovered by Bader et al. (2016)[19] and similarly we did not observe a strong difference in *Cfap126* (gene encoding Flattop protein) mRNA expression between sorted populations (**Extended Data Figure 5g**). This is expected as *Cfap126* is expressed only transiently and at low levels, while both of the reporters mark the cells even after *Cfap126* is no longer expressed[20,21].

Xin et al. (2018) reported cycling of cells between states of active insulin production, resulting in metabolic stress, followed by a recovery phase. We observed similar metabolic heterogeneity in healthy adult samples, as described in the section "β-cell dysfunction patterns within healthy samples".

Oppenländer et al. (2021) and Sachs et al. (2020), whose data is contained within MIA, described db/db and mSTZ model populations, respectively, distinct from healthy controls. Their diabetic model populations match our db/db+mSTZ population (**Extended Data Figure 5c,d**) and we detected molecular

changes corresponding to previous publications (**Supplementary Note 6**). Similarly, markers *Ucn3* and *Slc2a2* were reported to be downregulated in the STZ model by Feng et al. (2020) and were also downregulated in our diabetic db/db+mSTZ population (**Figure 5b**). However, the orthologue of gene *ST8SIA1*, which was reported to be upregulated in human T2D by Dorrell et al. (2016), was significantly downregulated in our db/db+mSTZ DGE analysis (**Supplementary Table 10**). Conversely, orthologue of their marker *CD9*, for which they did not discuss association with diabetes[22], was upregulated in our db/db+mSTZ DGE analysis. Our integration also preserved finer cellular heterogeneity. The trajectory between mSTZ model cells with and without anti-diabetic treatment and controls, which was reported in the original study[11], was also observed in MIA (**Supplementary Note 8**). Similarly, Oppenländer et al. (2021) reported differences between db/db model cells with and without VSG and we observed a separation of VSG-treated cells into a distinct cluster (db/db-VSG state, **Extended Data Figure 5d**). Thompson et al. (2019), whose data is also contained within MIA, described a distinct cell population during NOD model diabetes progression, matched to our NOD-D population (**Figure 5a, Extended Data Figure 5c, Supplementary Note 6**). As expected, based on their reports, our DGE analysis of the NOD model revealed upregulation of senescence markers *Cdkn1a* and *Cxcl10* (**Supplementary Table 10**).

While we largely observe correspondence with previous studies in terms of markers and subpopulations, we also report some results that we could not reproduce. Having a comprehensive atlas that enables quick lookup of genes and cells, such as MIA, thus helps to reveal findings that do not translate to other datasets.

Supplementary Note 6: Comparison of healthy and diabetic states with GPs

To showcase our GP-based interpretation approach we applied it to cell states that have been compared before[11,12,23]. Namely, we compared the main healthy state (adult2) to the main T2D model state (db/db+mSTZ) for the db/db and mSTZ datasets and to the T1D model state (NOD-D) for the 8-16wNOD dataset (**Extended Data Figure 8c**). We selected the adult2 and db/db+mSTZ states as the main healthy and T2D model states, respectively, based on them containing cells across datasets for specific conditions. According to our observations in the diabetes models comparison (section "Transcriptomic similarity of db/db and STZ diabetes model β-cells"), the GP differences were similar for db/db and mSTZ diabetes model cells and more distinct for the NOD model (**Extended Data Figure 8c**). Among the most differentially activated GPs in the diseased state of the db/db and mSTZ models were downregulated GP19, enriched in genes related to insulin or peptide hormone secretion, GP20, enriched in genes associated with stimuli (growth factors, hormones, cytokines) response, and GP17, enriched for p38 MAPK cascade. On the other hand, the diseased state of the db/db and mSTZ models exhibited upregulation of GP3, which is predominantly active in the T2D-model cells (**Extended Data Figure 8b**) and contains known T2D-associated genes (*Cck*, *Gc*, *Slc5a10*, and *Gast*)[12], and GP4, enriched in protein processing and golgi-ER endomembrane system. In the NOD diabetic state, we observed the strongest change in upregulation of GP27 (enriched for antigen presentation and interferon response) and GP25 (enriched for response to viruses). In all comparisons of diabetes model states there was downregulation of GP15, enriched for the circadian clock, insulin secretion, and stimuli response genes. Altogether, this shows that GPs provide relevant insights into differences between cell states, which can be broadly summarized by immune-mediated β-cell dysfunction in the NOD T1D model and metabolic/stress-related dysfunction in the db/db and mSTZ T2D models[24].

Supplementary Note 7: Description of gene groups consistently variable in healthy samples

Gene groups consistently variable across health samples (**Supplementary Table 8**) were interpreted based on gene set enrichment and group member genes (**Supplementary Table 8**), correlation with known β-cell function genes (**Figure 5g**), and expression across MIA β-cells (**Extended Data Figure 9b,c**), as

summarized in **Supplementary Table 8**. Groups 3 and 5 had similar expression patterns in healthy cells (**Extended Data Figure 9a**) and were associated with β-cell maturity and insulin production, with group 3 being more strongly associated with insulin-production-related stress and more strongly active in diabetes model states (**Figure 5g, Extended Data Figure 9b**, **Supplementary Table 8**). Group 1, which was highly active in cells with low activity of groups 3 and 5 (**Extended Data Figure 9a**), was negatively correlated with the expression of many insulin-production-related genes and contained genes previously implicated in β-cell recovery from metabolic stress, such as ATP-production-related genes[25], and protective genes (**Figure 5g, Supplementary Table 8**). Group 2 contained immaturity genes (**Figure 5g**), and was more strongly expressed in immature states of MIA and in healthy adult samples with a higher proportion of immature cells (**Extended Data Figure 9b,c, Extended Data Figure 5c**). Its high activity in healthy adult cells was mutually exclusive with high activities of groups 1, 4, and 5, and to a lesser extent of group 3 (**Extended Data Figure 9a**), indicating a presence of a distinct immature population in healthy adults. Group 4 contained stress and senescence-associated genes (**Figure 5g, Supplementary Table 8**) and had increased activity in the aged and more mature cells from MIA (**Extended Data Figure 9b,c**).

We assessed the localization of cells with the highest expression of individual gene groups in healthy adult samples of the atlas and Feng dataset mapped onto the atlas (**Extended Data Figure 9d**). We observed that cells with high expression of individual gene groups localized to distinct regions of the UMAP embedding, with cells high in groups 3 and 5 showing somewhat greater overlap, as expected based on expression similarity of these two gene groups (**Extended Data Figure 9a**). In all samples cells from the aged-like gene group (4) co-localized near the aged cells (agedM β-cell coarse state), confirming the relevance of this gene group in aging. In general, group 1 (low insulin expression) high-scoring cells localized somewhat further from diabetes model cells than cells high in groups 2, 3, and 5. However, the exact regions of the group-high cells differed between 4m dataset and mSTZ and db/db datasets, as expected due to the different localization of these datasets in the integrated embedding. Especially in the mSTZ, db/db, and Feng datasets we observed a large number of group 3 and also group 5 high cells that localized near the diabetic intermediate cells (D-inter. fine state), confirming the association between stressed or diabetic-like phenotype and increased insulin production. Furthermore, all datasets contained gene group 2 high cells that co-localized with imm.3 and mSTZ fine states. In Feng et al. (2020) it was proposed that a distinct β-cell population within their dataset represents "virgin" β-cells that are located in the islet periphery, but whose function is not fully understood[26]. This "virgin" population within the Feng dataset corresponds to our gene group 2 high cells within imm.3 and mSTZ fine states (**Extended Data Figure 9d,e**). Similarly, gene group 2 activity is anticorrelated to genes downregulated in "virgin" β-cells (*Ucn3*, *Ero1b, G6pc2, Mafa*) and correlated to genes upregulated in "virgin" β-cells (*Mafb* and other immaturity markers, **Figure 5g**)[27,28]. Interestingly, this indicates a similarity between "virgin" and a subset of mSTZ-model β-cells. Additionally, the db/db dataset also contained some gene group 2 high cells that mapped near the immature coarse state (imm.) and the gene group 2 high cells from the 4m dataset likewise mapped to both mSTZ-model and immature regions, indicating the presence of different immature populations in some of the healthy adult samples.

Supplementary Note 8: Trajectories used in diabetic DGE analysis

Dysfunctional NOD samples were relatively young (14 and 16 weeks) and thus the full dysfunctional phenotype might not yet have been developed[29]. Nevertheless, a recent study of T1D and autoantigen-positive prediabetic human donors revealed that pre-diabetic samples already show similar transcriptomic changes to those with progressed T1D[30], indicating that despite the youth of our NOD samples they may nevertheless provide valuable insights into diabetes dysfunction.

A gradient of cells from healthy to dysfunctional was observed in both NOD and db/db+mSTZ mice, which we modeled as a linear trajectory based on the localization of samples and their metadata using MELD[31] (**Extended Data Figure 10a**). We confirmed the validity of both fitted trajectories. In NOD cells the trajectory corresponded to *B2m* expression, a known marker of T1D[32]. In db/db+mSTZ trajectory the cells from diabetes model animals receiving anti-diabetic treatment (VSG and insulin administration) had lower dysfunction scores[11,12] (**Extended Data Figure 10a**).

Supplementary Note 9: Groups of DEGs in T2D model support the separation of mSTZ model cells into two distinct populations

We described that db/db and mSTZ models co-localize on the atlas embedding and show their molecular similarity, however, we also identified heterogeneity within the mSTZ model. Similarly, in our T2D model DGE analysis, some DEG groups were differently strongly dysregulated across T2D model states, while still showing differences against healthy adult cells (**Figure 6a**). The mSTZ cell state had higher activity of the gene group T2-up1 that was also highly expressed in immature cells. In contrast, db/db+mSTZ cell state had higher activity of T2-up2 that was lowly expressed across healthy cells and was enriched for transmembrane ion transport and cell-cell adhesion, which were previously associated with insulin secretion and diabetic compensatory response[8,33]. Cell state db/db+mSTZ also had stronger downregulation of T2-down6 that contained genes involved in key β-cell functions (**Supplementary Table 10**) and whose downregulation was previously linked to β-cell stress[34–37]. This is in accordance with the differences between the two states that we described above based on GPs, with the db/db+mSTZ state showing stronger signs of β-cell metabolic stress than the mSTZ state.

Supplementary Note 10: Validation of cell states in islets used for immunohistochemistry

We validated that the islets used for immunohistochemistry-based validation of diabetes markers contain the expected healthy and dysfunctional cell states in health and diabetes models, respectively. As expected, insulin-positive cells were reduced in both diabetic models (NOD, db/db) compared to control. Ucn3 protein was also strongly downregulated in the db/db model and to a lesser extent in the NOD model compared to healthy control on both the protein and RNA levels. Aldh1a3 protein-positive cells were observed only in the diabetes models (NOD and db/db) and not in the healthy control, however, we did not observe upregulation at the RNA level in NOD islets.

Supplementary Note 11: Gene program differences between the healthy and the intermediate state resemble changes in diabetes

To decipher what distinguishes the intermediate state (D-inter.) from the main healthy state (adult2) we again used GP score differences for each individual diabetes model dataset (**Extended Data Figure 8e**). The GP differences exhibited a pattern similar to the differences between healthy and diabetes model states (**Extended Data Figure 8c**), but with a lower magnitude. Relatively strong differences were also observed in some GPs with similar changes across all diabetes models. Examples are D-inter. downregulated GP15 (enriched in insulin secretion, stimuli response, and circadian clock (**Supplementary Table 6**)) and GP23 (characteristic for immature samples and enriched in cholesterol metabolism, cell junctions, and response to metal ions).

Supplementary figures

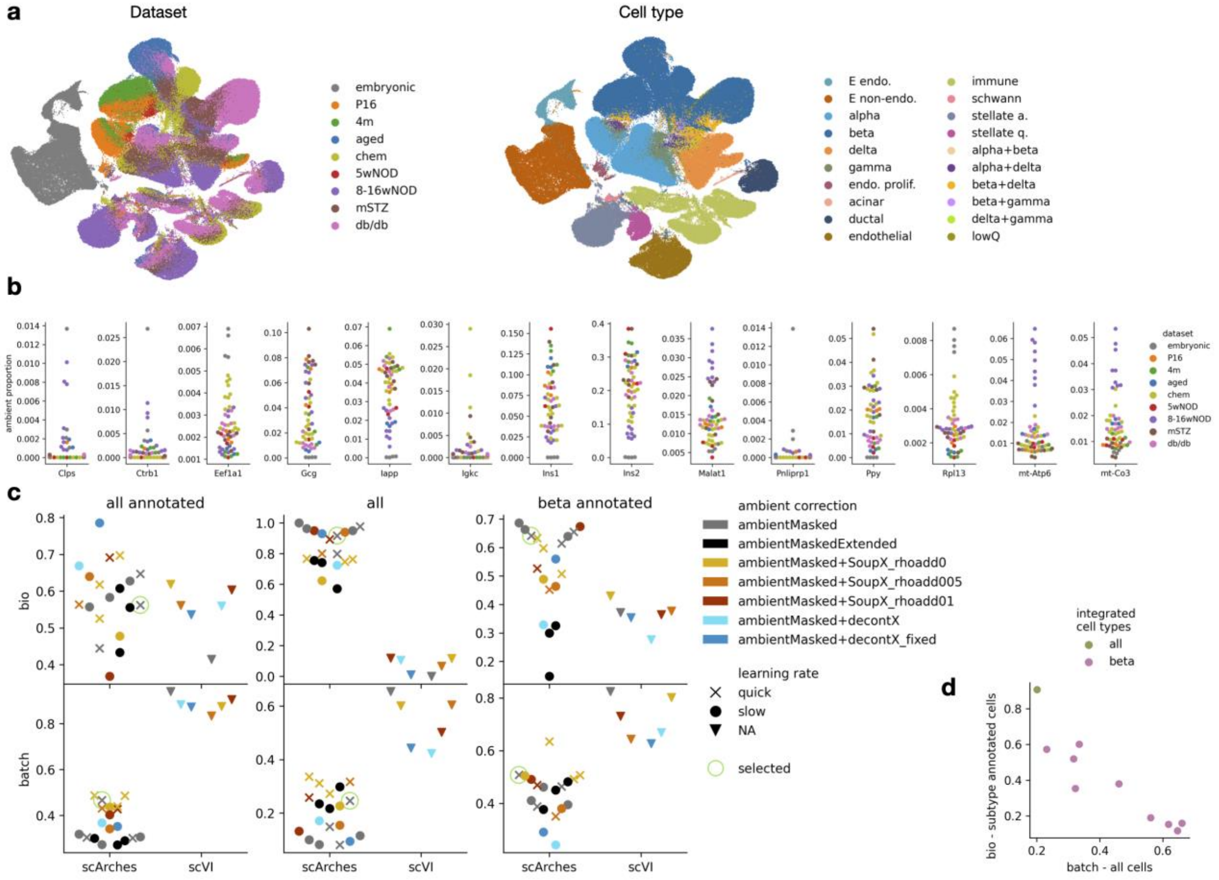

Supplementary Figure 1: Evaluation of atlas integration. (a) UMAP embedding of unintegrated atlas data. Cell types are colored based on annotation from integrated embedding for comparability to the final integrated atlas (**Figure 2d,e, Extended Data Figure 1a**). (b) Ambient expression strength of most ambient genes across samples. Ambient proportion denotes the proportion of counts within empty cells that belong to the gene. Each dot represents a sample colored by the dataset. (c) Integration metrics results for different integration protocols (dots) showing biological conservation (bio) and batch removal (batch) scores in scArches-cVAE and scVI integrations. The evaluation was performed on different data subsets: all annotated - cells from all cell types that had cell type annotation, which was used for evaluation (**Extended Data Figure 1b**); all - all cells, including unannotated, for batch removal evaluation we used clusters instead of cell types and for biological preservation evaluation we used only Moran's I; beta annotated - cells annotated as β (**Extended Data Figure 1b**), using marker-based cell states for evaluation. Some integration protocols were run multiple times, indicated by multiple dots of the same color and shape. Encircled integration is the one used for the atlas. We preprocessed data before integration with different ambient removal methods, including removal of top ambient genes (ambientMasked) or an extended list of ambient genes (ambientMaskedExtended), SoupX using default rho (rhoadd0) or increased rho (rhoadd005 - adding 0.05, rhoadd01 - adding 0.1), and DecontX using estimated delta or a pre-set delta (fixed) to increase the strength of ambient removal. We ran scArches-cVAE with two learning rate settings. (d) Integration metrics for β-cell specific integrations compared to metrics computed on β-cell embedding from the integration of all cells. We tuned β-cell integration strength, corresponding to multiple points on the graph.

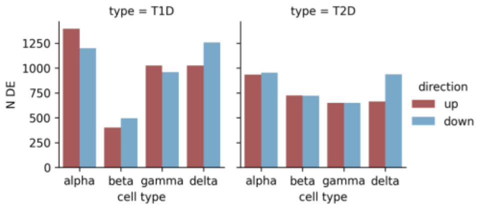

Supplementary Figure 2: All endocrine cell types exhibit expression changes in T1D (NOD) and T2D (db/db+mSTZ) models. Number of DEGs across cell types and diabetes models.

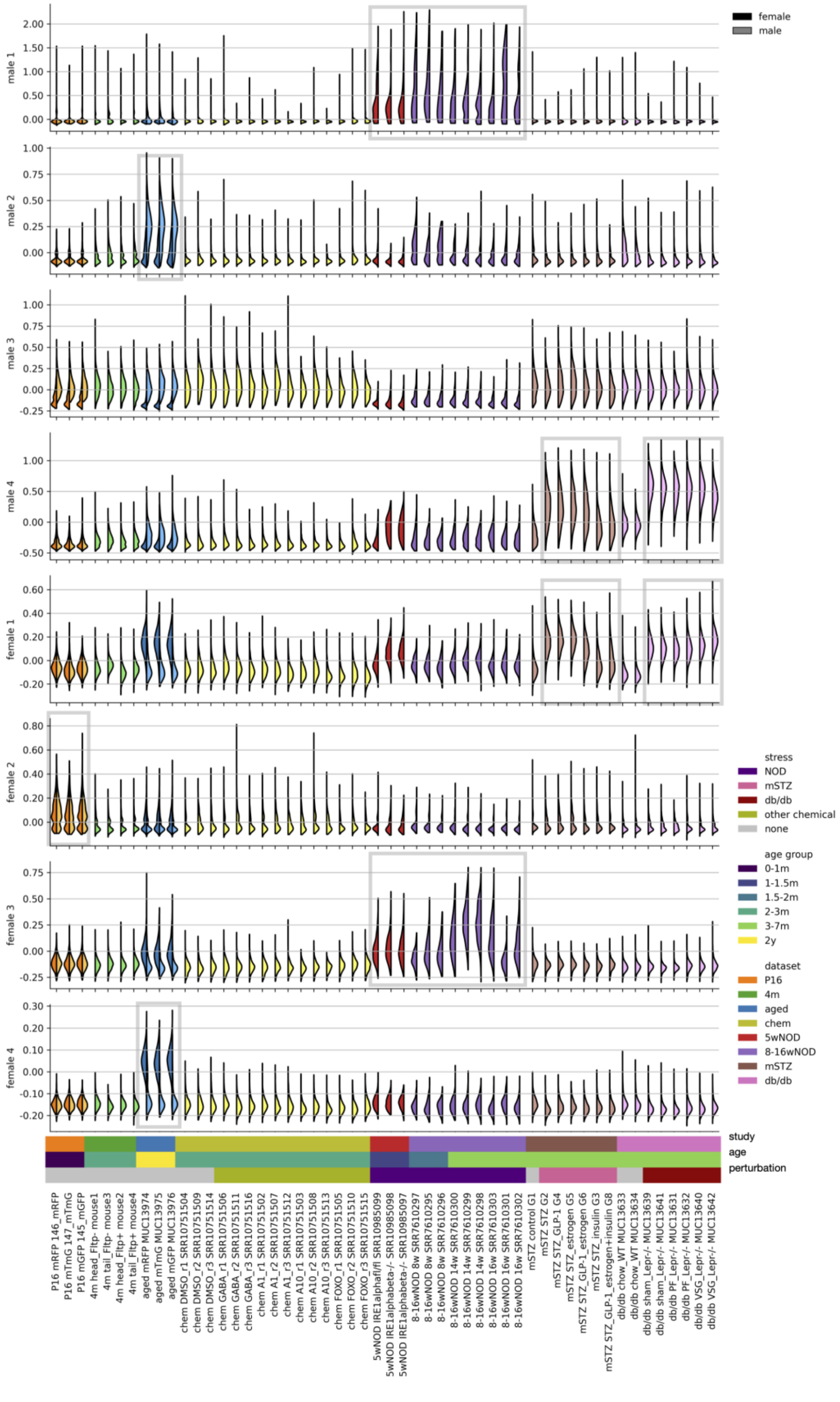

Supplementary Figure 3: Genes differentially expressed between aged male and female β-cells form distinct groups, some of which also show high activity in other conditions. The plot shows the activity of

gene groups across samples and sexes (indicated by the color darkness). Sample names are given as study_sampleDescription_sampleIdentifier.

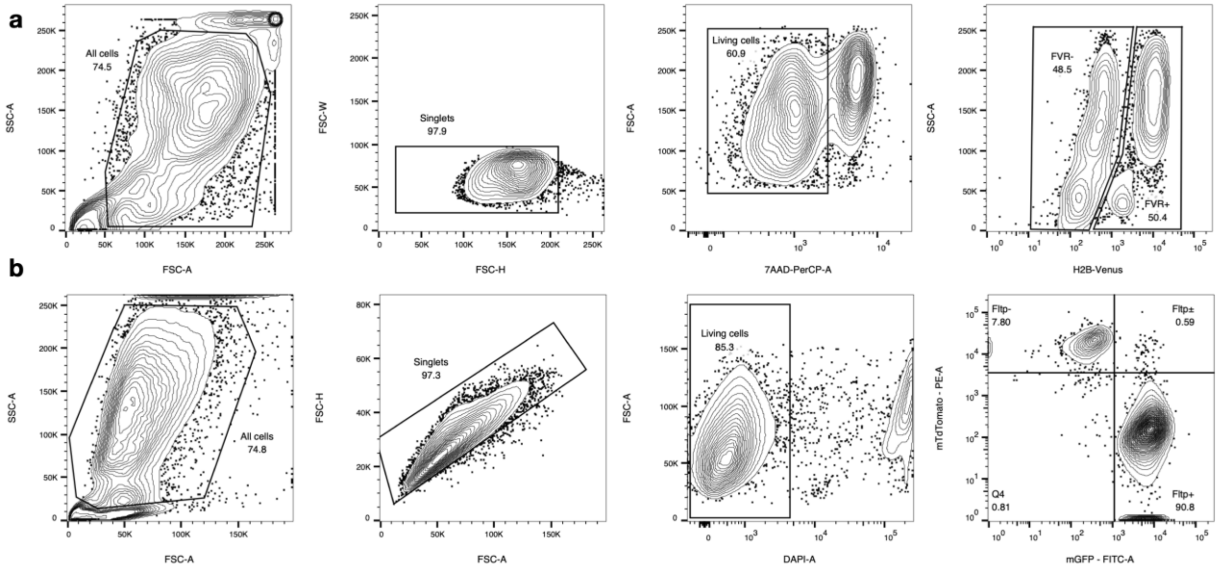

Supplementary Figure 4: FACS analysis of samples used for newly generated scRNA-seq datasets. Sorting strategy for (a) Fltp$^{ZV/+}$ and (b) Fltp iCre mTmG mouse strains. On plots are reported names and percentages of the used populations.

Supplementary table legends

Supplementary Table 1: Detailed description of individual samples from each dataset (given in separate sheets).

Supplementary Table 2: Number of cells per cell type (sheet name cell_type) and β-cell state (sheet names beta_subtype_coarse and beta_subtype_fine) within atlas samples. Samples in columns are named as "dataset sample_description sample_name". Sheet cell_type also contains columns: total - number of all cells within a cluster; low_quality - number of low-quality cells within a cluster, where blank/nan means that annotation of low-quality cells was not performed within that cluster; doublet - whether that cluster was assigned to contain doublets/multiplets.

Supplementary Table 3: Endocrine cell type markers in postnatal and embryonic data. On each sheet is given the result of a single developmental stage and cell type combination.

Supplementary Table 4: Results of human healthy (reference level) to T1D and T2D β-cell comparison for each dataset (sheets T1D_DE and T2D_DE, concatenated across datasets), gene set enrichment of genes consistently upregulated in human T1D or T2D β-cells, and comparison of gene set activity between healthy and dysfunctional mouse diabetes model β-cell groups for selected human diabetes-associated gene sets (sheet name mouse_genesets_DE). Column names for the DGE tables: dataset - dataset in which DGE was performed; n_group - n cells from diabetic samples; n_ref - n healthy cells; ratio_group - the ratio of cells from diabetic samples expressing the gene. Column names for the mouse comparison table: me_logFC - logFC on medians.

Supplementary Table 5: Results of coarse β-cell state marker identification. Column names: n_cl_de - number of clusters against which the marker was upregulated in the cluster of interest, min_lfc - minimal lFC across comparisons.

Supplementary Table 6: Genes present in GPs explaining variability across β-cells (sheet name GPs), their interpretation, gene set enrichment, and variance explained by selected diabetes-associated GPs in β-cells of healthy mouse and human samples (sheet name sample_explained_var). Column name explanation for the variance table: n_cells - n β-cells present in each sample.

Supplementary Table 7: β-cell heterogeneity markers (sheet beta_heterogeneity_markers) and function genes (sheet beta_function_genes) manually extracted from literature. Explanation of column names: gene_name - gene symbol of the associated organism, subtype - β-cell subtype with "NA" used when a specific subtype was not associated and "dedifferentiated" subtype also encompassing T2D models, organism - animal species for which marker was reported, function - molecular function associated with the gene, function_specific - a subcategory of function, reference - DOI of papers from which gene information was extracted.

Supplementary Table 8: Genes present in gene groups consistently variable across healthy adult β-cells (sheet name GPs), their interpretation, and gene set enrichment.

Supplementary Table 9: Results of DGE analysis between healthy and different diabetes-model groups across endocrine cell types; given in individual sheets, named as celltype_diabestype_DE, with T1D representing NOD model and T2D representing db/db+mSTZ models. Additionally, gene set enrichment of genes jointly up or downregulated across diabetes models in α-, γ-, and δ-cells (sheet names starting with sharedADG_DE and ending with DGE direction).

Supplementary Table 10: Results of DGE analysis and DEG clustering in 8-16wNOD (sheet T1D_DE) or db/db+mSTZ (Sheet T2D_DE) healthy (reference level) and diabetes model β-cells, gene set enrichment of individual DEG groups, DEG group interpretation, and gene set enrichment of DEGs in the same direction in db/db+mSTZ and 8-16wNOD DGE tests (sheet names starting with sharedT1DT2D_DE and ending with DGE direction).

Supplementary Table 11: Results of DGE analysis between male (reference level) and female β-cells across datasets (reported in sheets). The aged dataset also contains DEG groups named as down for male and up for female upregulated groups and their interpretation.

Supplementary Table 12: Detailed description of individual samples from each of the external validation datasets.

Supplementary Table 13: List of antibodies used in laboratory validation of diabetes markers.

Explanation of column names used in multiple tables: hc - gene group, if empty the gene was not assigned to any gene group; rel_beta_expr/rel_ct_expr - relative expression in β-cells/relevant cell type compared to other cell types; N_PMID - number of papers associated with a gene; N_PMID_pancreas_notCancerNonendo - as N_PMID, but filtering papers based on terms that indicate the paper is focused on endocrine pancreas compartment; mean_expr_in_expr_cells - mean expression in β-cells that have above zero expression.

Explanation of enrichment sheets: Sheet names containing the term "enrichment" represent gene set enrichment for individual gene groups. When enrichment analysis did not produce any significant results

for a specific gene group the corresponding enrichment sheet is empty. Columns: signature/query_size - N genes in gene group, geneset - N genes in the gene set, overlap - N overlapping genes, background - N genes in the background, hits - overlapping genes, recall - ratio overlap/geneset.

Explanation of gene group interpretation sheets: Sheet names containing the term “interpretation” contain interpretation for gene groups within the Excel file. Columns: group - group name, enrichment - manually curated summary of gene set enrichment, example_genes - example genes contained within the group, cell_cluster_expression - summary of expression pattern across β-cell states. Empty table cells indicate a lack of interpretation for given gene group characteristics.

References

1. Luecken, M. D., Büttner, M., Chaichoompu, K., Danese, A., Interlandi, M., Mueller, M. F., Strobl, D. C., Zappia, L., Dugas, M., Colomé-Tatché, M. & Theis, F. J. Benchmarking atlas-level data integration in single-cell genomics. *Nat. Methods* **19,** 41–50 (2022).
2. Stuart, T., Butler, A., Hoffman, P., Hafemeister, C., Papalexi, E., Mauck, W. M., 3rd, Hao, Y., Stoeckius, M., Smibert, P. & Satija, R. Comprehensive Integration of Single-Cell Data. *Cell* **177,** 1888–1902.e21 (2019).
3. Heumos, L., Schaar, A. C., Lance, C., Litinetskaya, A., Drost, F., Zappia, L., Lücken, M. D., Strobl, D. C., Henao, J., Curion, F., Single-cell Best Practices Consortium, Schiller, H. B. & Theis, F. J. Best practices for single-cell analysis across modalities. *Nat. Rev. Genet.* 1–23 (2023).
4. Lotfollahi, M., Naghipourfar, M., Luecken, M. D., Khajavi, M., Büttner, M., Wagenstetter, M., Avsec, Ž., Gayoso, A., Yosef, N., Interlandi, M., Rybakov, S., Misharin, A. V. & Theis, F. J. Mapping single-cell data to reference atlases by transfer learning. *Nat. Biotechnol.* **40,** 121–130 (2022).
5. Lopez, R., Regier, J., Cole, M. B., Jordan, M. I. & Yosef, N. Deep generative modeling for single-cell transcriptomics. *Nat. Methods* **15,** 1053–1058 (2018).
6. Yang, S., Corbett, S. E., Koga, Y., Wang, Z., Johnson, W. E., Yajima, M. & Campbell, J. D. Decontamination of ambient RNA in single-cell RNA-seq with DecontX. *Genome Biol.* **21,** 57 (2020).
7. Young, M. D. & Behjati, S. SoupX removes ambient RNA contamination from droplet-based single-cell RNA sequencing data. *Gigascience* **9,** (2020).
8. Camunas-Soler, J., Dai, X.-Q., Hang, Y., Bautista, A., Lyon, J., Suzuki, K., Kim, S. K., Quake, S. R. & MacDonald, P. E. Patch-Seq Links Single-Cell Transcriptomes to Human Islet Dysfunction in Diabetes. *Cell Metab.* **31,** 1017–1031.e4 (2020).
9. Wang, Y. J., Schug, J., Won, K.-J., Liu, C., Naji, A., Avrahami, D., Golson, M. L. & Kaestner, K. H. Single-Cell Transcriptomics of the Human Endocrine Pancreas. *Diabetes* **65,** 3028–3038 (2016).
10. Fang, Z., Weng, C., Li, H., Tao, R., Mai, W., Liu, X., Lu, L., Lai, S., Duan, Q., Alvarez, C., Arvan, P., Wynshaw-Boris, A., Li, Y., Pei, Y., Jin, F. & Li, Y. Single-Cell Heterogeneity Analysis and CRISPR Screen Identify Key β-Cell-Specific Disease Genes. *Cell Rep.* **26,** 3132–3144.e7 (2019).
11. Sachs, S., Bastidas-Ponce, A., Tritschler, S., Bakhti, M., Böttcher, A., Sánchez-Garrido, M. A., Tarquis-Medina, M., Kleinert, M., Fischer, K., Jall, S., Harger, A., Bader, E., Roscioni, S., Ussar, S., Feuchtinger, A., Yesildag, B., Neelakandhan, A., Jensen, C. B., Cornu, M., Yang, B., Finan, B., DiMarchi, R. D., Tschöp, M. H., Theis, F. J., Hofmann, S. M., Müller, T. D. & Lickert, H. Targeted pharmacological therapy restores β-cell function for diabetes remission. *Nat Metab* **2,** 192–209 (2020).
12. Oppenländer, L., Palit, S., Stemmer, K., Greisle, T., Sterr, M., Salinno, C., Bastidas-Ponce, A., Feuchtinger, A., Böttcher, A., Ansarullah, Theis, F. J. & Lickert, H. Vertical sleeve gastrectomy triggers fast β-cell recovery upon overt diabetes. *Mol Metab* **54,** 101330 (2021).

13. Bastidas-Ponce, A., Tritschler, S., Dony, L., Scheibner, K., Tarquis-Medina, M., Salinno, C., Schirge, S., Burtscher, I., Böttcher, A., Theis, F. J., Lickert, H. & Bakhti, M. Comprehensive single cell mRNA profiling reveals a detailed roadmap for pancreatic endocrinogenesis. *Development* **146,** (2019).
14. Salinno, C., Büttner, M., Cota, P., Tritschler, S., Tarquis-Medina, M., Bastidas-Ponce, A., Scheibner, K., Burtscher, I., Böttcher, A., Theis, F. J., Bakhti, M. & Lickert, H. CD81 marks immature and dedifferentiated pancreatic β-cells. *Mol Metab* **49,** 101188 (2021).
15. Marquina-Sanchez, B., Fortelny, N., Farlik, M., Vieira, A., Collombat, P., Bock, C. & Kubicek, S. Single-cell RNA-seq with spike-in cells enables accurate quantification of cell-specific drug effects in pancreatic islets. *Genome Biol.* **21,** 106 (2020).
16. Miranda, M. A., Macias-Velasco, J. F. & Lawson, H. A. Pancreatic β-cell heterogeneity in health and diabetes: classes, sources, and subtypes. *Am. J. Physiol. Endocrinol. Metab.* **320,** E716–E731 (2021).
17. Zeng, C., Mulas, F., Sui, Y., Guan, T., Miller, N., Tan, Y., Liu, F., Jin, W., Carrano, A. C., Huising, M. O., Shirihai, O. S., Yeo, G. W. & Sander, M. Pseudotemporal Ordering of Single Cells Reveals Metabolic Control of Postnatal β Cell Proliferation. *Cell Metab.* **25,** 1160–1175.e11 (2017).
18. Aguayo-Mazzucato, C., van Haaren, M., Mruk, M., Lee, T. B., Jr, Crawford, C., Hollister-Lock, J., Sullivan, B. A., Johnson, J. W., Ebrahimi, A., Dreyfuss, J. M., Van Deursen, J., Weir, G. C. & Bonner-Weir, S. β Cell Aging Markers Have Heterogeneous Distribution and Are Induced by Insulin Resistance. *Cell Metab.* **25,** 898–910.e5 (2017).
19. Bader, E., Migliorini, A., Gegg, M., Moruzzi, N., Gerdes, J., Roscioni, S. S., Bakhti, M., Brandl, E., Irmler, M., Beckers, J., Aichler, M., Feuchtinger, A., Leitzinger, C., Zischka, H., Wang-Sattler, R., Jastroch, M., Tschöp, M., Machicao, F., Staiger, H., Häring, H.-U., Chmelova, H., Chouinard, J. A., Oskolkov, N., Korsgren, O., Speier, S. & Lickert, H. Identification of proliferative and mature β-cells in the islets of Langerhans. *Nature* **535,** 430–434 (2016).
20. Lange, A., Gegg, M., Burtscher, I., Bengel, D., Kremmer, E. & Lickert, H. Fltp(T2AiCre): a new knock-in mouse line for conditional gene targeting in distinct mono- and multiciliated tissues. *Differentiation* **83,** S105–13 (2012).
21. Gegg, M., Böttcher, A., Burtscher, I., Hasenoeder, S., Van Campenhout, C., Aichler, M., Walch, A., Grant, S. G. N. & Lickert, H. Flattop regulates basal body docking and positioning in mono- and multiciliated cells. *eLife* **3,** Preprint at https://doi.org/10.7554/elife.03842 (2014)
22. Dorrell, C., Schug, J., Canaday, P. S., Russ, H. A., Tarlow, B. D., Grompe, M. T., Horton, T., Hebrok, M., Streeter, P. R., Kaestner, K. H. & Grompe, M. Human islets contain four distinct subtypes of β cells. *Nat. Commun.* **7,** 11756 (2016).
23. Thompson, P. J., Shah, A., Ntranos, V., Van Gool, F., Atkinson, M. & Bhushan, A. Targeted Elimination of Senescent Beta Cells Prevents Type 1 Diabetes. *Cell Metab.* **29,** 1045–1060.e10 (2019).
24. Eizirik, D. L., Pasquali, L. & Cnop, M. Pancreatic β-cells in type 1 and type 2 diabetes mellitus: different pathways to failure. *Nat. Rev. Endocrinol.* **16,** 349–362 (2020).
25. Xin, Y., Dominguez Gutierrez, G., Okamoto, H., Kim, J., Lee, A.-H., Adler, C., Ni, M., Yancopoulos, G. D., Murphy, A. J. & Gromada, J. Pseudotime Ordering of Single Human β-Cells Reveals States of Insulin Production and Unfolded Protein Response. *Diabetes* **67,** 1783–1794 (2018).
26. Lee, S., Zhang, J., Saravanakumar, S., Flisher, M. F., Grimm, D. R., van der Meulen, T. & Huising, M. O. Virgin β-Cells at the Neogenic Niche Proliferate Normally and Mature Slowly. *Diabetes* **70,** 1070–1083 (2021).
27. van der Meulen, T., Mawla, A. M., DiGruccio, M. R., Adams, M. W., Nies, V., Dólleman, S., Liu, S., Ackermann, A. M., Cáceres, E., Hunter, A. E., Kaestner, K. H., Donaldson, C. J. & Huising, M. O. Virgin Beta Cells Persist throughout Life at a Neogenic Niche within Pancreatic Islets. *Cell Metab.* **25,** 911–926.e6 (2017).
28. Huising, M. O., Lee, S. & van der Meulen, T. Evidence for a neogenic niche at the periphery of

pancreatic islets. *Bioessays* **40,** e1800119 (2018).
29. Yzydorczyk, C., Mitanchez, D., Boubred, F. & Simeoni, U. in *Glucose Intake and Utilization in Pre-Diabetes and Diabetes* (eds. Watson, R. R. & Dokken, B. B.) 5–20 (Academic Press, 2015).
30. Fasolino, M., Schwartz, G. W., Patil, A. R., Mongia, A., Golson, M. L., Wang, Y. J., Morgan, A., Liu, C., Schug, J., Liu, J., Wu, M., Traum, D., Kondo, A., May, C. L., Goldman, N., Wang, W., Feldman, M., Moore, J. H., Japp, A. S., Betts, M. R., HPAP Consortium, Faryabi, R. B., Naji, A., Kaestner, K. H. & Vahedi, G. Single-cell multi-omics analysis of human pancreatic islets reveals novel cellular states in type 1 diabetes. *Nat Metab* **4,** 284–299 (2022).
31. Burkhardt, D. B., Stanley, J. S., 3rd, Tong, A., Perdigoto, A. L., Gigante, S. A., Herold, K. C., Wolf, G., Giraldez, A. J., van Dijk, D. & Krishnaswamy, S. Quantifying the effect of experimental perturbations at single-cell resolution. *Nat. Biotechnol.* **39,** 619–629 (2021).
32. Richardson, S. J., Rodriguez-Calvo, T., Gerling, I. C., Mathews, C. E., Kaddis, J. S., Russell, M. A., Zeissler, M., Leete, P., Krogvold, L., Dahl-Jørgensen, K., von Herrath, M., Pugliese, A., Atkinson, M. A. & Morgan, N. G. Islet cell hyperexpression of HLA class I antigens: a defining feature in type 1 diabetes. *Diabetologia* **59,** 2448–2458 (2016).
33. Stožer, A., Šterk, M., Paradiž Leitgeb, E., Markovič, R., Skelin Klemen, M., Ellis, C. E., Križančić Bombek, L., Dolenšek, J., MacDonald, P. E. & Gosak, M. From Isles of Königsberg to Islets of Langerhans: Examining the Function of the Endocrine Pancreas Through Network Science. *Front. Endocrinol.* **13,** 922640 (2022).
34. Tonne, J. M., Sakuma, T., Deeds, M. C., Munoz-Gomez, M., Barry, M. A., Kudva, Y. C. & Ikeda, Y. Global gene expression profiling of pancreatic islets in mice during streptozotocin-induced β-cell damage and pancreatic Glp-1 gene therapy. *Dis. Model. Mech.* **6,** 1236–1245 (2013).
35. Cataldo, L. R., Vishnu, N., Singh, T., Bertonnier-Brouty, L., Bsharat, S., Luan, C., Renström, E., Prasad, R. B., Fex, M., Mulder, H. & Artner, I. The MafA-target gene PPP1R1A regulates GLP1R-mediated amplification of glucose-stimulated insulin secretion in β-cells. *Metabolism* **118,** 154734 (2021).
36. Green, C. D. & Olson, L. K. Modulation of palmitate-induced endoplasmic reticulum stress and apoptosis in pancreatic β-cells by stearoyl-CoA desaturase and Elovl6. *Am. J. Physiol. Endocrinol. Metab.* **300,** E640–9 (2011).
37. Hellemans, K. H., Hannaert, J.-C., Denys, B., Steffensen, K. R., Raemdonck, C., Martens, G. A., Van Veldhoven, P. P., Gustafsson, J.-A. & Pipeleers, D. Susceptibility of pancreatic beta cells to fatty acids is regulated by LXR/PPARalpha-dependent stearoyl-coenzyme A desaturase. *PLoS One* **4,** e7266 (2009).
