# Supplementary material for: Delineating mouse β-cell identity during lifetime and in diabetes with a single cell atlas
Source: Nat Metab. 2023 Sep 11;5(9):1615–37. doi: 10.1038/s42255-023-00876-x (PMC10513934; doi:10.1038/s42255-023-00876-x)
Supplement: Supplementary file 2 — Reporting Summary [file 42255_2023_876_MOESM2_ESM.pdf]

nature portfolio

Corresponding author(s): Heiko Lickert and Fabian Theis

Last updated by author(s): 5th of July, 2023

Reporting Summary

Nature Portfolio wishes to improve the reproducibility of the work that we publish. This form provides structure for consistency and transparency in reporting. For further information on Nature Portfolio policies, see our Editorial Policies and the Editorial Policy Checklist.

Statistics

For all statistical analyses, confirm that the following items are present in the figure legend, table legend, main text, or Methods section.

n/a	Confirmed	
☐	☒	The exact sample size (*n*) for each experimental group/condition, given as a discrete number and unit of measurement
☐	☒	A statement on whether measurements were taken from distinct samples or whether the same sample was measured repeatedly
☐	☒	The statistical test(s) used AND whether they are one- or two-sided<br>*Only common tests should be described solely by name; describe more complex techniques in the Methods section.*
☐	☒	A description of all covariates tested
☐	☒	A description of any assumptions or corrections, such as tests of normality and adjustment for multiple comparisons
☐	☒	A full description of the statistical parameters including central tendency (e.g. means) or other basic estimates (e.g. regression coefficient) AND variation (e.g. standard deviation) or associated estimates of uncertainty (e.g. confidence intervals)
☐	☒	For null hypothesis testing, the test statistic (e.g. F, t, r) with confidence intervals, effect sizes, degrees of freedom and P value noted<br>*Give P values as exact values whenever suitable.*
☒	☐	For Bayesian analysis, information on the choice of priors and Markov chain Monte Carlo settings
☒	☐	For hierarchical and complex designs, identification of the appropriate level for tests and full reporting of outcomes
☒	☐	Estimates of effect sizes (e.g. Cohen's d, Pearson's r), indicating how they were calculated

Our web collection on statistics for biologists contains articles on many of the points above.

Software and code

Policy information about availability of computer code

Data collection

For scRNA-seq no specific software was used, except for software associated with 10X Chromium Controller, NovaSeq6000 (Illumina), HiSeq4000 (Illumina), and Bioanalyzer. For FACS we used BD FACS ARIA III instrument and BD FACSDiva v6.1.3 software.

Data analysis

For the analysis of FACS data we used FlowJo v10.8.1 and for antibody staining images we used Leica Application Suite X (LAS X) v3.5.6 and ImageJ Fiji-Win32 software.

Reproducibility code and tutorial for mapping new data onto the atlas are available at https://github.com/theislab/mouse_cross-condition_pancreatic_islet_atlas.

Below is the list of most relevant software packages and their versions:
Python 3.8.5
R 4.0.2
10x Genomics Cell Ranger 2.2.1 – 3.1.0
anndata 0.7.4 – 0.7.6
CellBender 0.2.0
ComplexHeatmap 2.11.1
diffxpy 0.7.4
DecontX from celda v1.5
DropletUtils 1.10.3
edgeR 3.32.1
hypeR 1.6.0

Matplotlib 3.4.0
MELD 1.0.0
NumPy 1.19 – 1.22.3
pandas 1.0.5 – 1.3.2
rpy2 3.3.5
Scanpy 1.6 – 1.8.1
scArches 0.1.5
scIB developmental version last updated on 17. 1. 2022
SciPy 1.5.1 – 1.7.1
scran 1.16.0 – 1.18.7
Scrublet 0.2.1
scvi-tools 0.7.0a5
seaborn 0.11.1
SoupX 1.5.0

For manuscripts utilizing custom algorithms or software that are central to the research but not yet described in published literature, software must be made available to editors and reviewers. We strongly encourage code deposition in a community repository (e.g. GitHub). See the Nature Portfolio guidelines for submitting code & software for further information.

Data

Policy information about availability of data

All manuscripts must include a data availability statement. This statement should provide the following information, where applicable:

- Accession codes, unique identifiers, or web links for publicly available datasets
- A description of any restrictions on data availability
- For clinical datasets or third party data, please ensure that the statement adheres to our policy

Up-to-date data resource links are available from https://github.com/theislab/mouse_cross-condition_pancreatic_islet_atlas. The two newly generated scRNA-seq datasets, the integrated atlas, and the reference mapped embedding of the Feng dataset were deposited to GEO within super-series GSE211799. The atlas is also available as a cellxgene instance (https://cellxgene.cziscience.com/collections/296237e2-393d-4e31-b590-b03f74ac5070). The scArches model for reference mapping and an example code for reference mapping used for the Feng dataset are available in https://github.com/theislab/mouse_cross-condition_pancreatic_islet_atlas/tree/main/reference_mapping.
The following previously published datasets were included into the atlas: GSE132188, GSE161966, GSE128565, GSE174194, GSE144471, GSE117770, GSE142465 (GSM4228185 - GSM4228199). The following previously published datasets were used for validation: GSE83146, GSE137909, GSE148073, GSE81608, GSE198623, GSE81547, GSE86469, GSE124742 (FACS), GSE124742, GSE164875 (patch-seq), GSE101207, GSE154126, GSE83139. Gene sets were obtained from MSigDB (v7.4.1) and orthologue information from BioMart (Ensembl Genes v103).

Human research participants

Policy information about studies involving human research participants and Sex and Gender in Research.

Reporting on sex and gender	NA
Population characteristics	NA
Recruitment	NA
Ethics oversight	NA

Note that full information on the approval of the study protocol must also be provided in the manuscript.

Field-specific reporting

Please select the one below that is the best fit for your research. If you are not sure, read the appropriate sections before making your selection.

☒ Life sciences ☐ Behavioural & social sciences ☐ Ecological, evolutionary & environmental sciences

For a reference copy of the document with all sections, see nature.com/documents/nr-reporting-summary-flat.pdf

Life sciences study design

All studies must disclose on these points even when the disclosure is negative.

Sample size	We used all public datasets that have met our inclusion criteria (see Methods). No prior power calculations for sample size were performed. For scRNA-seq of aged mice, all subjects that reached the specified age were used. All newly generated scRNA-seq data contained at least 2 subjects per biological condition. Antibody staining was performed in at least 3 mice per condition, imaging at least 5 islets. A representative

picture was selected for the publication.

Data exclusions

In scRNA-seq datasets we removed low quality cells based on number of expressed genes, number of counts, and mitochondrial fraction by visual thresholding. We removed genes expressed in only a few cells. In downstream analyses we further excluded low quality cells based on clustering, technical cell multiplets based on expression of markers from multiple cell types, additional lowly expressed genes with more stringent filtering, and likely ambiently expressed genes based on expression in empty droplets and across cell types.

For details see Methods and reproducibility code https://github.com/theislab/mouse_cross-condition_pancreatic_islet_atlas.

Replication

We validated the reproducibility of many of our results based on external mouse scRNA-seq data. Some results could not be compared to external scRNA-seq datasets as no additional appropriate datasets were available. Most results were reproducible in the external data and where not we could explain the reasons for discrepancies, such as due to different cell states across conditions (see Results section).

Example endocrine markers and genes differentially expressed in diabetes were validated on protein level with antibody staining.

Randomization

Randomization was not performed. We added covariates to statistical tests as cofactors in regression models or performed tests on matched data subsets where no confounding by additional factors was expected.

Blinding

The generation of mouse data was not blinded, but we used standard protocols with all samples being treated equally. Data analysis was not blinded as the common analysis workflows require metadata availability across different steps, including evaluation, analysis of differences between groups, and biological interpretation.

Reporting for specific materials, systems and methods

We require information from authors about some types of materials, experimental systems and methods used in many studies. Here, indicate whether each material, system or method listed is relevant to your study. If you are not sure if a list item applies to your research, read the appropriate section before selecting a response.

Materials & experimental systems

n/a	Involved in the study	
☐	☒	Antibodies
☒	☐	Eukaryotic cell lines
☒	☐	Palaeontology and archaeology
☐	☒	Animals and other organisms
☒	☐	Clinical data
☒	☐	Dual use research of concern

Methods

n/a	Involved in the study	
☒	☐	ChIP-seq
☐	☒	Flow cytometry
☒	☐	MRI-based neuroimaging

Antibodies

Antibodies used

Primary antibody, Company, Order Number:
Insulin, Bio-Rad, 5330-0104G
Aldh1a3, Novus, NBP2-15339
Ucn3, Phoenix Pharmaceuticals, H-019-29
Nucb2, LIFE Technologies, PA578096
Fkbp11, Abcam, ab237528-100ug
Mt3, Abcam, ab214314-100ul
Transthyretin (TTR) / Prealbumin, Abcam, ab215202
RBP4, Abcam, ab109193
Somatostatin, Life-Tech, MA516987
Glucagon, Bio-Rad, 4660-1140
For all primary antibodies dilution IHC/IF 1-300 was used.

Secondary antibody (anti-), Company, Order Number, Label, Dilution IHC/IF:
Rabbit, Invitrogen, A11055, Alexa Flour® 488
Guinea pig, Dianova/Jackson, 706-165-148, Cy™3
Guinea pig, Dianova/Jackson, 706-495-148, Alexa Flour® 647
Goat, Dianova/Jackson, 705-605-147, Alexa Flour® 647
Rat, Dianova/Jackson, 712-605-150, Alexa Flour® 647
For all secondary antibodies dilution IHC/IF 1-800 was used.

Validation

We report previous publications or supplier web-pages with antibody validation:
Insulin https://www.bio-rad-antibodies.com/polyclonal/pig-porcine-insulin-antibody-5330-0054.html?f=purified&JSESSIONID_STERLING=EBBF4D2AEB04D4DAB38BB299F522226E.ecommerce2&evCntryLang=DE-dethirdPartyCookieEnabled, 10.1016/j.molmet.2021.101188
Aldh1a3 https://www.novusbio.com/products/aldh1a3-antibody_nbp2-15339, 10.1038/s42255-020-0171-3
Ucn3 https://www.phoenixpeptide.com/products/view/Antibodies/H-019-29, https://www.nature.com/articles/nbt.2141
Nucb2 https://www.thermofisher.com/antibody/product/NUCB2-Antibody-Polyclonal/PA5-78096, https://www.thermofisher.com/antibody/product/PA5-52551.html?gclid=CjwKCAiAqt-dBhBcEiwATw-ggKNvLkXj1uVFO-wSLearV2RPyiva0_JcmGhdAPHmAltdPU-

Xa6_AaxoCkc0QAvD_BwE&ef_id=CjwKCAiAqt-dBhBcEiwATw-ggKNvLkXj1uVFO-wSLearV2RPyiva0_JcmGhdAPHmAltdPU-Xa6_AaxoCkc0QAvD_BwE:G:s&s_kwcid=AL!3652!3!459736943987!!!g!!!10950825775!106531320406&cid=bid_pca_aup_r01_co_cp1359_pjt0000_bid00000_0se_gaw_dy_pur_con
Fkbp11 https://www.abcam.com/fkbp11-antibody-ab237528.html?productWallTab=ShowAll, 10.2147/OTT.S273823
Mt3 https://www.abcam.com/mt3-antibody-ab214314.html , 10.1038/s41598-021-84185-x
Ttr https://www.abcam.com/products/primary-antibodies/prealbumin-antibody-epr20971-ab215202.html
Rbp4 https://www.abcam.com/products/primary-antibodies/rbp4-antibody-ep3657-ab109193.html
Somatostatin https://www.thermofisher.com/antibody/product/Somatostatin-Antibody-clone-YC7-Monoclonal/MA5-16987
Glucagon https://www.bio-rad-antibodies.com/polyclonal/human-glucagon-antibody-4660-1140.html?f=purified&JSESSIONID_STERLING=E6E26457ECD7B7D3FA0B1AAA71BD7030.ecommerce1&evCntryLang=US-enthirdPartyCookieEnabled
Rabbit https://www.thermofisher.com/antibody/product/Donkey-anti-Goat-IgG-H-L-Cross-Adsorbed-Secondary-Antibody-Polyclonal/A-11055
Guinea pig 706-165-148 https://www.dianova.com/en/shop/706-165-148-donkey-igg-anti-guinea-pig-igg-hl-cy3-minx-bockgohshohumsrbrtsh/
Guinea pig 706-605-148 https://www.dianova.com/en/shop/706-605-148-donkey-igg-anti-guinea-pig-igg-hl-alexa-fluor-647-minx-bockgohshohumsrbrtsh/
Goat https://www.dianova.com/en/shop/705-605-147-donkey-igg-anti-goat-igg-hl-alexa-fluor-647-minx-ckgphshohumsrbrt/
Rat https://www.dianova.com/en/shop/712-605-150-donkey-igg-anti-rat-igg-hl-alexa-fluor-647-minx-bockgogphshohurbsh/

Animals and other research organisms

Policy information about studies involving animals; ARRIVE guidelines recommended for reporting animal research, and Sex and Gender in Research

Laboratory animals	Newly generated mouse samples:<br>scRNA-seq of mouse islets:<br>- Fltp lineage tracing line (Fltp iCre mTmG), older than 2 years, male and female<br>- Fltp reporter line (Fltp-ZV), aged 4 months, male<br>Antibody staining of mouse islets:<br>- C57BL/6J, P9 or aged 2-4 months, male and female<br>- db/db model (B6.BKS(D)-Leprdb/J, diabetic and healthy control), aged 8 weeks, male<br>- NOD model, aged 8 weeks, female
Wild animals	No wild animals were used.
Reporting on sex	We performed differential expression analysis between the sexes in the aged and P16 dataset animals where matched samples of both sexes were available. Sex differences were observed only among aged animals. Sex was determined on mice subjects or based on the expression of Y-linked genes in pooled samples.
Field-collected samples	No field-collected samples were used.
Ethics oversight	Animal studies were conducted with adherence to relevant ethical guidelines for the use of animals in research in agreement with German animal welfare legislation with the approved guidelines of the Society of Laboratory Animals (GV-SOLAS) and the Federation of Laboratory Animal Science Associations (FELASA). The study was approved by the Helmholtz Munich Animal Welfare Body and by the Government of Upper Bavaria.

Note that full information on the approval of the study protocol must also be provided in the manuscript.

Flow Cytometry

Plots

Confirm that:

- [x] The axis labels state the marker and fluorochrome used (e.g. CD4-FITC).
- [x] The axis scales are clearly visible. Include numbers along axes only for bottom left plot of group (a 'group' is an analysis of identical markers).
- [x] All plots are contour plots with outliers or pseudocolor plots.
- [x] A numerical value for number of cells or percentage (with statistics) is provided.

Methodology

Sample preparation	Dissociated islets of Langerhans were washed and resuspended in FACS buffer (2 % FCS, 2 mM EDTA in PBS). Prior sorting, cells were passed through 40 µm cell strainer to remove possible clumps.
Instrument	Single cells were analyzed and sorted by FACS-Aria III (BD) with a 100 µm nozzle.
Software	Data were analyzed with the FACS DIVA software v6.1.3 and FlowJo v10.8.1.

Cell population abundance	Abundance of Fltp populations (from living cells = 60-90%) are not reported in this manuscript since they vary according to age and mouse model utilized, thus going beyond the purpose of the paper.
Gating strategy	First, islets' cells were gated using FSC-A and SCA-A to retain the majority of the endocrine cells. Second, doublets were excluded using FSC-H and FSC-W (FSC-W high excluded) or FSC-A and FSC-H (excluding cells below the diagonal). Third, dead cells were gated out using a viability dye (DAPI or 7-AAD positive cells excluded). Last, for the FltpiCre mTmG cells, the endogenous membrane EGFP and membrane TdTomato fluorescent proteins were used to distinguish the three Fltp populations: mEGFP+ (Fltp positive), mTdtomato+ (Fltp negative), mEGFP+ and mTdtomato+ double positive (Fltp transient). These three populations were sorted separately and used for the scRNAseq experiment. For the Fltp Venus Repeorter (FVR) cells, the endogenous H2B-Venus was used to distinguish cells positive and negative for the Fltp

☒ Tick this box to confirm that a figure exemplifying the gating strategy is provided in the Supplementary Information.
